# LC-MS Fingerprinting Development for Standardized Precipitate from *Agastache mexicana*, Which Induces Antihypertensive Effect through NO Production and Calcium Channel Blockade †

**DOI:** 10.3390/pharmaceutics15092346

**Published:** 2023-09-19

**Authors:** Karla Catalina Cruz-Torres, Samuel Estrada-Soto, Luis Arias-Durán, Gabriel Navarrete-Vázquez, Julio César Almanza-Pérez, Beatriz Mora-Ramiro, Irene Perea-Arango, Emanuel Hernández-Núñez, Rafael Villalobos-Molina, Gabriela Carmona-Castro, Irma-Martha Medina-Díaz, Gabriela Ávila-Villarreal

**Affiliations:** 1 Facultad de Farmacia, Universidad Autónoma del Estado de Morelos, Cuernavaca 62209, Morelos, Mexico; karla.cruzto@uaem.edu.mx (K.C.C.-T.); gabriel_navarrete@uaem.mx (G.N.-V.); 2 Ciencias de la Salud, Universidad Autónoma Metropolitana-Iztapalapa, Iztapalapa, Ciudad de Mexico 09340, Mexico; adl_ff@uaem.mx (L.A.-D.); j.almanza.perez@gmail.com (J.C.A.-P.); hibrida24@gmail.com (B.M.-R.); 3 Centro de Investigación en Biotecnología, Universidad Autónoma del Estado de Morelos, Cuernavaca 62209, Morelos, Mexico; iperea@uaem.mx (I.P.-A.); gabbyca18@yahoo.com (G.C.-C.); 4 Departamento de Recursos del Mar, Centro de Investigación y de Estudios Avanzados del IPN, Unidad Mérida, Mérida 97310, Yucatán, Mexico; emanuel.hernandez@cinvestav.mx; 5 Unidad de Biomedicina, Facultad de Estudios Superiores Iztacala, Universidad Nacional Autónoma de Mexico, Tlalnepantla 54090, Estado de Mexico, Mexico; rafaelvillalobosmolina@gmail.com; 6 Laboratorio de Contaminación y Toxicología Ambiental, Secretaría de Investigación y Posgrado, Universidad Autónoma de Nayarit, Tepic 63000, Nayarit, Mexico; irmartha.md@uan.edu.mx; 7 Centro Nayarita de Innovación y Transferencia de Tecnología “Unidad Especializada en I+D+i en Calidad de Alimentos y Productos Naturales”, Universidad Autónoma de Nayarit, Tepic 63000, Nayarit, Mexico; 8 Unidad Académica de Ciencias Químico Biológicas y Farmacéuticas, Universidad Autónoma de Nayarit, Tepic 63000, Nayarit, Mexico

**Keywords:** *Agastache mexicana*, acacetin, antihypertensive, NO production, oleanolic acid, tilianin, ursolic acid

## Abstract

The aim of this work was to evaluate the vasorelaxant and antihypertensive effects of a standardized precipitate of the hydroalcoholic extract from *Agastache mexicana* (PP*Am*), comprising ursolic acid, oleanolic acid, acacetin, luteolin and tilianin, among others. In the ex vivo experiments, preincubation with L-NAME (nonspecific inhibitor of nitric oxide synthases) reduced the relaxation induced by PP*Am*; nevertheless, preincubation with indomethacin (nonspecific inhibitor of cyclooxygenases) did not generate any change in the vasorelaxation, and an opposed effect was observed to the contraction generated by CaCl_2_ addition. Oral administration of 100 mg/kg of PP*Am* induced a significant acute decrease in diastolic (DBP) and systolic (SBP) blood pressure in spontaneously hypertensive rats, without changes in heart rate. Additionally, PP*Am* showed a sustained antihypertensive subacute effect on both DBP and SBP for 10 days compared to the control group. On the other hand, human umbilical vein cells treated with 10 µg/mL of PP*Am* showed a significant reduction (*p* < 0.05) in intracellular adhesion molecule-1, compared to the control, but not on vascular cell adhesion molecule-1. In conclusion, PP*Am* induces a significant antihypertensive effect in acute- and subacute-period treatments, due to its direct vasorelaxant action on rat aortic rings through NO production and Ca^2+^ channel blockade.

## 1. Introduction

Hypertension is a chronic disease characterized by persistent increased blood pressure (BP) in the systemic arteries, with values over 130 mmHg for systolic BP (SBP) and over 80 mmHg for diastolic BP (DBP) [1]. The hypertension is a risk factor for developing renal dysfunction, cerebrovascular disease, heart failure, and stroke. This has a global epidemiological and economic impact [2,3,4]. Even though there are many antihypertensive drugs such as diuretics, vasodilators, sympatholytic, calcium-channel blockers, and drugs that inhibit the renin-angiotensin system, these are often not devoid of side effects and non-adherence to therapy is a common issue among patients [3,4,5]. Thus, it is necessary to search for new drugs from medicinal plants to offer more specific and successful treatments for patients who have hypertension. 

In Mexico, the medicinal plant, *Agastache mexicana* (kunth.) lint. & epling *subsp mexicana*, commonly called “toronjil morado” has been used as a traditional remedy for various diseases, including insomnia, high blood pressure, digestive disorders, and diabetes, among others [6]. The Agastache genus produces a diverse range of secondary metabolites, including volatile and non-volatile compounds. Most of these metabolites belong to the phenylpropanoid and terpenoid classes. *A. mexicana* in particular, is rich in terpenoid compounds such as monoterpenes, sesquiterpenes, diterpenes, and triterpenes, including ursolic, oleanolic, corosolic, and maslinic acids. Furthermore, phenolic and phenylpropanoid compounds such as flavones, primarily acacetin, and flavonoids such as tilianin and hesperitin, are also present in significant amounts. [7]

Through our previous studies, we were able to establish that dichloromethanic (DE*Am*) and methanolic (ME*Am*) extracts showed a significant vasorelaxant effect, in a concentration-dependent and partially endothelium-dependent manner. From DE*Am*, acacetin, ursolic acid (UA), and oleanolic acid (OA) [8,9], and from ME*Am* tilianin [10,11] were obtained. The compounds mentioned showed vasorelaxant effect mainly via NO production. Moreover, tilianin induced a significant decrease in both SBP and DBP on spontaneously hypertensive rats (SHR) in acute studies, and the activity was dose-dependent and did not produce any toxicological effects [12]. All these previous results led us to develop a standardized precipitate (PP*Am*), which was obtained via hydroalcoholic extraction from *A. mexicana*. The PP*Am* containing acacetin, luteolin, tilianin, UA, and OA, was previously evaluated with regard to its vasorelaxant action and antihypertensive effects, using in vitro, ex vivo*,* and in vivo approaches. 

The standardization of crude plant extracts, precipitates, or fractions is an imperative subject when such extracts are used for medicinal purposes. Consequently, the development of fast and effective analytical methods for chemical content fingerprinting of plant extracts is of high interest. Liquid chromatography with electrospray mass spectrometry (ESI-MS) instrumentation allowed the development of relatively standardized metabolite profiling via fingerprinting procedures to assign, align, and annotate peaks correctly [13,14].

One of the best alternatives to acquire information about a metabolic profile from standardized products is conducting a mass scan experiment, and a selected ion recording experiment for each compound mass expected [13]. Within this frame of reference, the aim of this study was to evaluate the vasorelaxant action and antihypertensive effect of characterized PP*Am* using in vitro, ex vivo*,* and in vivo methodologies linked to its metabolic profile.

## 2. Materials and Methods

### 2.1. Chemicals and Drugs

*N*-nitro-L-arginine methyl ester (L-NAME), noradrenaline hydrochloride (NA) ≥ 98%, indomethacin, carbamoylcholine chloride (carbachol) ≥ 98%, luteolin, acacetin, nifedipine, UA and OA were purchased from Sigma-Aldrich Co. (St. Louis, MO, USA). Tilianin was previously isolated from *A. mexicana* [10].

### 2.2. Plant Material

*A. mexicana* was collected in the flowering season in October 2019, in Tlalnepantla, Morelos, Mexico (19°06′6.65″ N and 98°55′49.3″ W, at 2823 m above sea level). The plant was identified by Dr. Irene Perea Arango of CEIB-UAEM, and a voucher specimen (No 35766) was deposited in the HUMO Herbarium of the Universidad Autónoma del Estado de Morelos.

### 2.3. Preparation of the Extract

Collected plants were dehydrated inside a room; once dried, the material was pulverized in a mechanical mill; this allowed substantial contact with the extraction solvent. Two thousand grams of *A. mexicana* were macerated with aqueous–ethanol mixture (30:70) (hydroalcoholic extract, HAE*Am*) over 72 h, repeating this step twice more. Then, the solvent was removed on a rotary evaporator until reaching near-dryness, and a mixture of compounds (PP*Am*) was obtained via spontaneous precipitation from the liquid hydroalcoholic extract.

### 2.4. Chemical Fingerprinting Development by UPLC-MS Analysis

Ultra-performance liquid chromatography coupled with mass spectrometry (UPLC-ESI-MS) method was developed using an ACQUITY UPLC^®^ H-Class Bio System (Waters^®^ Corp., Milford, MA, USA). To optimize the chromatographic conditions and achieve suiTable separations and detections, we conducted experiments using two different columns and assessed both isocratic and gradient mobile phases. The separation was conducted using an ACQUITY UPLC^®^ HSS T3 130 Å column (1.8 µm, 2.1 × 100 mm with a column temperature of 40 °C and with an ACQUITY UPLC^®^ HBE C18 130 Å column (1.7 µm, 2.1 × 50 mm). With the aim of selecting a mobile phase ideal for identifying flavonoids and pentacyclic triterpenoid acids in the same experiment, two methods were assessed.

The elution process involved the use of a binary solvent system and isocratic mode. Isocratic elution consisted of (A) 0.05% aqueous ammonium hydroxide (20%), and (B) acetonitrile (80%), at a flow rate of 0.4 mL/min for 10 min. For gradient elution, the solvent system employed consisted of (A) 0.05% aqueous ammonium hydroxide, and (B) acetonitrile for ESI−, (C) 0.1% aqueous formic acid, and (B) acetonitrile for ESI+, both at a flow rate of 0.4 mL/min. Gradient elution for ESI−, was the following: 0–5 min, 90 to 10% (A), 5–7 min, 10% (A), 7–8 min, 10 to 90% (A), and 8–10 min, 90% (A). For ESI+, solvent A was replaced by C. For all cases, 3 μL of the samples at 100 ppm concentration were injected. Either acetonitrile or methanol was employed as the solvent for both the samples and the blank.

Detection was carried out with a single-quadrupole mass detector (QDa) spectrometer with an electrospray ionization source (ESI) (Waters^®^ Corp., Milford, MA, USA). ESI interface was used in positive and negative ion mode. Voltage of the capillary-tube was put to 1.5 kV and −0.8 kV for positive and negative-ion mode, respectively (ESI+, ESI−). The data were processed using Waters Empower™ 3 software (Waters^®^ Corp., Milford, MA, USA). Total ion chromatogram (TIC) mass scan acquisition was set to 50–1250 Da and a selected ion recording (SIR) for each one targeted mass was obtained [15,16].

### 2.5. Rat Aorta Rings and Functional Studies

Wistar male rats of 250–300 g were euthanized, and the thoracic aorta was dissected, freed of surrounding fat, and cut into 4–5 mm rings. Endothelium was removed in some arterial rings via gentle rubbing of the intima layer. Aortas were hooked in the bottom of the chamber and in an isometric Grass-FT03 force transducer (Astromed, West Warwick, RI, USA), connected to a MP100 analyzer (Biopac^®^ Instruments, Santa Barbara, CA, USA), as described [12]. The optimal tension for rings was set to 3 g and bathed in 10 mL of Krebs–Henseleit mixture (in mM: NaCl, 118; KCl, 4.7; CaCl_2_, 2.5; MgSO_4_, 1.2; 157 KH_2_PO_4_, 1.2; NaHCO_3_, 25.0; and glucose, 11.1, pH 7.4), kept at 37 °C and bubbled with oxygen (O_2_/CO_2_, 19:1). Once equilibrated, aortas were subjected to contraction via noradrenaline (NA, 0.1 µM) along 10 min, then washed out to remove the agonist; this was repeated two more times after 30 min each was over. Lack of relaxation with carbachol (1 µM) after the last contraction allowed us to confirm the absence of endothelium. After the final NA-induced contraction, the extract was added in a volume of 100 µL; then, concentration–response assays were conducted for each arterial ring, the relaxation was compared before and after the addition of extracts, using Acknowledge 3.8.1 software (Biopac^®^) [17]. To evaluate the vasorelaxant action due to PP*Am*, two approaches were followed: in endothelium containing aortic rings, enzyme inhibitors of endothelium signaling pathways were used, as follows: 15 min before last NA-induced contraction, aortic rings were exposed to L-NAME 175 (10 μM, nitric oxide synthases inhibitor), or indomethacin (10 μM, cyclooxygenases inhibitor), accordingly [18,19]. Then, concentration–response curves of extract-induced relaxation were obtained. In the second approach, the concentration–response curves to CaCl_2_ (80 μM–27 mM) were constructed for the control group, or after 15 min incubation with 100 and 314 μg/mL of the extract. CaCl_2_-induced contractility was compared without (control group) and with the PP*Am* [20,21].

### 2.6. ICAM and VCAM Determination in HUVEC Cells

#### 2.6.1. Cell Viability by Neutral Red Assay

Viability of HUVEC cells was determined via the amount of neutral red (Sigma-Aldrich) incorporated, which is proportional to the number of viable cells. Cells were plated in 24-well dishes at semi-confluency (40,000 cells/well), then treated with PP*Am* (0.1, 1, 10 and 100 μg/mL) for 24 h, at 37 °C in a humidified atmosphere with 5% CO_2_. Afterward, the cells were exposed for 2 h to neutral red; then, cells were read at 540 nm.

#### 2.6.2. Cytoplasmic and Nuclear Protein Extracts

HUVEC were exposed to PP*Am* (10 μg/mL) for 1 h before being stimulated with LPS (1 μg/mL) (Escherichia coli 0111:B4, Sigma-Aldrich) for 3 h. Then, cells were washed twice with cold PBS and scrape-harvested in PBS. After, the cells were spun at 330× *g* for 5 min to obtain the pellet, which was frozen in dry ice for 1 min, and gently resuspended in 100 μL of hypotonic solution (10 mM Hepes pH 7.9, 10 mM KCl, 1.5 mM MgCl_2_, 1 mM DTT), and spun at 3300× *g* at 4 °C; the supernatant was collected to obtain cytoplasmic proteins. The pellet containing cell nuclei was re-suspended in 50 μL of hypertonic solution (10 mM Hepes pH 7.9, 400 mM NaCl, 1.5 mM MgCl_2_, 25% glycerol, 0.2 mM EDTA, 1 mM DTT, 0.5 mM PMSF), incubated for 30 min, mixed gently at 4 °C, and spun at 1340× *g* for 10 min. These supernatants with nuclear proteins were collected and diluted 1:1 with HDKE buffer (20 mM Hepes pH 7.9, 50 mM KCl, 25% glycerol, 0.2 mM EDTA, 1 mM DTT, 0.5 mM of PMSF), and stored at −70 °C for further use. Cytoplasmic and nuclear protein concentrations were determined according to Bradford method.

#### 2.6.3. Western Blotting

The cytoplasmic extract was split in a 10% SDS-polyacrylamide gel and transferred to a polyvinylidene fluoride membrane (Bio-Rad, Mexico), with a glycine transfer buffer (192 mM glycine, 25 mM Tris-HCl (pH 8.8), and 10% methanol (*v*/*v*) overnight). The next day, the nonspecific sites were blocked with TBS-Tween 1%-albumin 5%. After overnight incubation, membranes were exposed to specific primary antibody VCAM (1:500, Santa Cruz Biotechnology, Inc., Dallas, TX, USA); ICAM (1:1000 Santa Cruz), and β-actin (1:10,000 222 Santa Cruz), diluted in TBS-Tween 1%-albumin 1% at 4 °C. Thereafter, membranes were exposed to secondary antibodies (anti-rabbit-HRP (1:20,000 Pierce-Thermo, Waltham, MA, USA) for 1 h, and anti-mouse-HRP (1:10,000 Pierce Thermo)), at room temperature. Proteins were detected via chemiluminescence “Super-Signal” System (Pierce, Rockford, IL, USA). The generated signals were detected with the Fusion FX Vilber Lourmat device (VILBER Smart Imaging, PerkinElmer, Waltham, MA, USA), the spots were densitometrically quantified.

### 2.7. Acute and Subacute In Vivo Antihypertensive Experiments

Antihypertensive activity study of PP*Am* was conducted in SHR rats (250–300 g). Animals were divided into four groups (seven animals each): SHR Control (10% Tween 80, group 1), SHR treated (PP*Am* 100 mg/kg, group 2), SHR amlodipine (reference drug 5 mg/kg, group 3), and Wistar Kyoto rats (WKY, normotensive control, group 4). Measurements (SBP, DBP, and heart rate) were recorded before and after intragastric administration (i.g.) dose of test samples at 0, 1, 3, 5 and 7 h by a tail-cuff method, using a LE 5001 automatic blood pressure meter (PanLabTM Harvard Apparatus, Barcelona, Spain). Percent decrease in HR, SBP, and DBP was calculated [12]. 

After this test, the subacute antihypertensive test was conducted, where the previous four groups were used. Measurements were recorded before and after i.g. administration dose of PP*Am* (100 mg/kg/day), or amlodipine (5 mg/kg/day) at 0, 1, 3, 7, and 10 days. Subsequently, the animals of the subacute antihypertensive study were sacrificed, and the aortic rings of each group were used to construct concentration–response curves for relaxation (carbachol), following the methodology detailed above.

### 2.8. Acute Oral Toxicity

To determine the possible acute lethal effect of PPAm at different dosages, an acute oral toxicity evaluation was conducted. The assessment of acute oral toxicity was determined according to the modified OECD 423 guideline. Male CD1 mice strains were randomly selected, marked to permit individual identification, and kept in their cages for 5 days prior to dosing to allow for acclimatization to the laboratory conditions [22].

For the experiment, five cohorts of three mice each were subjected to an 8 h fasting period with ad libitum access to water. Every cohort was subjected to administration of the PP*Am* at varying doses: 5, 50, 300, and 2000 mg/kg, alongside a control group receiving the vehicle only. The response of the treated mice was carefully monitored over a span of 72 h, during which the incidence of mortality was recorded for each dose. Animals were observed for 14 days following the administration of the substance. Observations focused on skin changes, body hair, somatomotor activity, and regular behavior. Significant attention was directed towards an assortment of manifestations, such as tremors, convulsions, diarrhea, lethargy, and sleep. The fatalities were subsequently classified and characterized in accordance with the guidelines outlined by the Globally Harmonized System of Classification and Labeling (GHS) [23].

### 2.9. Statistical Analysis 

The experimental results were presented as the mean ± standard error of the mean (SEM), and statistical significance among the groups was assessed using a one-way analysis of variance (ANOVA) followed by Tukey’s multiple comparison test. A significance level of *p* ≤ 0.05 was deemed as statistically significant.

## 3. Results and Discussion

### 3.1. Chemical Characterization of HAEAm and PPAm by LC-MS

The outcome of the maceration process applied to *Agastache mexicana* revealed a yield of 1.8% for the hydroalcoholic extract (HAE*Am*) and 0.73% for the precipitate (PP*Am*). Subsequent to extraction, the obtained extracts underwent thorough pharmacological analysis and were also subjected to the development of fingerprinting for further characterization.

**Figure 1:**
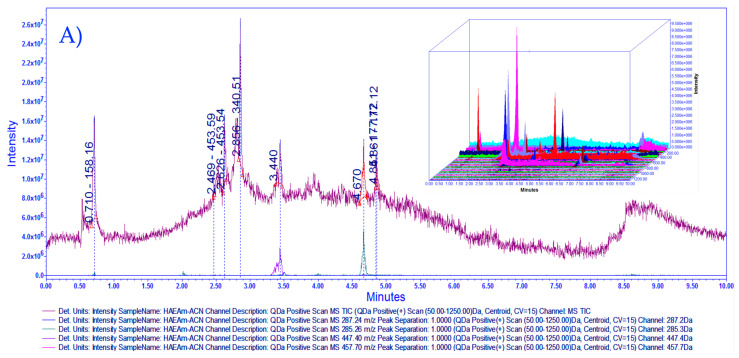

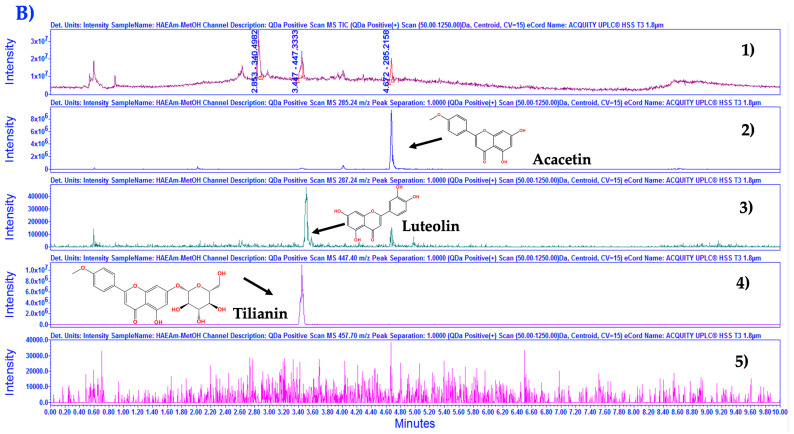
(**A**) HAE*Am* Overlayed chromatograms for total ion chromatogram (TIC) with mass scan 50.0–1250 Da and selected channels for identification of 285.26 Da acacetin, 287.2 Da luteolin, 447.4 tilianin, 457.70 oleanolic acid and ursolic acid with 3D chromatogram. (**B**) Stack plot for HAE*Am* (1) TIC and selected channels from mass scan at (2) 285.26 Da *m*/*z* [M+1] for acacetin identification, peak retention time 4.66, (3) 287.24 Da *m*/*z* [M+1] for luteolin identification, peak retention time 3.49, (4) 447.40 Da *m*/*z* [M+1] for tilianin peak retention time 3.43. (5) Oleanolic acid and ursolic acid, mass not detected. Acquired in positive mode [ESI+] gradient elution; the x-axis represents time, and the y-axis represents signal intensity for 3D chromatogram z-axis represent mass in Da. Column ACQUITY UPLC^®^ HSS T3 130 Å (1.8 µm, 2.1 × 100 mm). Column ACQUITY UPLC^®^ HSS T3 130 Å (1.8 µm, 2.1 × 100 mm). For additional details, complete retention time, and mass data for Figure 1A,B, please refer to Supplementary Material Table S1.

Liquid chromatography-mass spectrometry (LC-MS) fingerprinting has emerged as a powerful analytical tool for the targeted identification and quantification of compounds in traditional herbal medicines, particularly for the targeted analysis of bioactive compounds in mixtures. The chemical characterization and fingerprinting of *Agastache mexicana* presents a unique challenge due to the presence of multiple classes of compounds in the herbal preparation. Following a series of preliminary experiments, it became evident that the column ACQUITY UPLC^®^ HSS T3 130 Å (1.8 µm, 2.1 × 100 mm) showcased better performance, making it the ideal choice for the development of fingerprinting analysis. In Figure 1, we can observe the complexity of the mixture; the major peaks indicate the presence of flavonoids acacetin, luteolin, and tilianin; other peaks present in HAE*Am* were not identified. As observed in the 3D chromatogram, the diversity of masses is extensive, and it is possible that signal intensities overlap, thereby complicating their elucidation.

**Figure 2:**
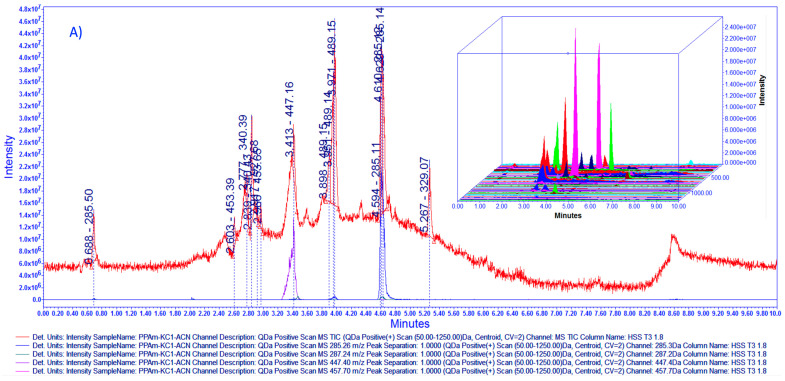

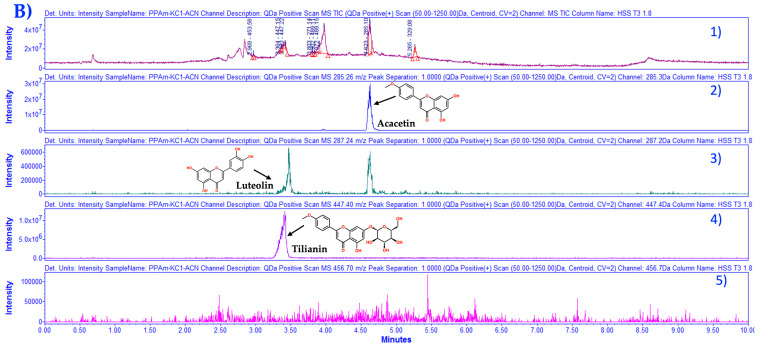

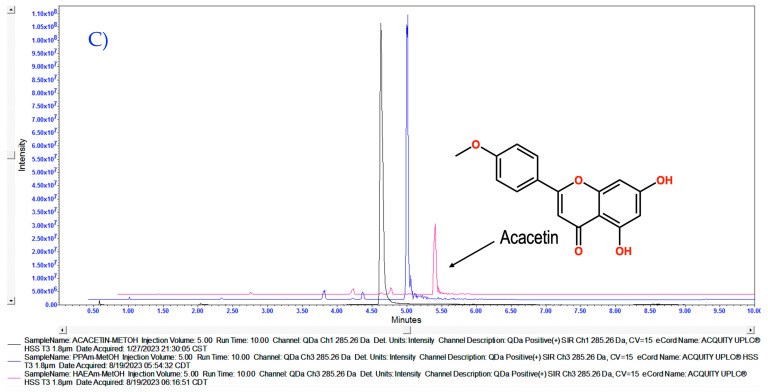

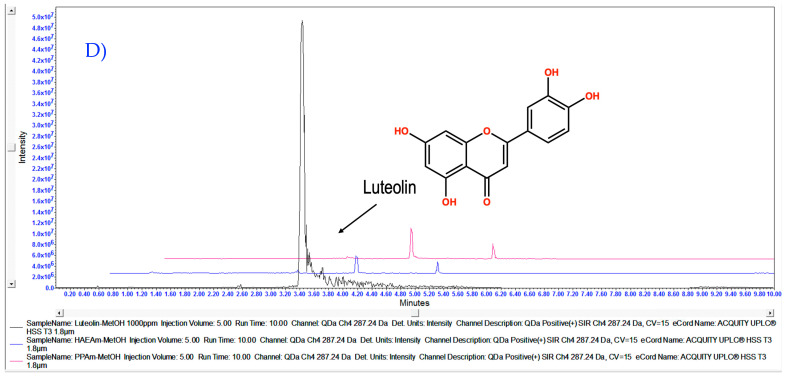

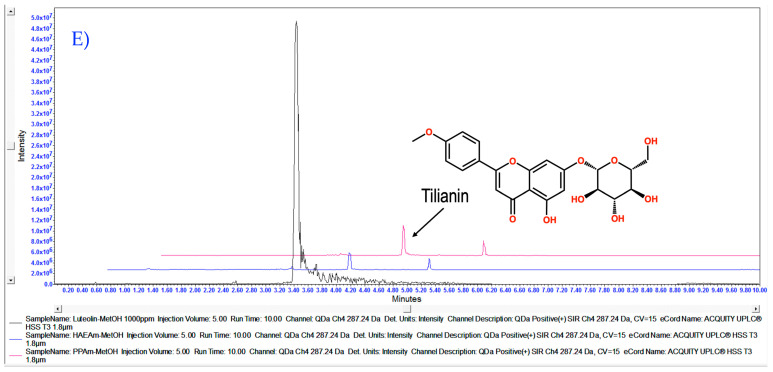
(**A**) Overlaid chromatograms for PP*Am* TIC with mass scan 50.0–1250 Da and selected channels for identification of 285.26 Da acacetin, 287.2 Da luteolin, 447.4 tilianin, 457.70 oleanolic acid and ursolic acid with 3D Chromatogram. (**B**) Stack plot for PP*Am* (1) TIC and selected channels from mass scan at (2) 285.26 Da *m*/*z* [M+1] for acacetin identification, peak retention time 4.66, (3) 287.24 Da *m*/*z* [M+1] for luteolin identification, peak retention time 3.49, (4) 447.40 Da *m*/*z* [M+1] for tilianin peak retention time 3.43. (5) Oleanolic acid and ursolic acid, mass not detected. (**C**–**E**) show the overlaid chromatogram for acacetin, luteolin and tilianin, respectively comparing standard ant 1000 ppp against HAE*Am* and PP*Am* at 100 ppm. Acquired in positive mode [ESI+] gradient elution; the x-axis represents time, and the y-axis represents signal intensity for 3D chromatogram z-axis represent mass in Da. Column ACQUITY UPLC^®^ HSS T3 130 Å (1.8 µm, 2.1 × 100 mm). For additional details, complete retention time, and mass data for Figure 2A,B, please refer to Supplementary Material Table S1.

As described in the experimental section, during vacuum concentration of liquid hydroalcoholic extract (HAE*Am*), a yellowish amorphous solid was obtained via spontaneous precipitation (PP*Am*), which was characterized via LC-MS. Fingerprinting of PP*Am* was established using LC-MS-ESI (Figure 2). From this, we can observe the presence of acacetin, tilianin, luteolin, UA, and OA, where acacetin was found in greater proportion followed by luteolin, tilianin, UA and OA. The selectivity of the analytical method was guaranteed through the use of the selected ion recording (SIR) acquisition mode, monitoring the *m*/*z* ratio corresponding to the protonated molecule [M + H]^+^ for the target analyte. After quantification based on compared intensity with standards at 1000 ppm, we determined the compound percentages for HAE*Am* and PP*Am*. As shown in Table 1, the major compound in PP*Am* with a 31.443% of acacetin/1 g of PP*Am* (314.43 µg/1 mg).

**Figure 3:**
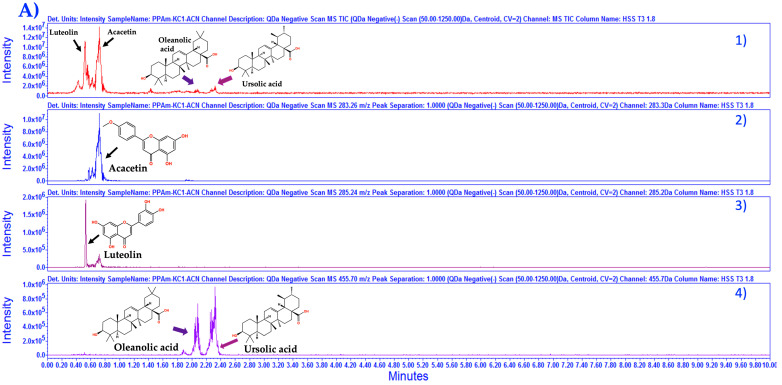

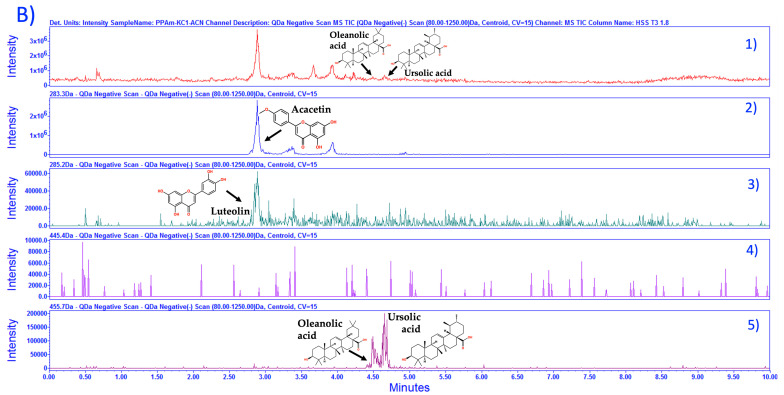
(**A**) Stack plot for PP*Am*, (1) TIC with mass scan 50.0–1250 Da and selected channels for identification with 3D chromatogram. Selected channels: (2) 283.26 Da *m*/*z* [M−1] for acacetin identification, peak retention time 0.718, (3) 285.24 Da *m*/*z* [M−1] for luteolin identification, peak retention time 0.521, (4) 455.7 Da *m*/*z* [M−1] for oleanolic acid and ursolic acid, peak retention time 2.27 and 2.35, respectively. Acquired in negative mode [ESI−] isocratic elution. (**B**) Stack plot for PP*Am* (1) TIC and selected channels from mass scan at (2) 283.26 Da *m*/*z* [M−1] for acacetin identification, peak retention time 2.87 (3) 285.24 Da *m*/*z* [M−1] for luteolin identification, peak retention time 2.81, (4) 447.40 Da *m*/*z* [M−1] for tilianin peak retention time not identified. (5) Oleanolic acid and ursolic acid, peak retention time 4.5 and 4.65. Chromatograms acquired in negative mode [ESI−] gradient elution, the x-axis represents time, and the y-axis represents signal intensity. Column ACQUITY UPLC^®^ HSS T3 130 Å (1.8 µm, 2.1 × 100 mm).

The identification for OA and UA was improved. In Figure 3, the fingerprinting for PP*Am* through isocratic and gradient elution in negative acquisition mode (ESI−) is presented, as we can observe isocratic elution was more efficient to separate OA and UA as previously reported [24].

It is imperative to note that in a previous study, acacetin and OA, and a mixture of acacetin/OA/UA in the dichloromethanic extract from *A. mexicana* (DE*Am*) were obtained, using bio-guided fractionation. The structural elucidation of these compounds was conducted through NMR [8]. Furthermore, it is noteworthy that tilianin was not found in DE*Am*. For comprehensive information and in-depth insights, please refer to the Supplementary Materials, where further details regarding the analysis are extensively documented.

### 3.2. Vasorelaxant Effect of Hydroalcoholic Extract (HAEAm) and PPAm from Agastache mexicana

**Figure 4:**
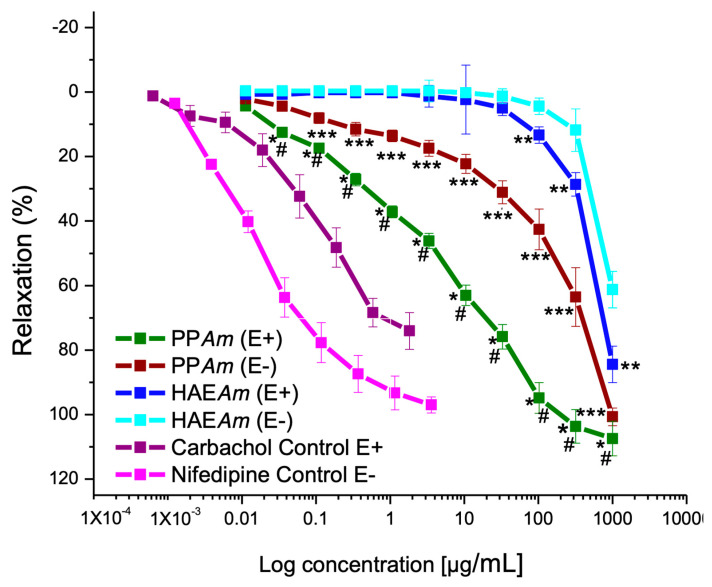
Concentration–response curves of the relaxing effect induced by HAE*Am* and PP*Am* on isolated rat aortic rings pre-contracted with NA, in the presence and absence of endothelium. Results are expressed as mean ± standard error of the mean (SEM) from six experiments. Statistical significance is indicated as follows: * *p* < 0.05 PP*Am* (E+) versus PP*Am* (E−), ** *p* < 0.05 HAE*Am* (E+) versus HAE*Am* (E−), # *p* < 0.05 PP*Am* (E+) versus HAE*Am* (E+), and *** *p* < 0.05 HAE*Am* (E−) versus PP*Am* (E−); (E+ indicates tissue with endothelium; E− indicates the absence of endothelium in the tissue).

As observed in Figure 4 and Table 2, HAE*Am* showed a significant vasorelaxant effect on contraction induced by NA (0.1 µM), and the effect was partially endothelium-dependent. As described in the experimental section, during liquid HAE*Am* concentration, a mixture of some compounds was precipitated, which contained UA and OA, tilianin, luteolin, and acacetin, among others. This precipitate (PP*Am*) was also evaluated in aortic rings with and without endothelium, where PP*Am* was more potent and efficient than the hydroalcoholic extract, and the relaxation was better in the presence of endothelium—almost similar in potency than carbachol but more efficient. It is important to note that the vasorelaxant effect of UA and OA [8,25], acacetin [8], luteolin, and tilianin [10] was previously determined. Their effect was significant and totally or partially endothelium-dependent, which explains that the mixture of compounds elicited excellent relaxation and a drastic enhancement for relaxant activity.

### 3.3. Functional Mechanism of Vasorelaxant Action of PPAm

Thus, the fact that the effect was partially endothelium-dependent suggests that the mechanism of action is related to the release of endothelium-derived relaxing factors, such as NO, H_2_S, PGI_2_ or endothelium-derived hyperpolarizing factor [8,19]. On the other hand, the relaxing component that does not depend on the endothelium could be related to the blocking or opening of ion channels, or to the interaction with second messengers [10,20].

**Figure 5:**
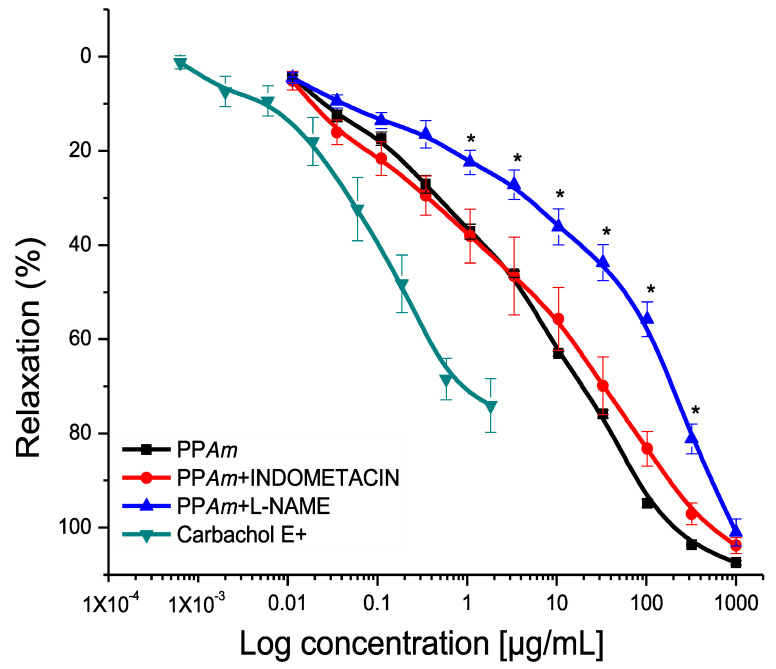
Concentration–response curves of the relaxant effect induced by PP*Am* on isolated rat aortic rings with endothelium, in the presence of Indomethacin or L-NAME contracted by NA. Results are expressed as the means ± SEM of six experiments (* *p* < 0.05 vs. Control).

With these results, we decided to establish the mechanism of action of PP*Am* in the presence of endothelium. Figure 5 shows the partial blocking of the relaxing activity induced by L-NAME, observed by the shift to the right on the concentration–response–relaxant curve of PP*Am* in the presence of the inhibitor, where the potency decreased significantly with respect to the control curve. Furthermore, the concentration-dependent effect is appreciated. These results indicate that the effect of PP*Am* was reduced in the absence of NO, suggesting that eNOS participates in the pharmacological effect observed [10,19]. On the other hand, with indomethacin, there was no significant difference compared to the control, indicating that the precipitate did not induce the synthesis of PGl_2_ via COX.

**Figure 6:**
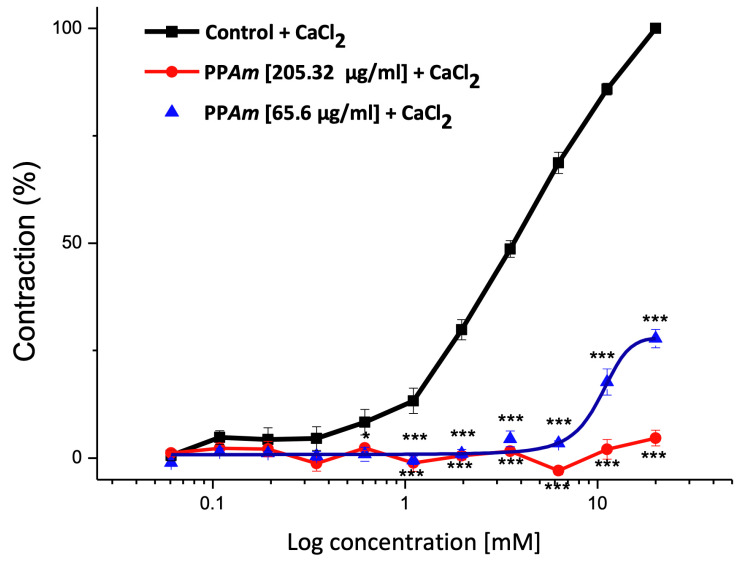
Inhibitory effects of PP*Am* on the cumulative contraction curves, dependent on extracellular Ca^2+^ influx in Ca^2+^-free solution. Data are expressed as means ± SEM of six experiments (* *p* < 0.05, ***p < 0.001).

As mentioned, the relaxant effect induced by PP*Am* was partially endothelium-dependent, and the participation of endothelium via NO production was corroborated. Then, it was necessary to explore the relaxant mechanism related to the smooth muscle cells. Thus, the potential blockade of L-type calcium channels was studied. Figure 6 shows that PP*Am* (65.6 and 205.32 µg/mL) significantly diminished CaCl_2_-induced contraction in a concentration-dependent manner, suggesting the blockade of Ca*^2^*^+^ flows into smooth muscle cells [8].

### 3.4. Acute and Subacute Antihypertensive Effects of Hydroalcoholic Extract (HAEAm) and PPAm from Agastache mexicana

**Figure 7:**
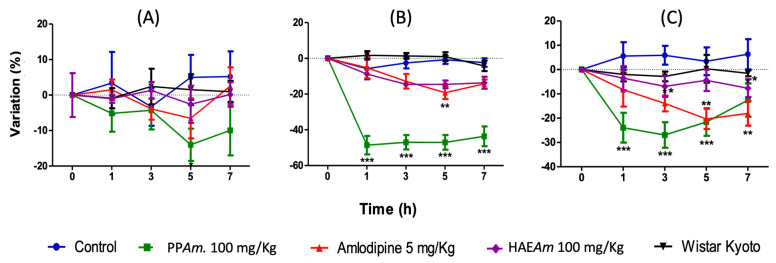
Maximal decrease in (**A**) heart rate, (**B**) systolic blood pressure, and (**C**) diastolic blood pressure (%) elicited by PP*Am* (100 mg/kg) intragastric administration on SHR acute test, the X-axis represents time in hours, and Y-axis represents variation in percentage. Results are expressed as means ± SEM, *n* = 6 rats per group, * *p* < 0.05, ** *p* < 0.01 and *** *p* < 0.001 vs. Control.

Based on the previous results, it was decided to evaluate the antihypertensive effects of HAE*Am* and PP*Am* in vivo on SHR. As depicted in Figure 7, HAE*Am* exhibited significant antihypertensive activity at a dose of 100 mg/kg, leading to a significant reduction in SBP and DBP after 1 h, which was maintained for 7 h. However, there was no significant modification observed in HR. Additionally, PP*Am* showed a strong and significant reduction in both SBP and DBP compared to the SHR and Wistar Kyoto control groups. The antihypertensive effect at a dose of 100 mg/kg was found to be more potent than amlodipine, a potent antihypertensive drug commonly used in the therapeutic management of hypertension (5 mg/kg, positive control) (*p* < 0.05).

In addition, it is important to mention that heart rate was not modified after extract and PP*Am* treatments (Figure 7). 

Since PP*Am* showed significant activity on DBP, it suggests that the mixture of compounds present there has a significant influence on decreasing blood vessel resistance (due to the NO production and calcium channel blockade), influencing the regulation of blood pressure and mitigating the risk of cardiovascular events.

These results are consistent with previous studies that show the responsible antihypertensive compounds present in dichloromethane (acacetin, UA and OA) and methanol (tilianin) extracts of *A. mexicana* [8,10].

Therefore, it is suggested that the biological effects of HAE*Am* and PP*Am* are linked to these flavonoids and terpenoids. Overall, these findings indicate that PP*Am* obtained from HAE*Am* could serve as an important agent for the development of antihypertensive drugs with potential clinical uses. Further research can be conducted to explore the therapeutic potential of these compounds and to develop more effective treatments for hypertension and related cardiovascular diseases.

**Figure 8:**
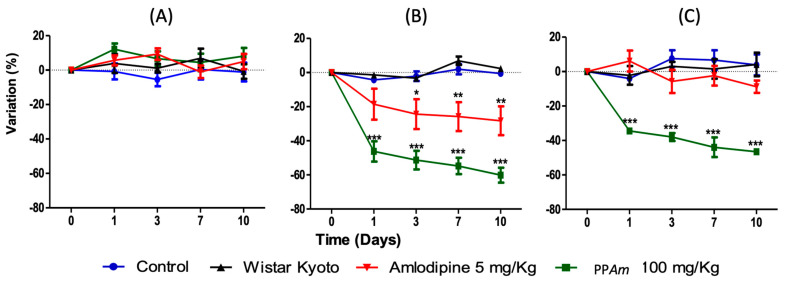
Maximal decrease in (**A**) heart rate, (**B**) systolic blood pressure and (**C**) diastolic blood pressure (%) elicited by intragastric administration of PP*Am* (100 mg/Kg) on SHR subacute test; X axis represents time in days, and Y axis represents variation in percentage. Results are expressed as means ± SEM; *n* = 6 rats per group; * *p* < 0.05, ** *p* < 0.01 and *** *p* < 0.001 vs. Control.

After the significant acute antihypertensive effect shown by test samples, we decided to conduct the subacute antihypertensive study in SHR. As can be seen, a notable decrease in SBP and DBP was induced by PP*Am* (100 mg/kg/day) (Figure 8) from the first day, and this effect was sustained until the tenth day (*p* < 0.001), compared with vehicle and WKY groups (*p* < 0.05). Again, the effect of PP*Am* was more pronounced than that shown by amlodipine (5/mg/kg/day). This activity can be attributed to the compounds that have already been identified in this precipitate, which are UA and OA, tilianin, luteolin, and acacetin [8], and this is based on antecedents described for each compound [8,9,10,25]. 

**Figure 9:**
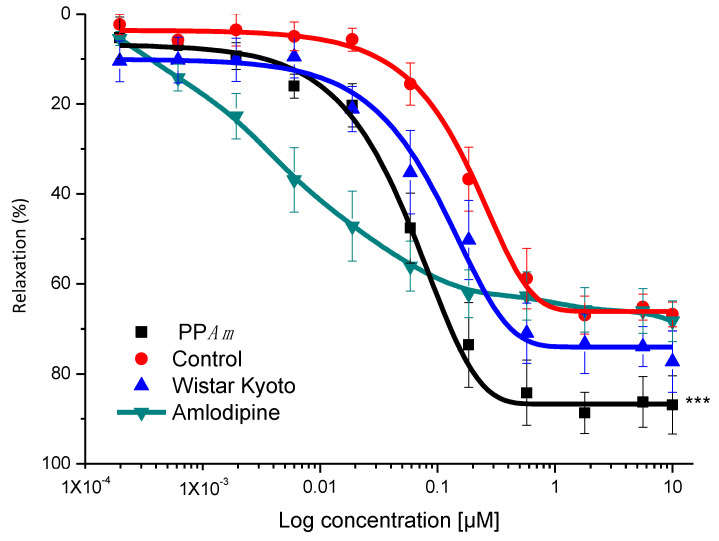
Concentration–response curves of the relaxant effect induced by carbachol on isolated rat aortic rings with endothelium (precontracted with NA 0.1 µM) obtained from SHR and WKY after subacute treatment with test samples. Results are expressed as the means ± SEM of six; *n* = 6 experiments; *** *p* < 0.001.

However, the confluence of all these compounds in a single mixture favors a synergistic response in antihypertensive activity over that shown by each of the compounds when they were individually studied in previous experiments. After SHR and WKY subacute treatment, animals were sacrificed, and aortic rings were obtained and placed on organ tissue baths with physiological solution. Once tissues were NA contracted, carbachol relaxant curves were constructed, which allowed us to observe that daily cumulative administration of PP*Am* on hypertensive rats improved the relaxing response of the tissue, compared to the aortas of untreated hypertensive rats, and even better than WKY control normotensive rats (Figure 9), suggesting that treatment with PP*Am* could restore the functionality of the aorta of hypertensive animals.

**Figure 10:**
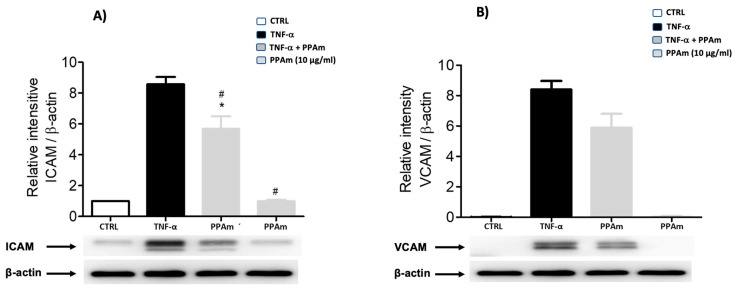
Effect of PP*Am* on ICAM (**A**) and VCAM (**B**) protein levels in HUVEC cells after stimulation with TNF-α for 180 min. The total cellular extracts were subjected to Western blot analysis. Relative intensity values were normalized using β-actin and were expressed relative to the control (CTRL; cell cultures without treatment). Values are presented as means ± SEM of three independent experiments. * *p* < 0.05 compared to control, # *p* < 0.05 compared to TNF-α.

This effect may be related to the prevention of atheroma plaque formation, since treatment of HUVEC cells with PP*Am* significantly decreased ICAM cell adhesion and has a non-significant tendency to decrease VCAM (Figure 10).

These results establish the pharmacological basis to carry out other preclinical assessments (such as in vitro and in vivo, acute, and sub-chronic toxicity studies), that make it possible to set up this standardized mixture as a potential phytopharmaceutical agent for the development of an antihypertensive drug.

The present study provides evidence that selected bioactive compounds present in *Agastache mexicana* and in PP*Am*, possess significant vasorelaxant and antihypertensive effects. PP*Am* contains a mixture of bioactive compounds, including acacetin [7,8], luteolin [7,26,27], and tilianin) [10], as well as pentacyclic triterpenic acids such as ursolic acid and morolic acid (in lower amounts) [8]. Flavonoids are known to exhibit a wide range of biological activities, including antioxidant, anti-inflammatory, and antihypertensive effects. Acacetin has been shown to inhibit vasoconstriction and reduce blood pressure in hypertensive rats, while luteolin has been found to have a vasodilatory effect and improve endothelial function. Tilianin has also been reported to have hypotensive effects and may act as a calcium channel blocker [7,8].

On the other hand, pentacyclic triterpenic acids such as ursolic and morolic acids have been shown to exhibit potent antihypertensive effects by reducing vascular resistance and improving endothelial function. Ursolic acid has been reported to induce vasorelaxation and reduce blood pressure by enhancing the production of nitric oxide (NO) and reducing oxidative stress. Morolic acid has also been found to have hypotensive effects and may act as a calcium channel blocker [28,29].

The synergistic effects of these bioactive compounds in PP*Am* are likely responsible for the significant vasorelaxant and antihypertensive effects observed in the present study. The fact that PP*Am* showed partial endothelium dependency in its vasorelaxant activity through the production of NO and calcium channel blockade suggests that it may have multiple mechanisms of action, which is advantageous for the treatment of hypertension [7,8,9,10,12].

### 3.5. Acute Oral Toxicity

Behavioral observation of the test animals after dosing did not show skin changes in body hair, somatomotor activity, and regular behavior. Seizures and tremors were not observed in the animals treated with PPAm, nor was there any lethality 72 h after the treatment was administered. 

**Figure 11:**
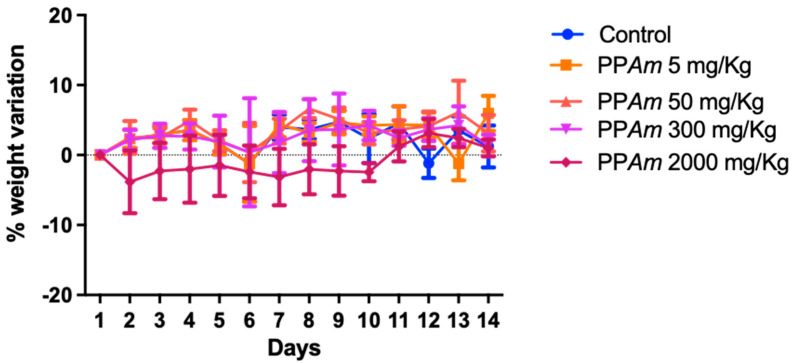
Effects of the extract on body weight of mice in acute toxicity study. Values are presented as means ± SEM; *n* = 3.

Over the 14-day period of observation after single oral acute toxicity evaluation, normal food and water intake were observed, accompanied by non-significant fluctuations in body weight. These findings suggest the unimpeded operation of lipid, carbohydrate, and protein metabolism within the animals. This is noteworthy as these nutrients play pivotal roles in various physiological functions of the organism. The body weights of test animals of both control and groups treated with PP*Am* were increased progressively throughout the study period as showed in Figure 11. These results allowed us to classify the *PPAm* in group 5 (LD50 > 2000 mg/kg), falling in lower a toxicity class according to the Globally Harmonized Classification System (mg/kg b.w) [23].

## 4. Conclusions

*Agastache mexicana* is widely used in Mexican traditional medicine for the treatment of various ailments, including hypertension. The present study provides scientific evidence to support the traditional use of this medicinal plant for hypertension and highlights the potential of its bioactive compounds as a source of new antihypertensive drugs. 

PP*Am* showed significant vasorelaxant activity through partial endothelium-dependency, through the production of NO and Ca^2+^ channel blockade as well. In the in vivo model, PP*Am* had a significant and sustained acute and sub-acute antihypertensive effect in SHR rats at a dose of 100 mg/Kg. Further studies are needed to determine the optimal dose and long-term effects of PP*Am* on blood pressure regulation and its potential as a therapeutic agent for hypertension. The preliminary results of acute oral toxicity indicated an absence of toxicity. Therefore, conducting a subchronic oral toxicity assessment may be considered.

## Figures and Tables

**Table 1:** Concentration of bioactive compounds in *HAEAm* and *PPAm.* Results are expressed in parts per million (ppm).

	Acacetin	Luteolin	Tilianin
HAE*Am*	19.20	3.88	6.36
PP*Am*	31.23	6.28	17.65

**Table 2:** Pharmacological parameters of the relaxant effects induced by HAE*Am* and PP*Am* on aortic rings with (E+) and without (E−) endothelium on the contraction induced by NA.

	HAE*Am* (E−)	HAE*Am* (E+)	PP*Am* (E−)	PP*Am* (E+)	Carbachol	Nifedipine
**E_max_ (%)**	61.5 ± 2.8	84.0 ± 3.6	100.0 ± 2.7	100.0 ± 0.4	74.1 ± 5.7	97 ± 2.5
**CE_50_ (µg/mL)**	503.9 ± 1.9	425.8 ± 3.6	157.3 ± 9.1	4.5 ± 0.7	0.082 ± 6.7	0.01 ± 3.4

## Data Availability

Further details regarding the analysis are extensively documented in Supplementary Materials.

## Supplementary Materials

The following supporting information can be downloaded at: https://www.mdpi.com/article/10.3390/pharmaceutics15092346/s1.

## References

[1] Flack J.M., Adekola B. (2020). Blood pressure and the new ACC/AHA hypertension guidelines. Trends Cardiovasc. Med..

[2] Ogihara T., Matsuzaki M., Matsuoka H., Shimamoto K., Shimada K., Rakugi H., Umemoto S., Kamiya A., Suzuki N., Kumagai H. (2005). The combination therapy of hypertension to prevent cardiovascular events (COPE) trial: Rationale and design. Hypertens. Res..

[3] Remiszewski P., Malinowska B. (2022). Why multitarget vasodilatory (endo)cannabinoids are not effective as antihypertensive compounds after chronic administration: Comparison of their effects on systemic and pulmonary hypertension. Pharmaceuticals.

[4] Arredondo A., Zúñiga A. (2006). Epidemiologic changes and economic burden of hypertension in Latin America: Evidence from Mexico. Am. J. Hypertens..

[5] Staffileno B.A. (2005). Treating hypertension with cardioprotective therapies: The role of ACE inhibitors, ARBs, and beta-blockers. J. Cardiovasc. Nurs..

[6] Monroy-Ortiz C., Castillo-España P. (2007). Plantas Medicinales Utilizadas en el Estado de Morelos.

[7] Palma-Tenango M., Sánchez-Fernández R.E., Soto-Hernández M. (2021). A Systematic Approach to *Agastache mexicana* Research: Biology, Agronomy, Phytochemistry, and Bioactivity. Molecules.

[8] Flores-Flores A., Hernández-Abreu O., Rios M.Y., León-Rivera I., Aguilar-Guadarrama B., Castillo-España P., Perea-Arango I., Estrada-Soto S. (2016). Vasorelaxant mode of action of dichloromethane-soluble extract from *Agastache mexicana* and its main bioactive compounds. Pharm. Biol..

[9] González-Trujano M.E., Ventura-Martínez R., Chávez M., Díaz-Reval I., Pellicer F. (2012). Spasmolytic and antinociceptive activities of ursolic acid and acacetin identified in *Agastache mexicana*. Planta Med..

[10] Hernández-Abreu O., Castillo-España P., León-Rivera I., Ibarra-Barajas M., Villalobos-Molina R., González-Christen J., Vergara-Galicia J., Estrada-Soto S. (2009). Antihypertensive and vasorelaxant effects of tilianin isolated from *Agastache mexicana* are mediated by NO/cGMP pathway and potassium channel opening. Biochem. Pharmacol..

[11] Wei J., Cao P., Wang J., Kang W. (2016). Analysis of Tilianin and Acacetin in *Agastache rugosa* by High-Performance Liquid Chromatography with Ionic Liquids-Ultrasound-Based Extraction. Chem. Cent. J..

[12] Hernández-Abreu O., Torres-Piedra M., García-Jiménez S., Ibarra-Barajas M., Villalobos-Molina R., Montes S., Rembao D., Estrada-Soto S. (2013). Dose-dependent antihypertensive determination and toxicological studies of tilianin isolated from *Agastache mexicana*. J. Ethnopharmacol..

[13] Schiozer A.L., Cabral E.C., de Godoy L.A., Chaves F., Poppi R.J., Riveros J.M., Pereira A.M.S., de Souza L.M., Barata L.E. (2012). Electrospray ionization mass spectrometry fingerprinting of extracts of the leaves of *Arrabidaea chica*. J. Braz. Chem. Soc..

[14] Draper J., Lloyd A.J., Goodacre R., Beckmann M. (2013). Flow infusion electrospray ionisation mass spectrometry for high throughput, non-targeted metabolite fingerprinting: A review. Metabolomics.

[15] Alcântara D.B., Riceli P., Almeida A.D.S., Luz L.R., Nascimento H.O., Fernandes T.S.M., Dionísio A.P., Castro A.C.R., Nascimento R.F., Lopes G.S. (2022). Development, optimization, and validation of an ultrasound-assisted liquid–liquid microextraction (UALLME) for selenomethionine analyses in cashew nut (*Anacardium occidentale*) by ultra-performance liquid chromatography coupled to electrospray ionization/single quadrupole mass spectrometer (UPLC-ESI/QDa). Food Anal. Methods.

[16] López J.L., Baltazar C., Torres M., Ruiz A., Esparza R., Rosas G., Pérez Campos R., Contreras Cuevas A., Esparza Muñoz R. (2017). Biosynthesis of silver nanoparticles using extracts of Mexican medicinal plants. Characterization of Metals and Alloys.

[17] El-Akhal J., Oliveira A.P., Bencheikh R., Valentão P., Andrade P.B., Morato M. (2022). Vasorelaxant Mechanism of Herbal Extracts from Mentha suaveolens, Conyza canadensis, Teucrium polium and Salvia verbenaca in the Aorta of Wistar Rats. Molecules.

[18] Yorsin S., Sriwiriyajan S., Chongsa W. (2022). Vasorelaxing Effect of Garcinia cowa Leaf Extract in Rat Thoracic Aorta and Its Underlying Mechanisms. J. Tradit. Complement. Med..

[19] Jin S.N., Wen J.F., Li X., Kang D.G., Lee H.S., Cho K.W. (2011). The Mechanism of Vasorelaxation Induced by Ethanol Extract of *Sophora flavescens* in Rat Aorta. J. Ethnopharmacol..

[20] Kim B., Kwon Y., Lee S., Lee K., Ham I., Choi H.Y. (2017). Vasorelaxant Effects of Angelica decursiva Root on Isolated Rat Aortic Rings. BMC Complement. Altern. Med..

[21] Javed A., Khan S., Salma U., Ahmad T., Khan T., Shah A.J. (2023). Extract of Chenopodium album lowers blood pressure in rats through endothelium-dependent and -independent vasorelaxation. Ann. Pharm. Fr..

[22] Organization for Economic Co-Operation and Development (OECD) (2002). Test No. 423: Acute oral toxicity-Acute Toxic Class Method. OECD Guidelines for the Testing of Chemicals.

[23] Mbosso Teinkela J.E., Oumarou H., Siwe Noundou X., Meyer F., Megalizzi V., Hoppe H.C., Macedo Krause R.W., Wintjens R. (2023). Evaluation of in vitro antiplasmodial, antiproliferative activities, and in vivo oral acute toxicity of Spathodea campanulata flowers. Sci. Afr..

[24] Estrada-Soto S., Ornelas-Mendoza K., Navarrete-Vázquez G., Chávez-Silva F., Almanza-Pérez J.C., Villalobos-Molina R., Ortiz-Barragán E., Loza-Rodríguez H., Rivera-Leyva J.C., Flores-Flores A. (2023). Insulin sensitization by PPARγ and GLUT-4 overexpression/translocation mediates the antidiabetic effect of *Plantago australis*. Pharmaceuticals.

[25] Sharma H., Kumar P., Deshmukh R.R., Bishayee A., Kumar S. (2018). Pentacyclic Triterpenes: New Tools to Fight Metabolic Syndrome. Phytomedicine.

[26] Muruganathan N., Dhanapal A.R., Baskar V., Muthuramalingam P., Selvaraj D., Aara H., Shiek Abdullah M.Z., Sivanesan I. (2022). Recent Updates on Source, Biosynthesis, and Therapeutic Potential of Natural Flavonoid Luteolin: A Review. Metabolites.

[27] Bangar S.P., Kajla P., Chaudhary V., Sharma N., Ozogul F. (2023). Luteolin: A flavone with myriads of bioactivities and food applications. Food Biosci..

[28] Nguyen H.N., Ullevig S.L., Short J.D., Wang L., Ahn Y.J., Asmis R. (2021). Ursolic Acid and Related Analogues: Triterpenoids with Broad Health Benefits. Antioxidants.

[29] Woźniak Ł., Skąpska S., Marszałek K. (2015). Ursolic Acid—A Pentacyclic Triterpenoid with a Wide Spectrum of Pharmacological Activities. Molecules.

