# Supplementary material for: LC-MS Fingerprinting Development for Standardized Precipitate from Agastache mexicana, Which Induces Antihypertensive Effect through NO Production and Calcium Channel Blockade
Source: Pharmaceutics. 2023 Sep 19;15(9):2346. doi: 10.3390/pharmaceutics15092346 (PMC10538051; doi:10.3390/pharmaceutics15092346)
Supplement: Supplementary file 1 [file pharmaceutics-15-02346-s001.zip › pharmaceutics-2487550-SM.pdf]

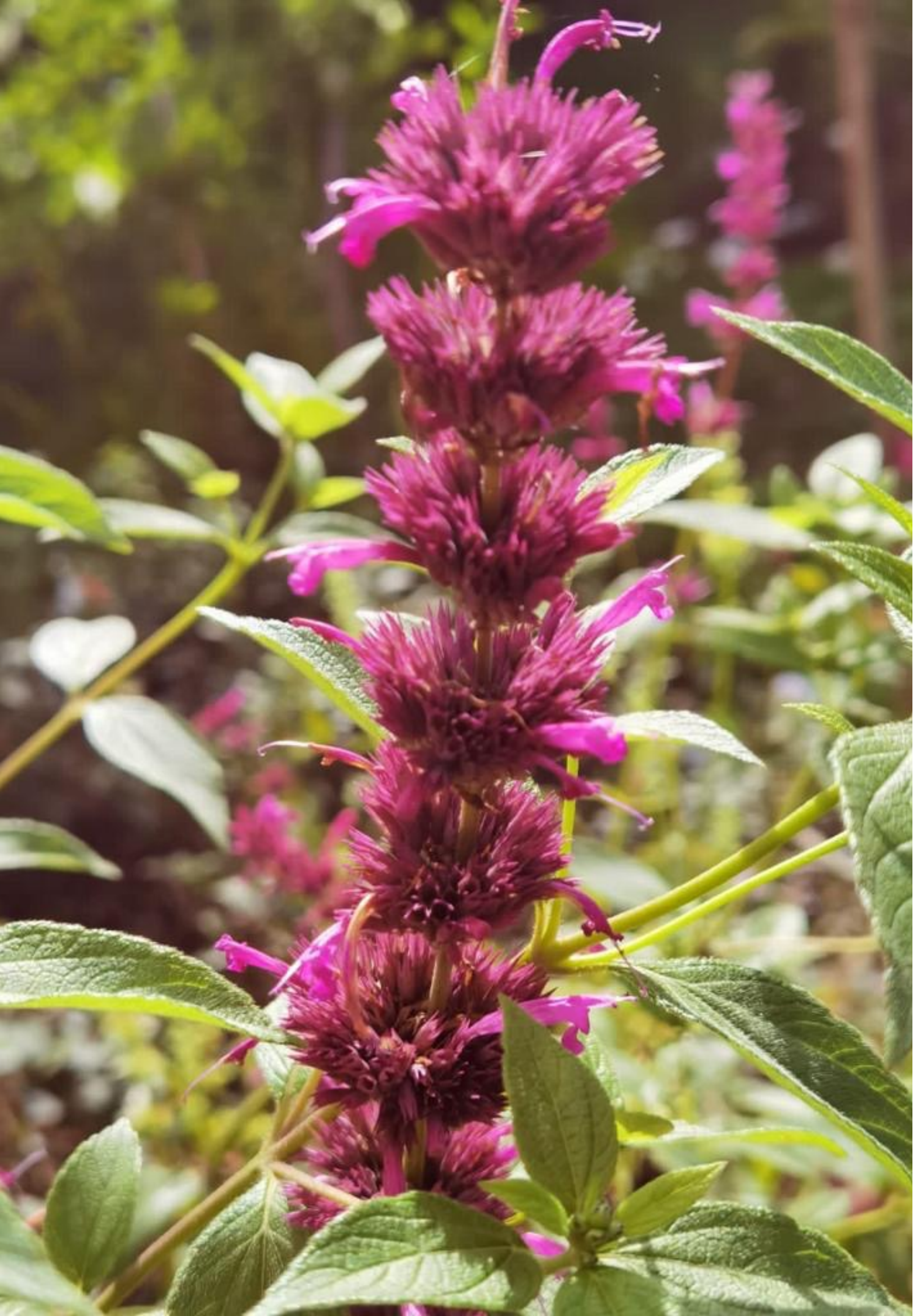

Figure S1: Fresh collected *Agastache Mexicana* aerial parts original photographs taken by authors.

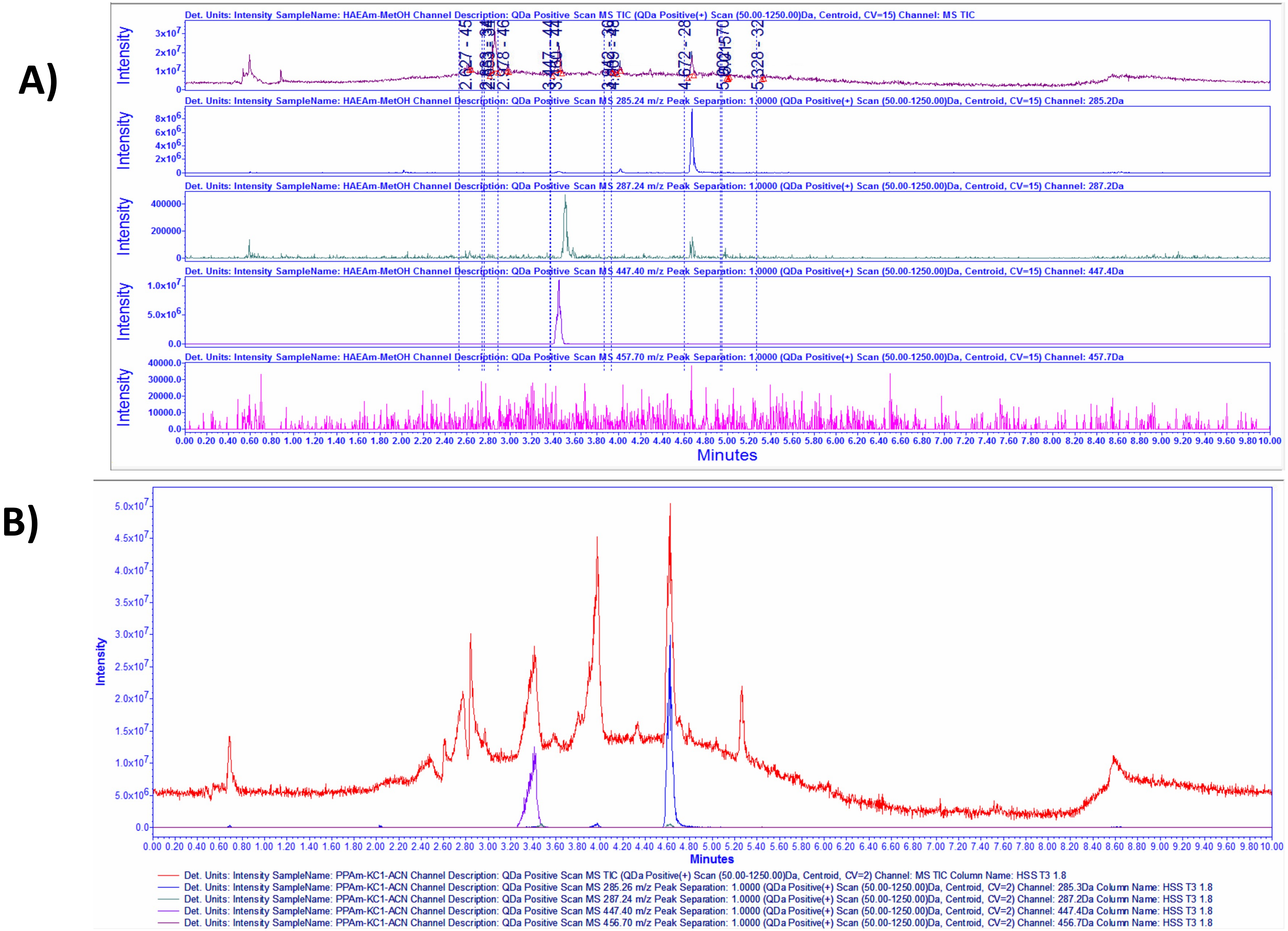

Figure S2: Comparison of retention times and detected masses gradient elution, ionization mode ESI +, for HAEAm A) sample in methanol B) Sample in acetonitrile, performed in column ACQUITY UPLC® HSS T3 130 Å (1.8 μm, 2.1 × 100 mm).

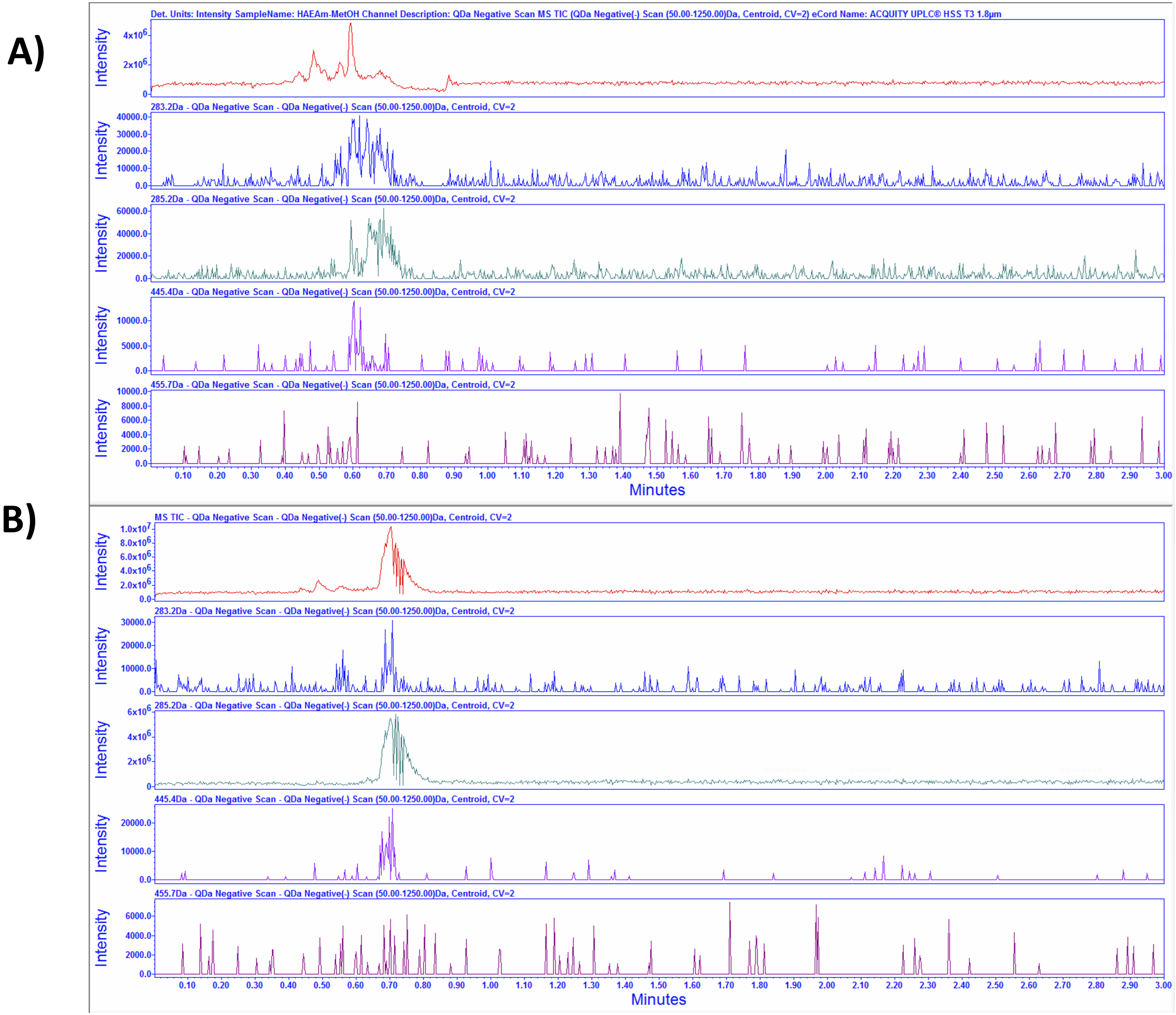

Figure S3: Comparison of retention times and detected masses isocratic elution, ionization mode ESI +, for HAEAm **A)** sample in methanol **B)** Sample in acetonitrile, performed in column ACQUITY UPLC® HSS T3 130 Å (1.8 µm, 2.1 × 100 mm).

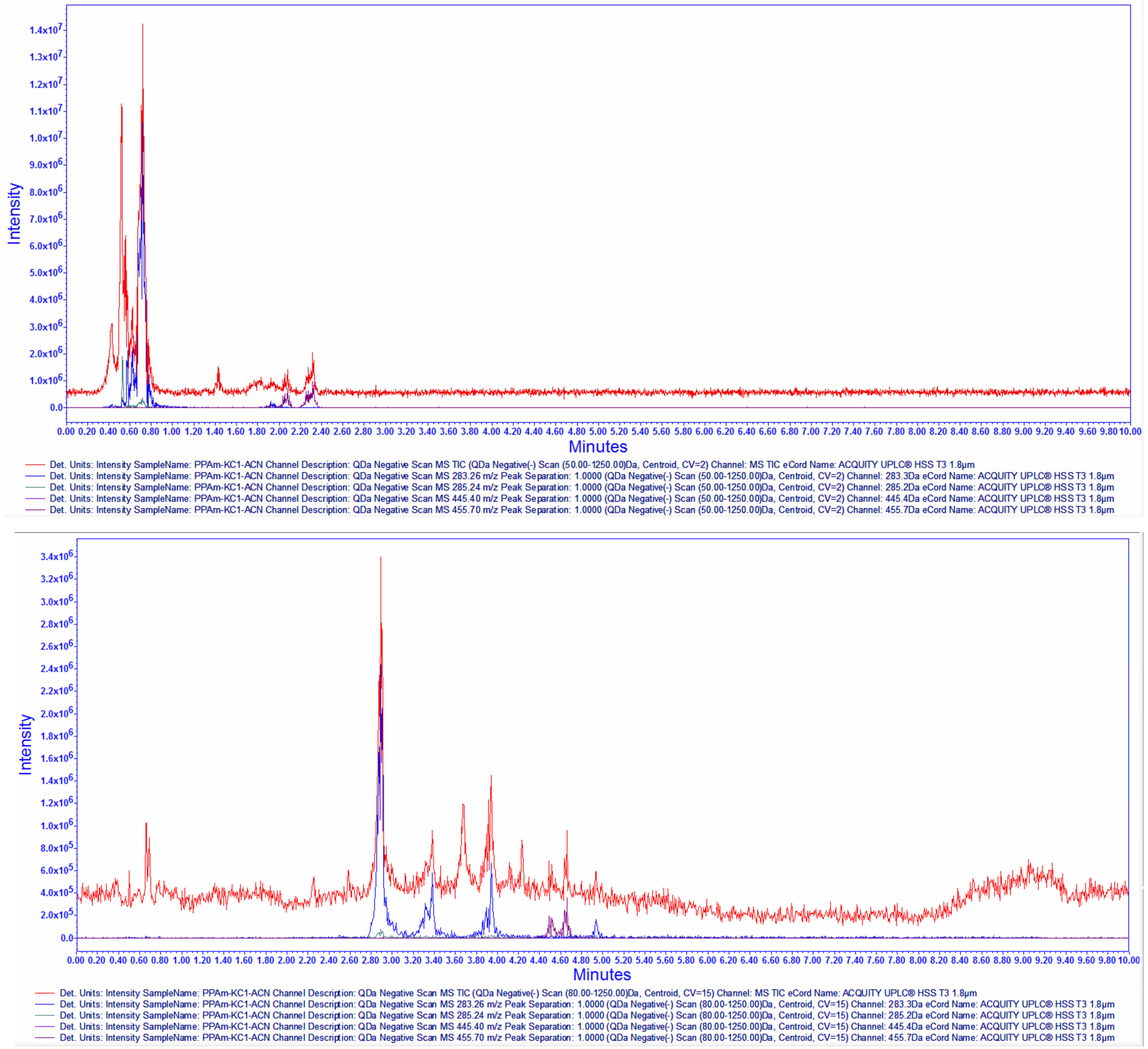

Mass channel selection/SIR ESI-		Compound name targeted	Retention time isocratic elution		Retention time gradient elution	
			PP*Am*	Standard	PP*Am*	Standard
1	283.26 Da	Acacetin	0.718	0.93	2.87	2.83
2	285.24 Da	Luteolin	0.521	0.583	2.81	2.81
3	445.40 Da	Tilianin	ND	ND	ND	ND
4	455.7 Da	Oleanolic acid	2.27	2.12	4.5	4.47
5	455.7 Da	Ursolic acid	2.35	2.39	4.65	4.65

Figure S4: Comparison of retention times and detected masses in isocratic and gradient elution, ionization mode for PP*Am* ESI- performed in column ACQUITY UPLC® HSS T3 130 Å (1.8 µm, 2.1 × 100 mm).

Table S1: Comparison of retention times and detected masses in isocratic and gradient elution, ionization mode ESI +, performed in column ACQUITY UPLC® HSS T3 130 Å (1.8 µm, 2.1 × 100 mm) for standards, and samples HAE*Am* and PP*Am*, ND= Not determined.

**Mass channel selection in Daltons (Da) / SIR ESI+**	**Acacetin** 285.26	**Luteolin** 287.24	**Tilianin** 447.40	**Oleanolic acid** 457.7	**Ursolic acid** 457.7
**RETENTION TIME IN MINUTES**					
HAE*Am* in acetonitrile	4.669	3.49	3.438	ND	ND
HAE*Am* in methanol	4.564	3.432	3.374	ND	ND
PP*Am* in acetonitrile	4.610	3.470	3.35	ND	ND
PP*Am* in methanol	4.589	3.443	3.418	ND	ND
Standards in acetronitrile	4.624	3.373	3.36	ND	ND
Standards in methanol	4.548	3.433	3.389	ND	ND

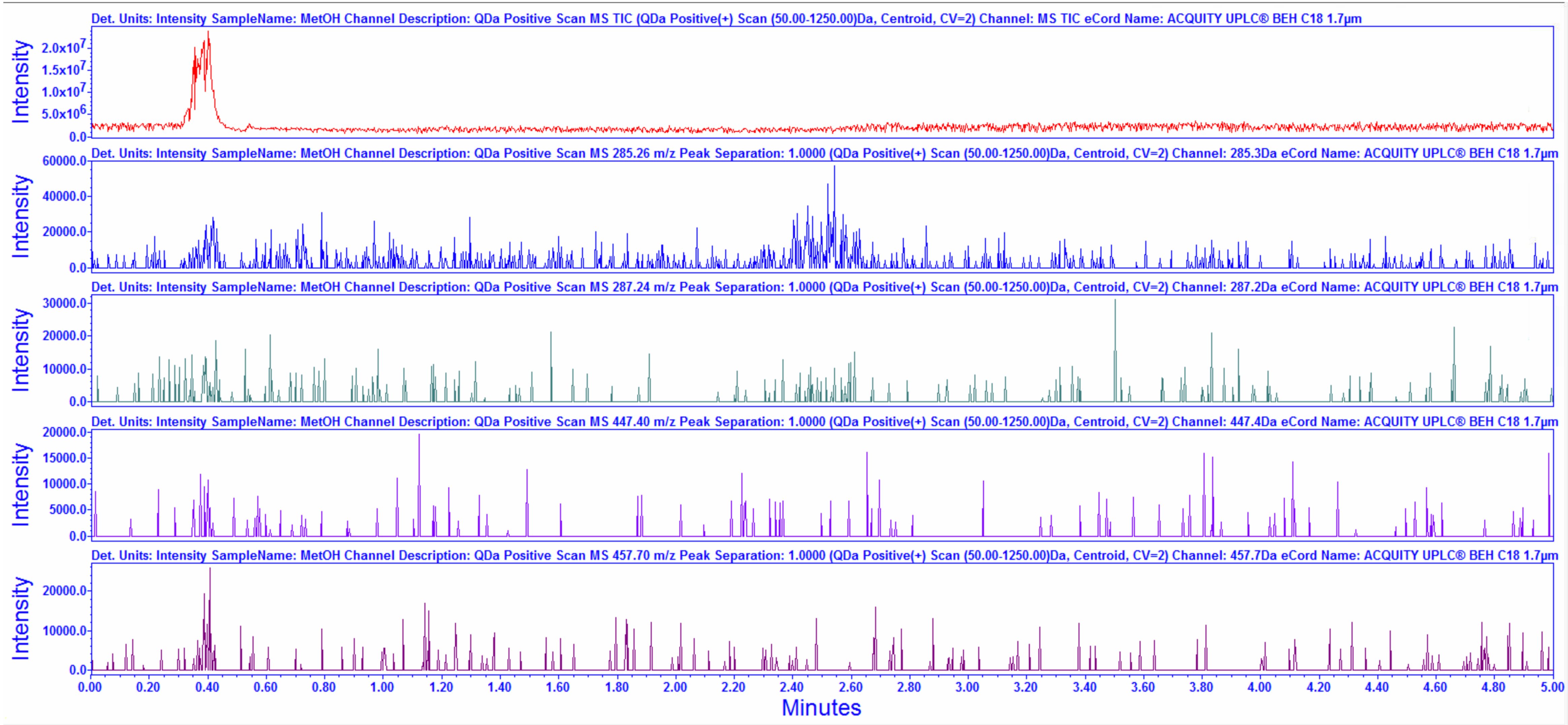

Figure S5: Stack plot from positive mode scan for blank methanol **A)** total ion chromatogram, exploratory mass scan from 50-1250, Selected channels Da m/Z [ESI+] **B)**Acacetin 285.26**C)** Luteolin 287.2 **D)** Tilianin 445.4 **E)** **Oleanoic and Ursolic acid** 455.7 [the X-axis represents time in minutes, and Y-axis represents signal intensity]. Column ACQUITY UPLC® HBE C18 130 Å column (1.7 µm, 2.1 x 50mm).

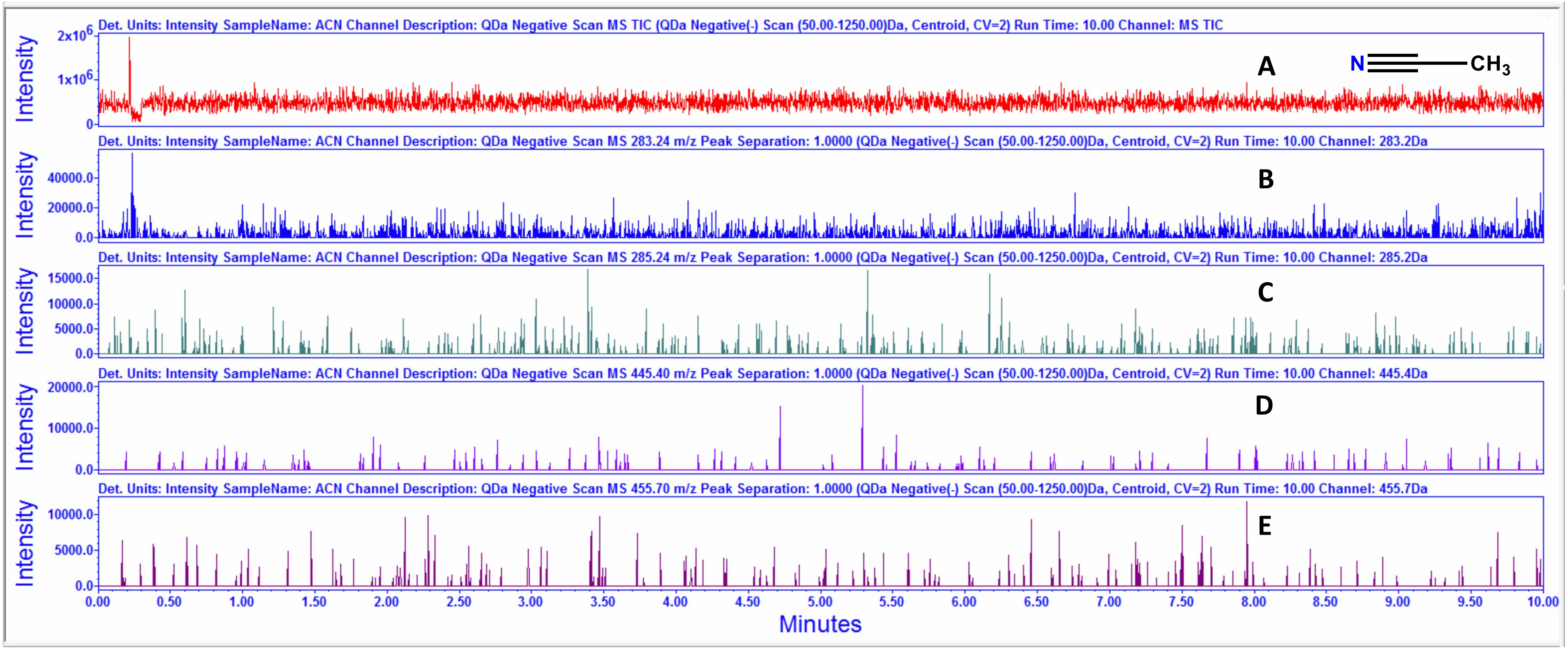

Figure S6: Stack plot from negative mode scan for blank acetonitrile **A)** total ion chromatogram, exploratory mass scan from 50-1250 Da **B)**Channel for 283.26Da mass **C)** Channel for 285.2 Da mass **D)** Channel for 445.4 Da mass **E)** Channel for 455.7 Da mass [the X-axis represents time in minutes, and Y-axis represents signal intensity]. Column ACQUITY UPLC® HBE C18 130 Å column (1.7 μm, 2.1 x 50mm).

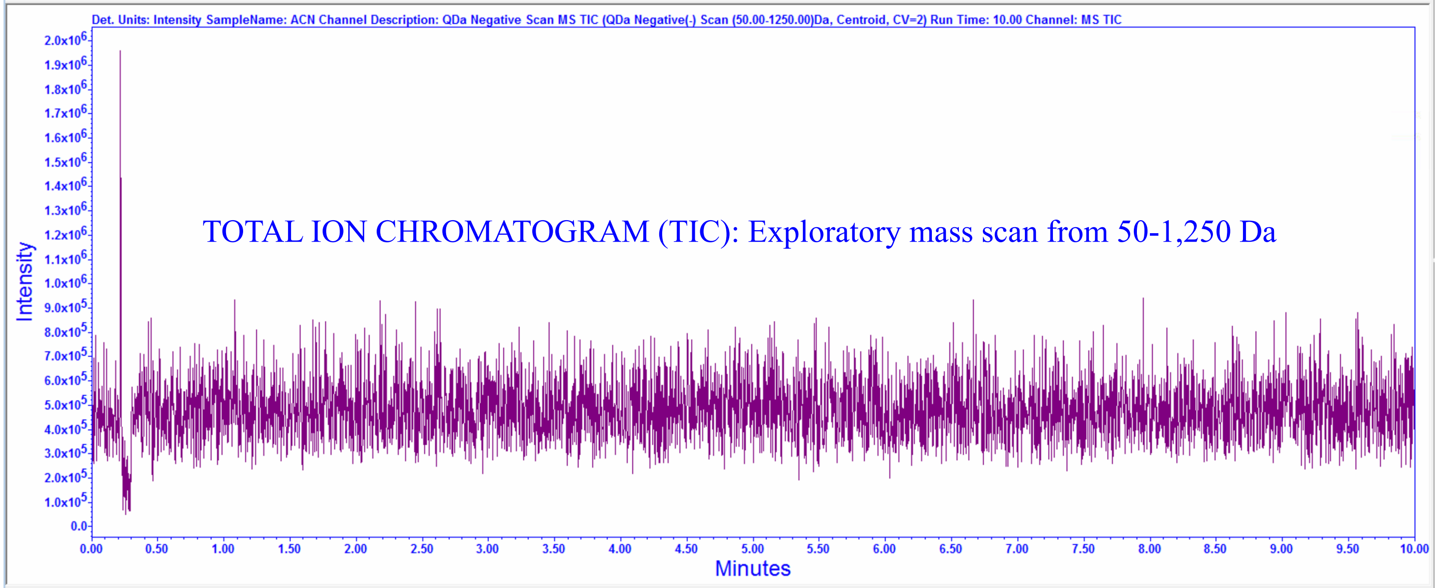

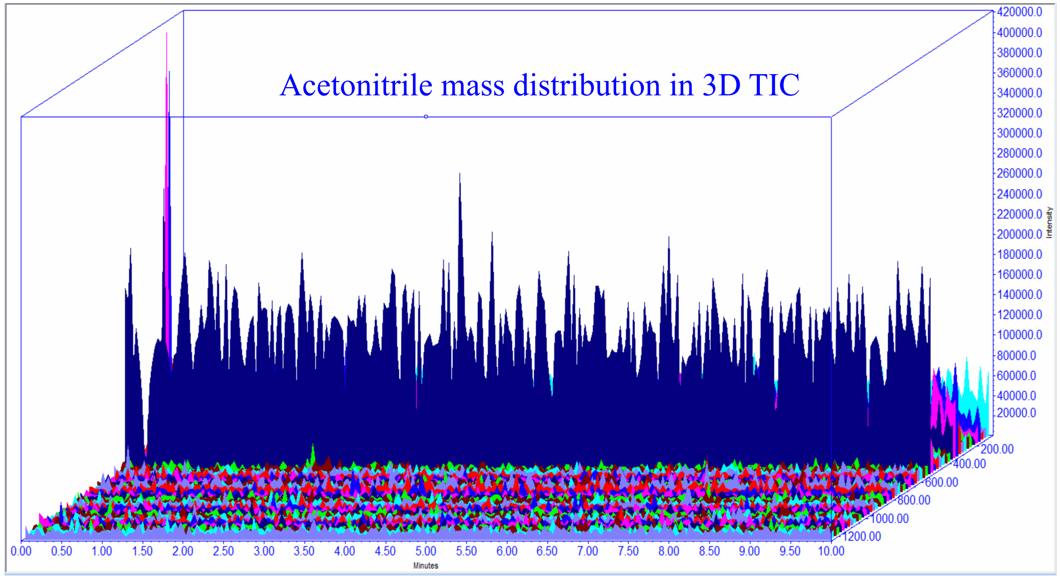

SIR experiment, 4 channels selected for Acetonitrile, negative mode (ESI-)

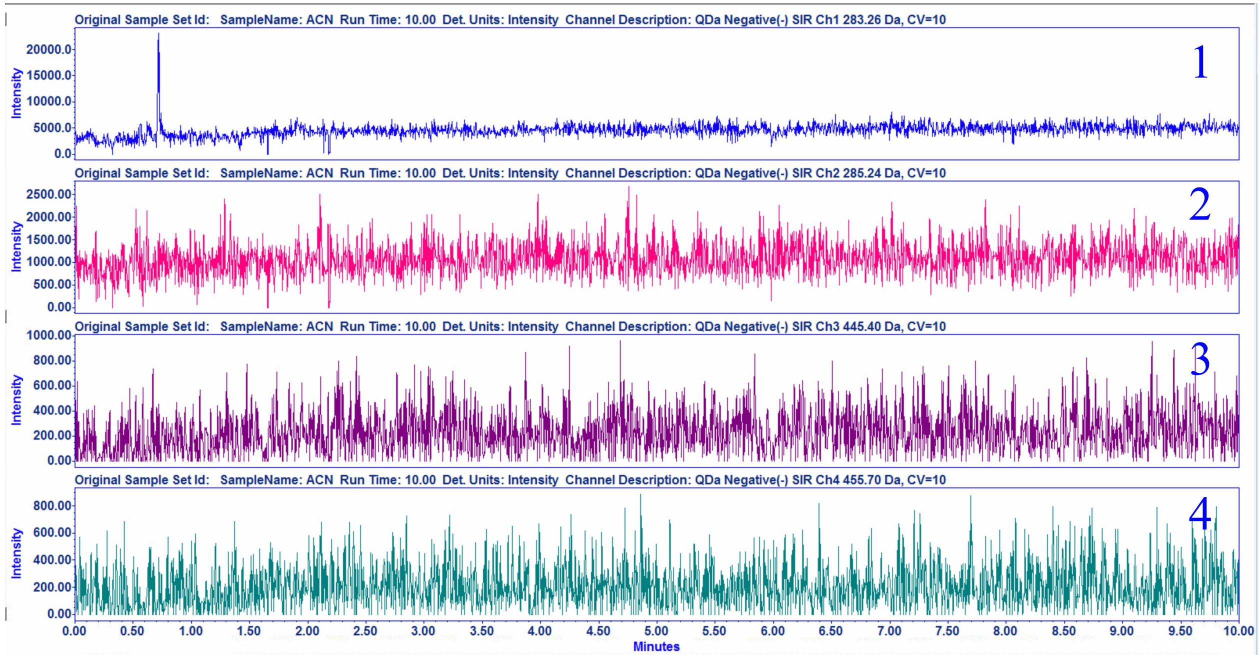

Table of the compound's names for SIR experiment

	Mass channel selection/SIR	Compound name targeted
1	QDa Ch1 283.26 Da	Acacetin
2	QDa Ch2 285.26 Da	Luteolin
3	QDa Ch3 445.40 Da	Tilianin
4	QDa Ch9 455.70 Da	Ursolic acid

Figure S7: Chromatographic experiments for acetonitrile. Column ACQUITY UPLC® HBE C18 130 Å column (1.7 µm, 2.1 x 50mm).

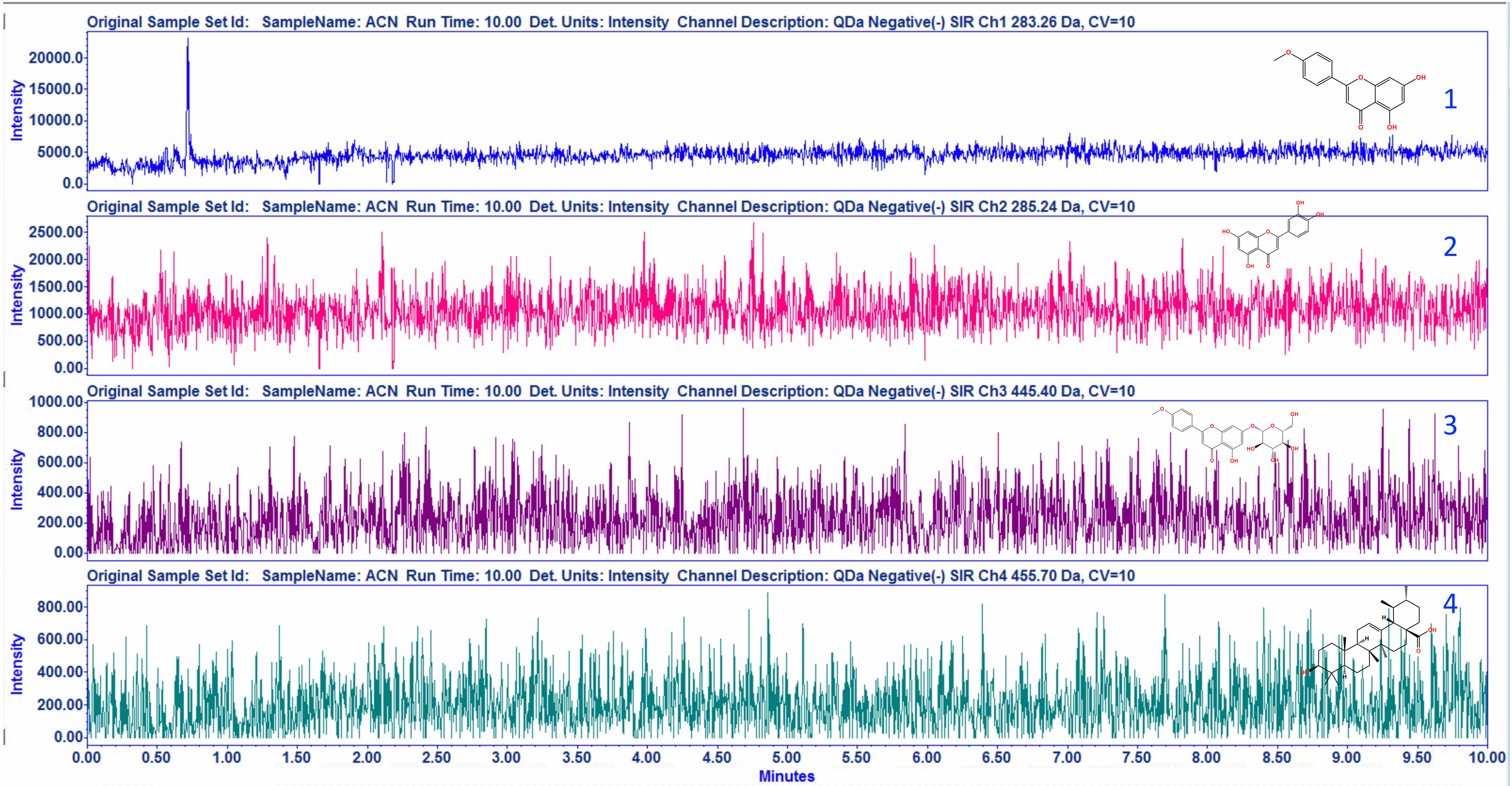

Figure S8: SIR experiment, 3 channels selected for ursolic acid standard negative mode (ESI-) 1) Acacetin 283.26 Da 2)Luteolin 28.24 Da 3) Tilianin 445.4 Da 4) Ursolic acid 455.41 Da [the X-axis represents time in minutes, and Y-axis represents signal intensity]. Column ACQUITY UPLC® HBE C18 130 Å column (1.7 µm, 2.1 x 50mm).

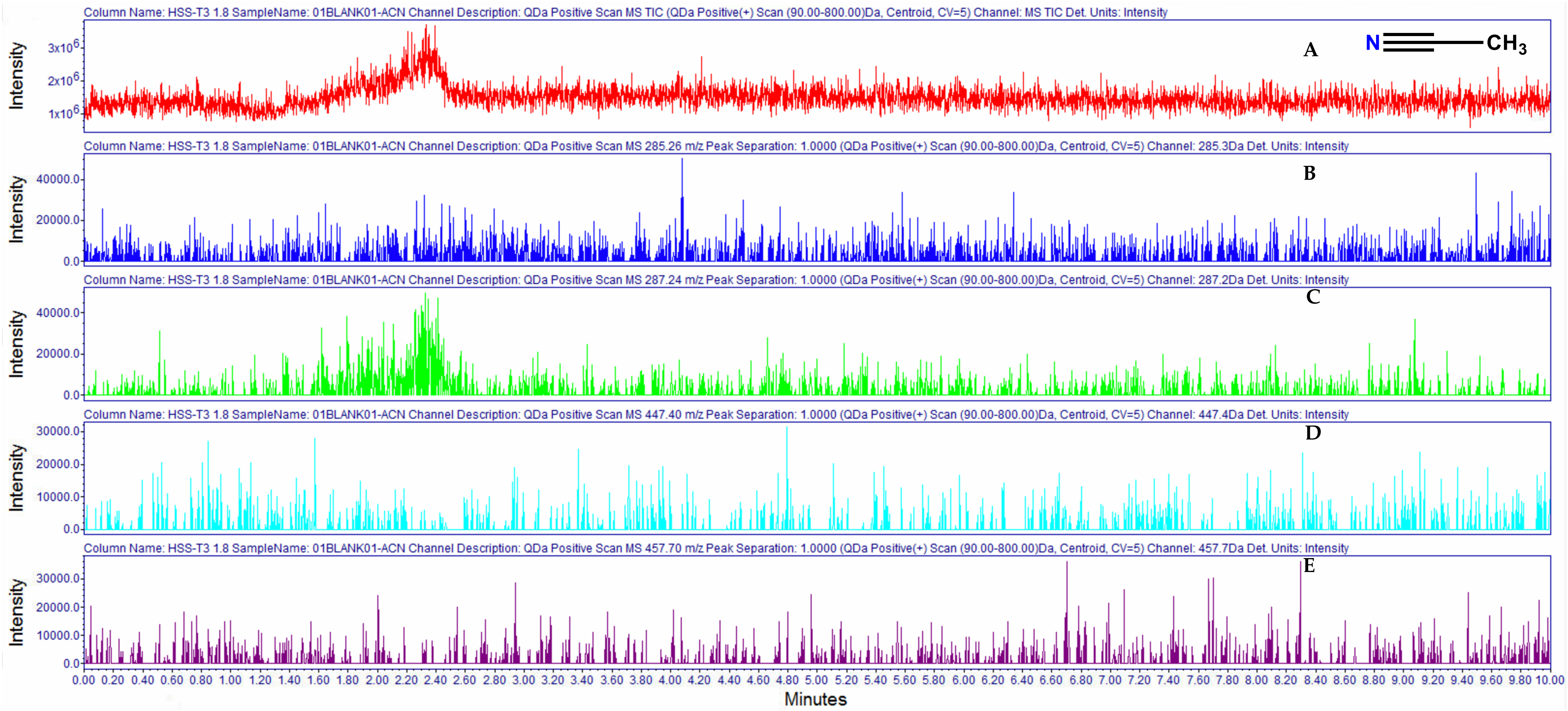

Figure S9: Stack plot from Mass Scan in positive mode (ESI+) for blank acetonitrile A) Total ion chromatogram, exploratory mass scan from 90-800 Da Selected channels for B) Acacetin 285.26 Da m/z [M+1] C) Luteolin 287.2 Da m/z [M+1] D) Tilianin 447.4 Da m/z [M+1] E) Oleanolic and ursolic acid 457.7 Da m/z [M+1]. The X-axis represents time in minutes, and Y-axis represents signal intensity; chromatographic column ACQUITY UPLC® HSS T3 130 Å (1.8 μm, 2.1 × 100 mm).

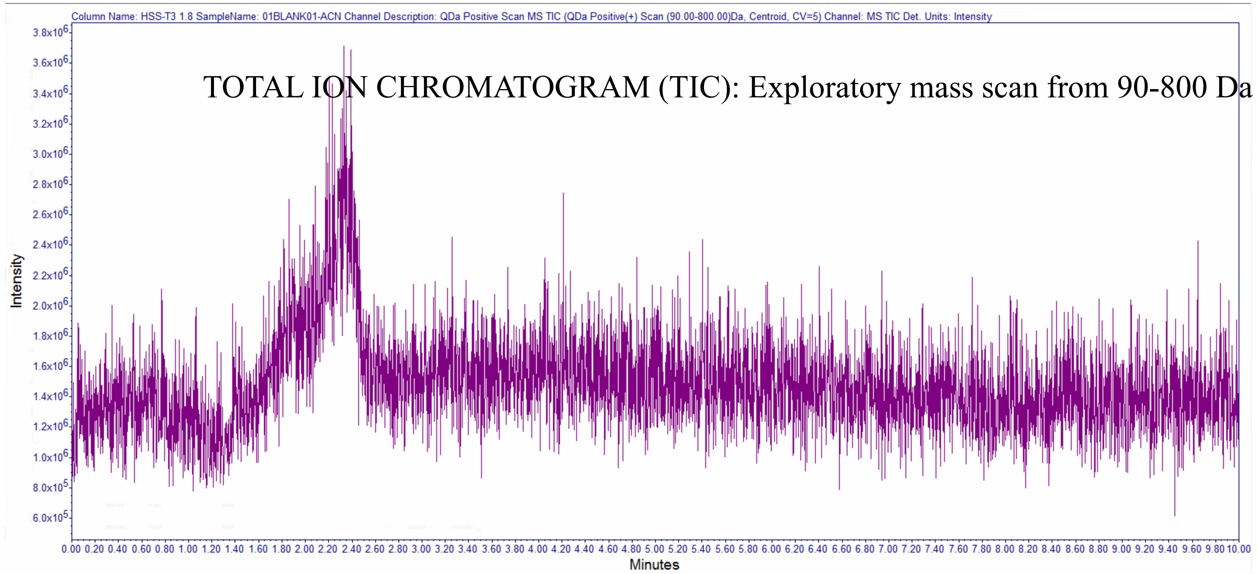

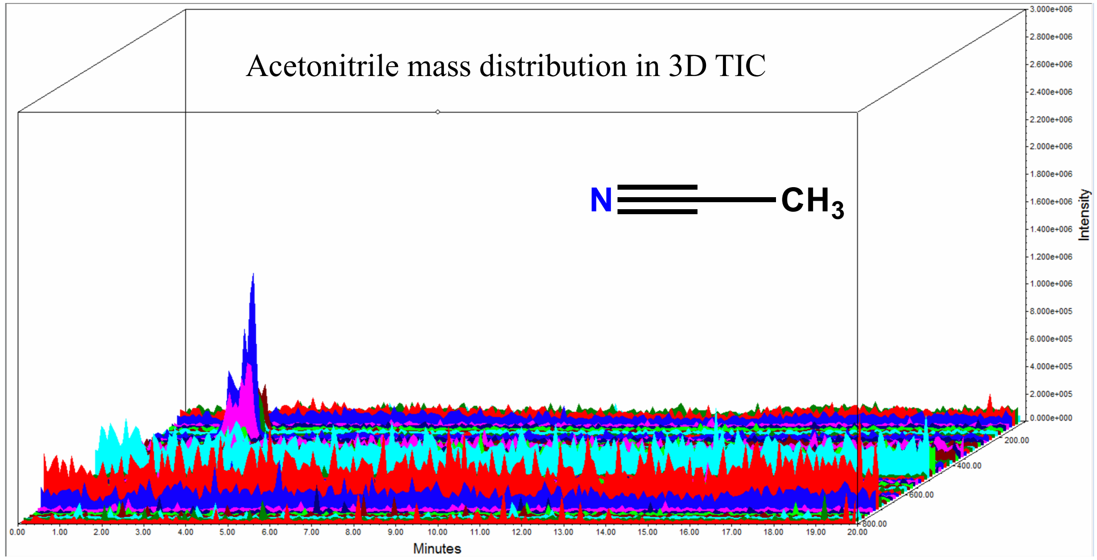

SIR experiment, 4 channels selected for Acetonitrile, positive mode (ESI+)

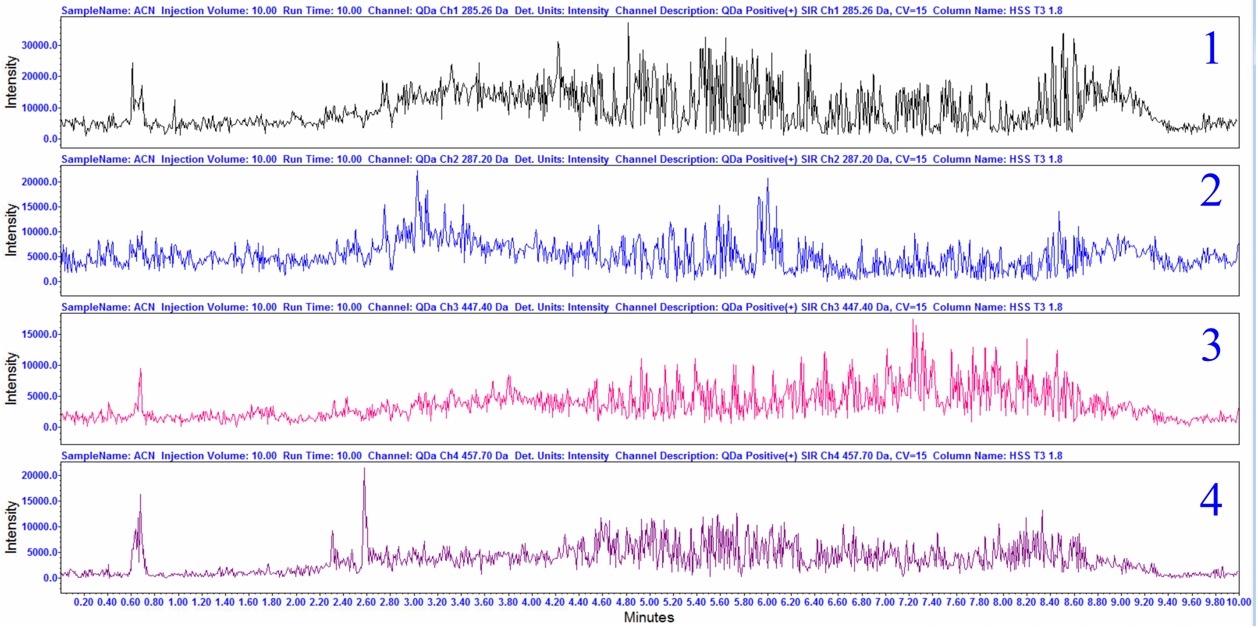

Table of the compound's names for SIR experiment

	**Mass channel selection/SIR (ESI+)** m/z Da	**Compound name targeted**
1	QDa Ch1 285.26	Acacetin
2	QDa Ch2 287.26	Luteolin
3	QDa Ch3 447.40	Tilianin
4	QDa Ch9 457.70	Ursolic acid /Oleanolic acid

Figure S10: Chromatographic experiments for blank of acetonitrile in positive mode (ESI+) chromatographic column ACQUITY UPLC® HSS T3 130 Å (1.8 µm, 2.1 × 100 mm).

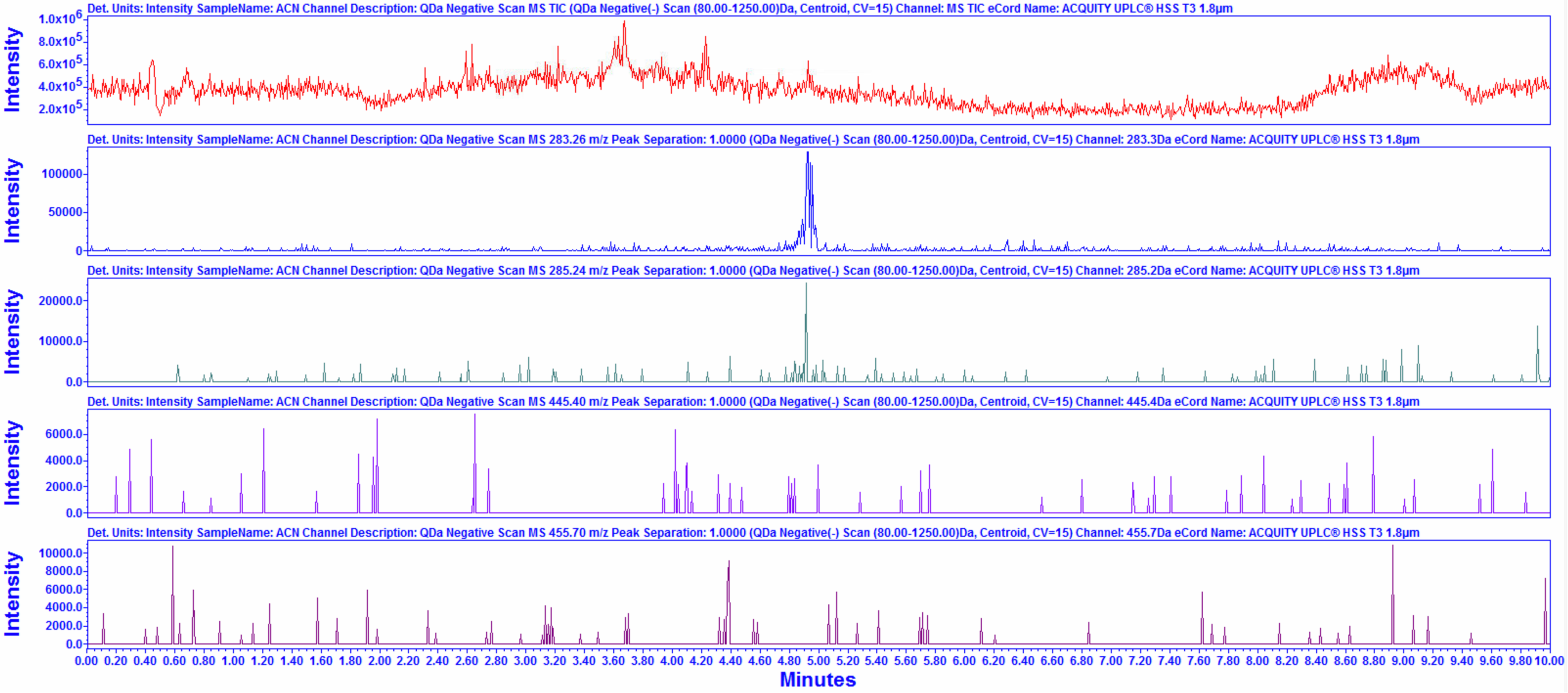

Figure S11: Stack plot from Mass Scan in negative mode (ESI-) for blank acetonitrile A) Total ion chromatogram, exploratory mass scan from 80-1250 Da Selected channels for B) Acacetin 283.26 Da m/z [M-1] C) Luteolin 285.2 Da m/z [M-1] D) Tilianin 445.4 Da m/z [M-1] E) Oleanolic and ursolic acid 455.7 Da m/z [M-1]. The X-axis represents time in minutes, and Y-axis represents signal intensity; chromatographic column ACQUITY UPLC® HSS T3 130 Å (1.8 µm, 2.1 × 100 mm).

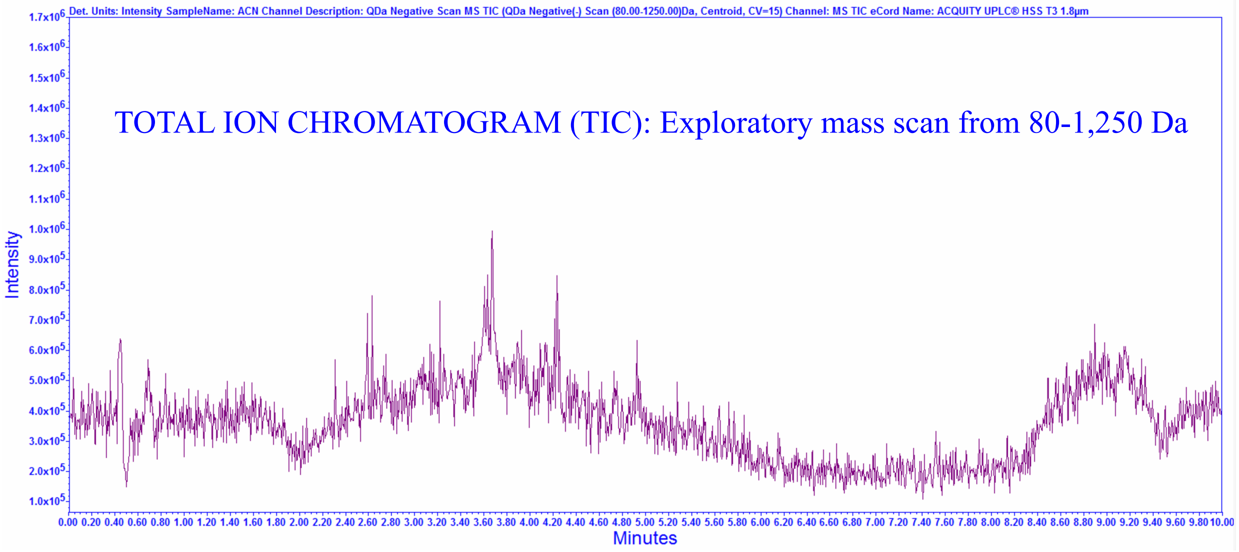

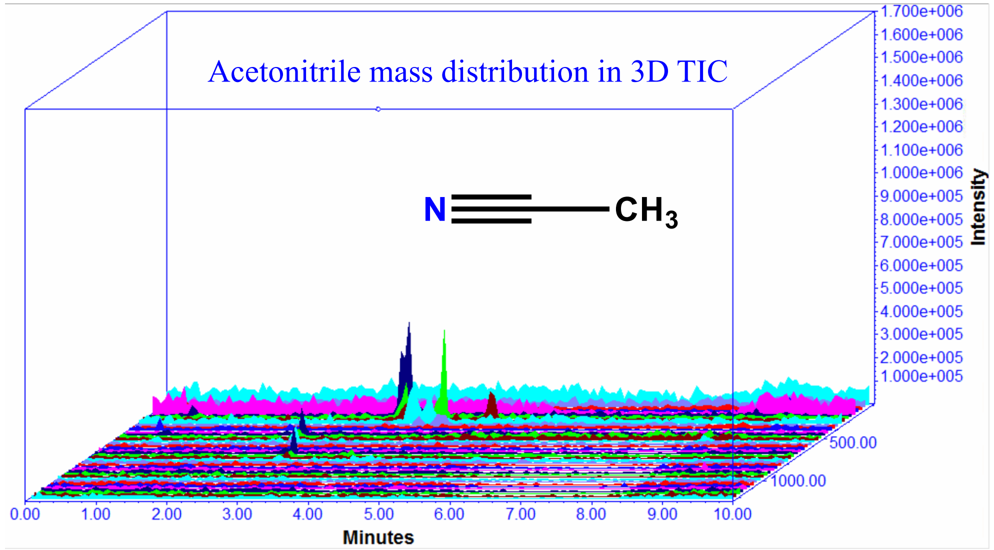

SIR experiment, 4 channels selected for Acetonitrile, negative mode (ESI-)

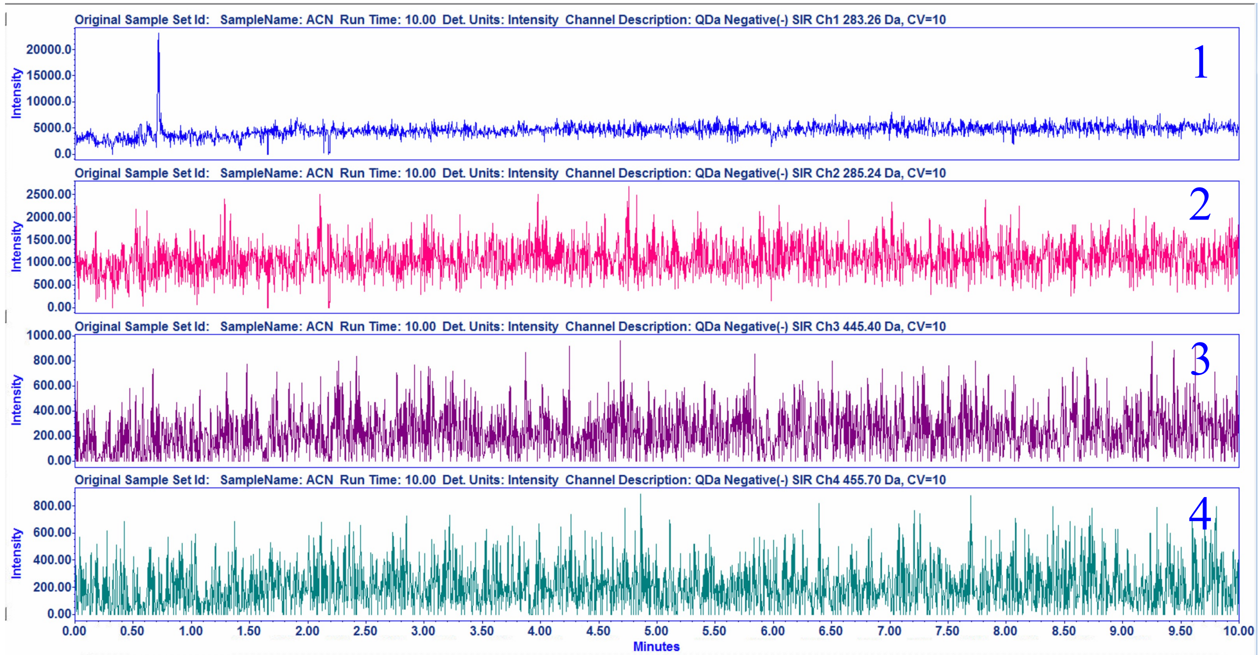

Table of the compound's names for SIR experiment

	**Mass channel selection/SIR (ESI-)** m/z Da	**Compound name targeted**
1	QDa Ch1 283.26 Da	Acacetin
2	QDa Ch2 285.26 Da	Luteolin
3	QDa Ch3 445.40 Da	Tilianin
4	QDa Ch9 455.70 Da	Ursolic acid /Oleanolic acid

Figure S12: Chromatographic experiments for blank of acetonitrile chromatographic column ACQUITY UPLC® HSS T3 130 Å (1.8 µm, 2.1 × 100 mm).

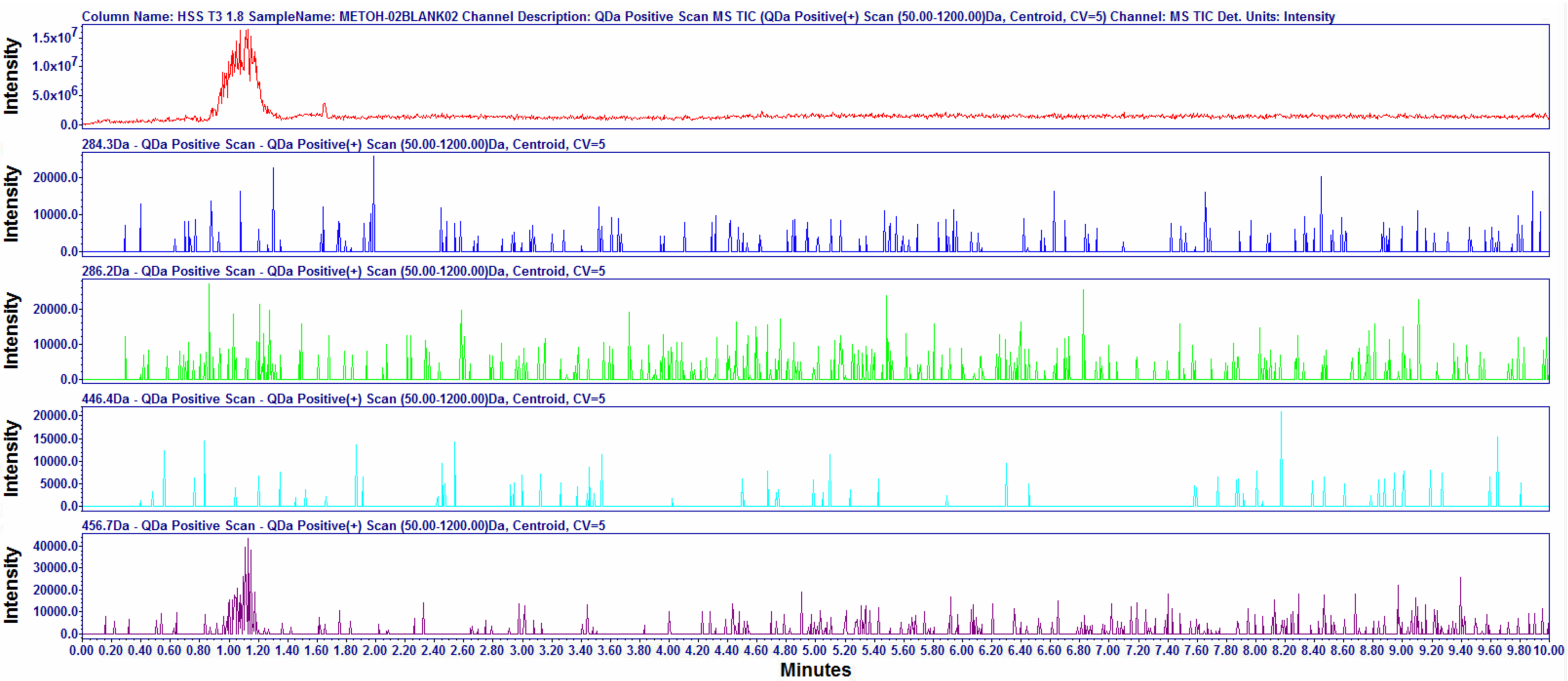

Figure S13: Stack plot from positive mode (ESI+) scan for blank acetonitrile A) total ion chromatogram, exploratory mass scan from 90-800 Da B)Channel for acacetin 285.26 Da mass C) Channel for luteolin 287.2 Da mass D) Channel for tilianin 447.4 Da mass E) Channel for oleanolic and ursolic acid 457.7 Da mass [the X-axis represents time in minutes, and Y-axis represents signal intensity] chromatographic column ACQUITY UPLC® HSS T3 130 Å (1.8 µm, 2.1 × 100 mm).

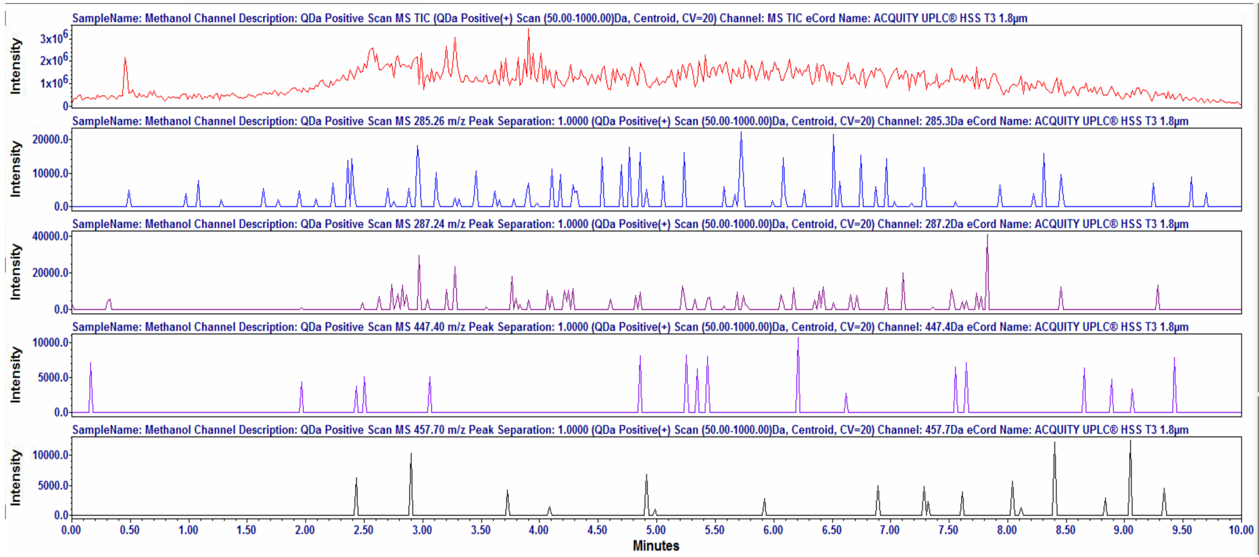

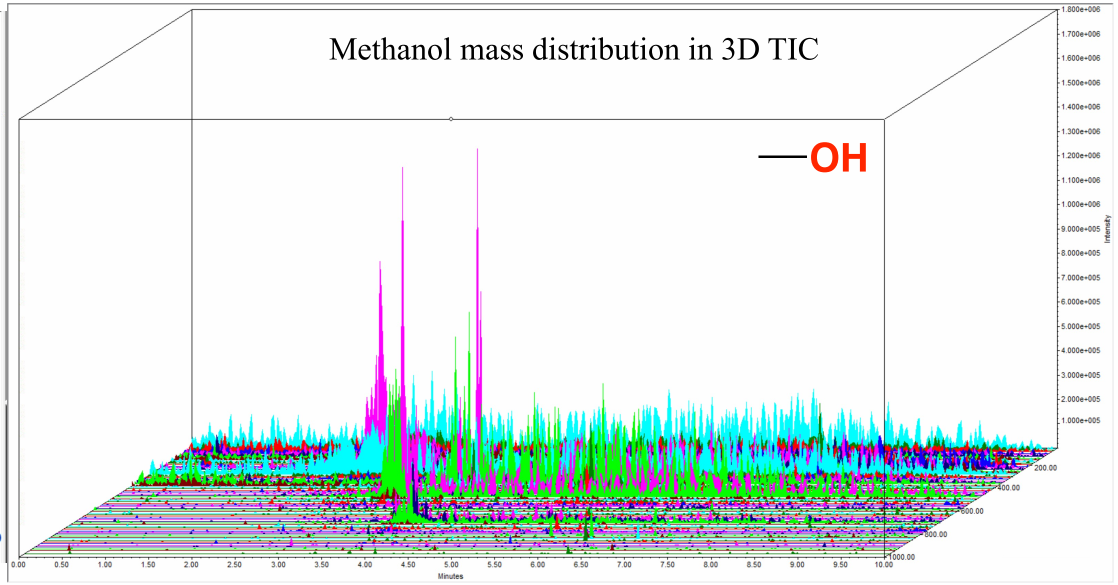

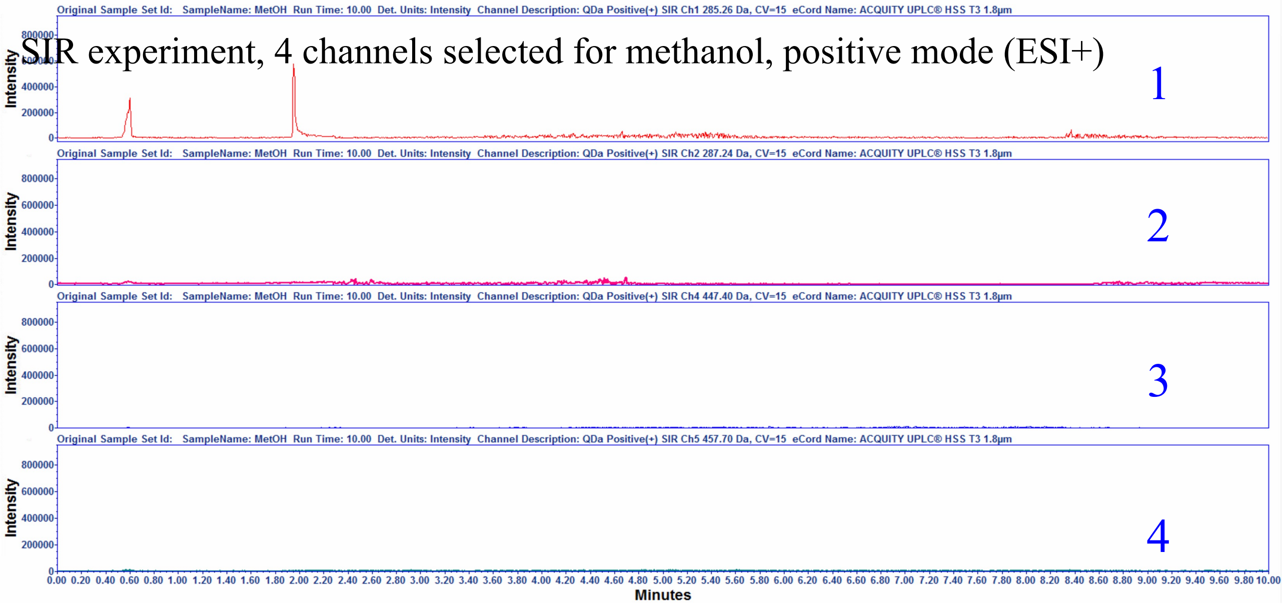

Table of the compound's names for SIR experiment

	Mass channel selection/SIR (ESI+) m/z Da	Compound name targeted
1	QDa Ch1 285.26	Acacetin
2	QDa Ch2 287.26	Luteolin
3	QDa Ch3 447.40	Tilianin
4	QDa Ch9 457.70	Ursolic acid /Oleanolic acid

Figure S14: Chromatographic experiments for blank of methanol in column ACQUITY UPLC® HSS T3 130 Å (1.8 µm, 2.1 × 100 mm).

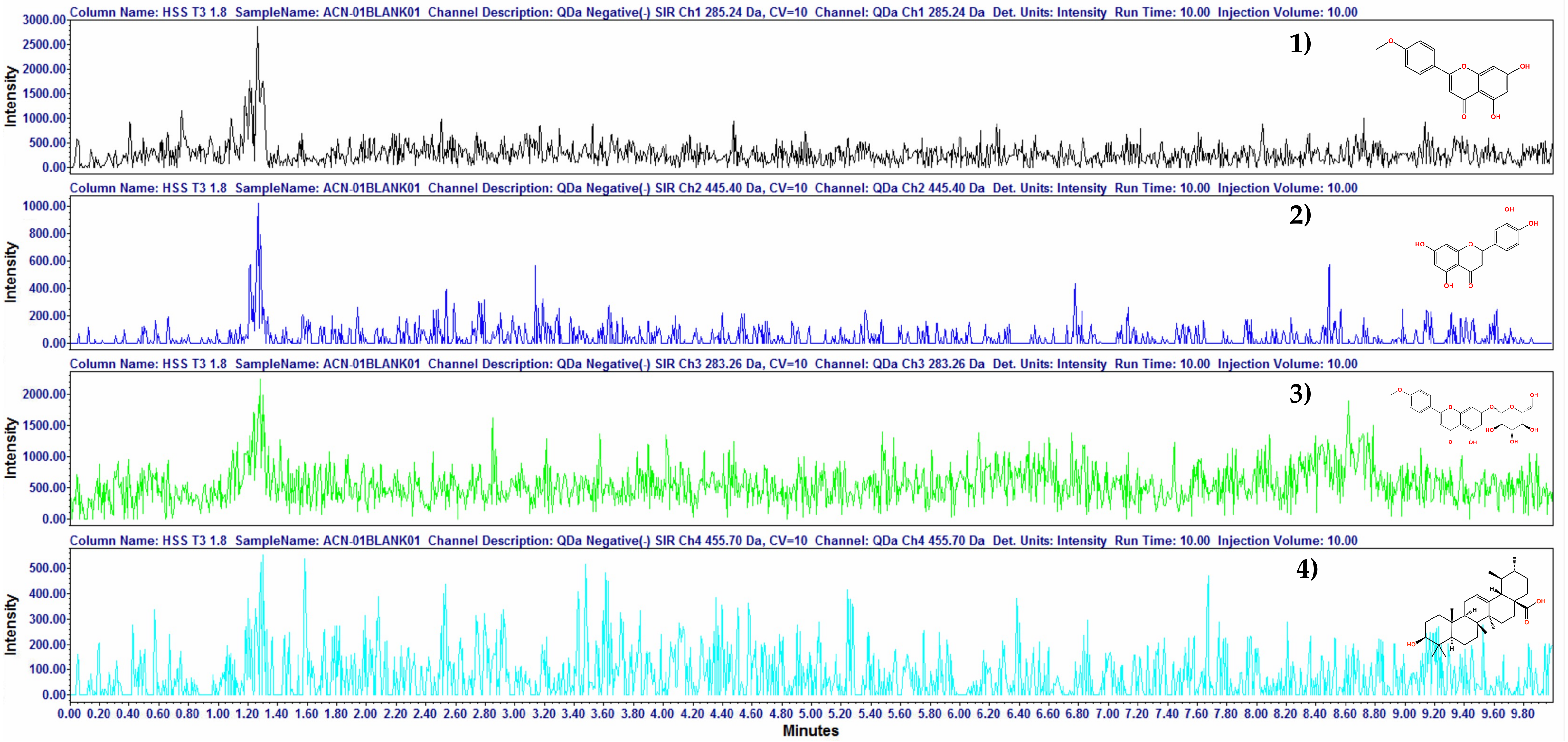

Figure S15: SIR experiment, 4 channels selected for blank acetonitrile negative mode (ESI-) 1) Acacetin 283.26 Da 2)Luteolin 28.24 Da 3) Tilianin 445.4 Da 4) Ursolic acid / Oleanolic acid 455.41 Da [the X-axis represents time in minutes, and Y-axis represents signal intensity] chromatographic column ACQUITY UPLC® HSS T3 130 Å (1.8 µm, 2.1 × 100 mm).

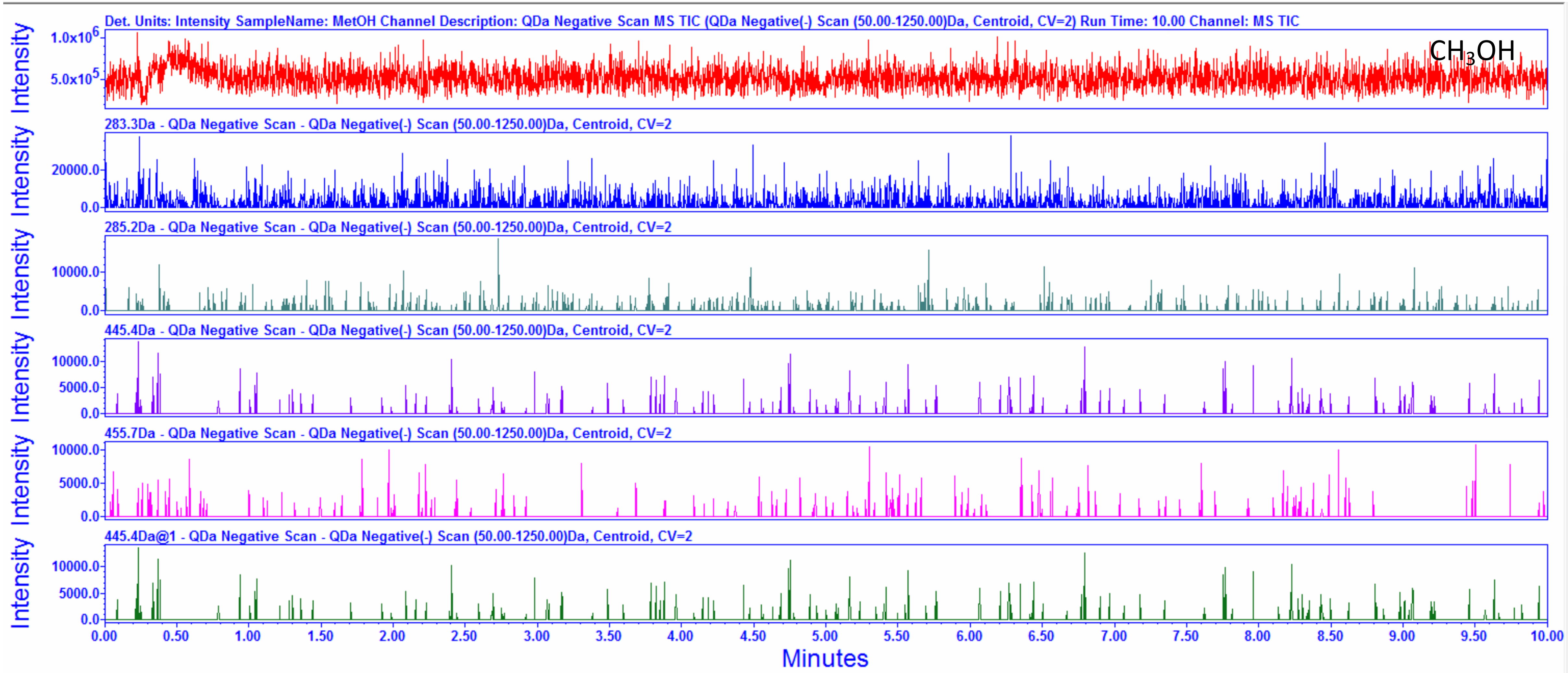

Figure S16: Stack plot from for blank methanol, negative mode scan ESI- **A)** total ion chromatogram, exploratory mass scan from 50-1250 Da **B)**Channel for 283.26Da mass **C)** Channel for 285.2 Da mass **D)** Channel for 445.4 Da mass **E)** Channel for 455.7 Da mass [the X-axis represents time in minutes, and Y-axis represents signal intensity].

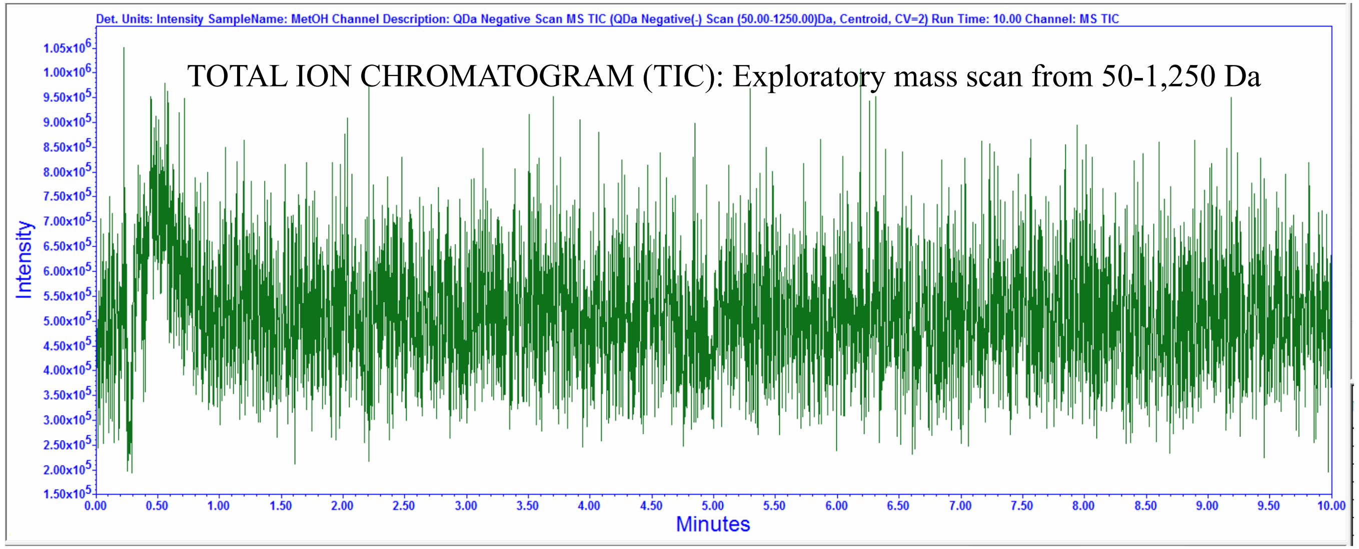

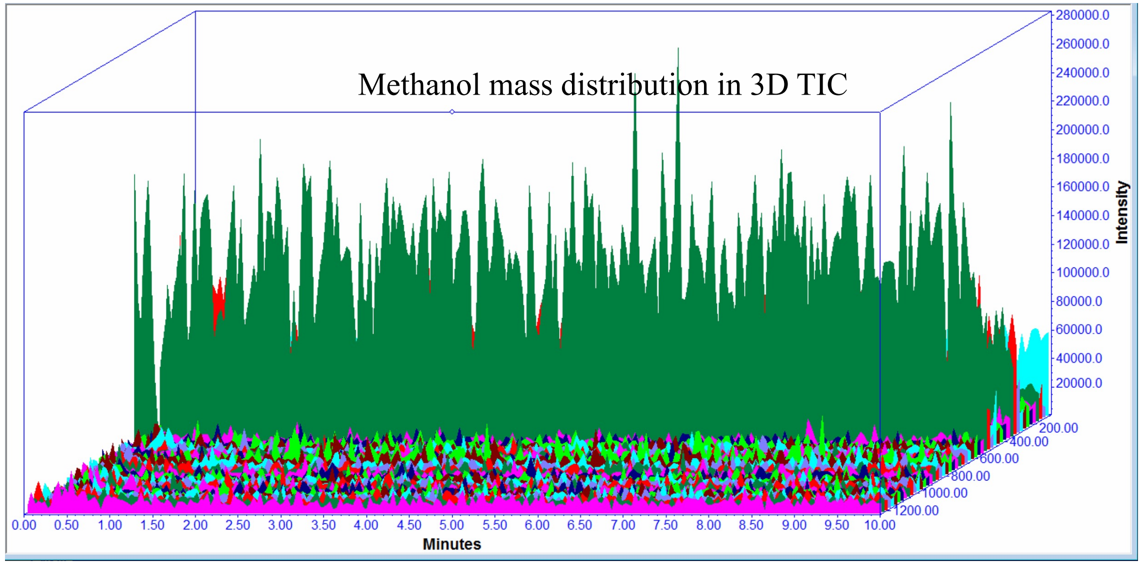

SIR experiment, 3 channels selected for methanol, negative mode (ESI-)

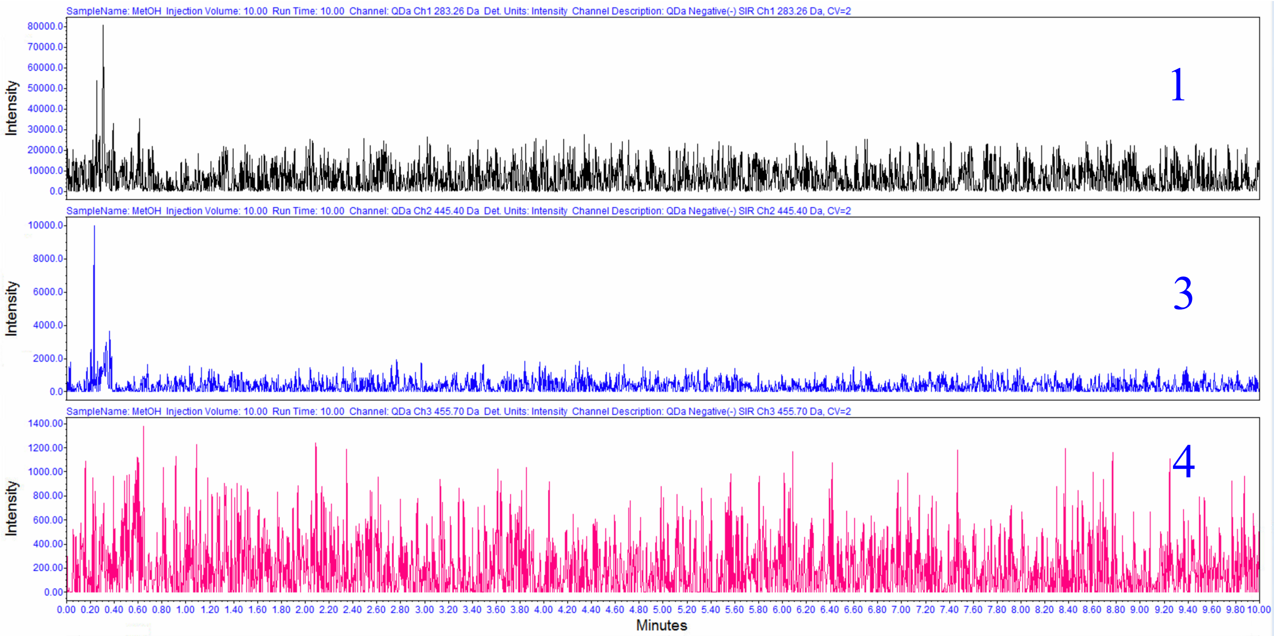

Table of the compound's names for SIR experiment

	**Mass channel selection/SIR**	**Compound name targeted**
1	QDa Ch1 283.26 Da	Acacetin
2	QDa Ch2 285.26 Da	Luteolin NOT SELECTED
3	QDa Ch3 445.40 Da	Tilianin
4	QDa Ch9 455.70 Da	Ursolic acid

Figure S17: Chromatographic experiments for blank of methanol in negative mode (ESI-) chromatographic column ACQUITY UPLC® HSS T3 130 Å (1.8 µm, 2.1 × 100 mm).

CHROMATOGRAPHIC EXPERIMENTS IN ACQUITY UPLC® HSS T3 130 Å (1.8 µm, 2.1 × 100 mm) COLUMN

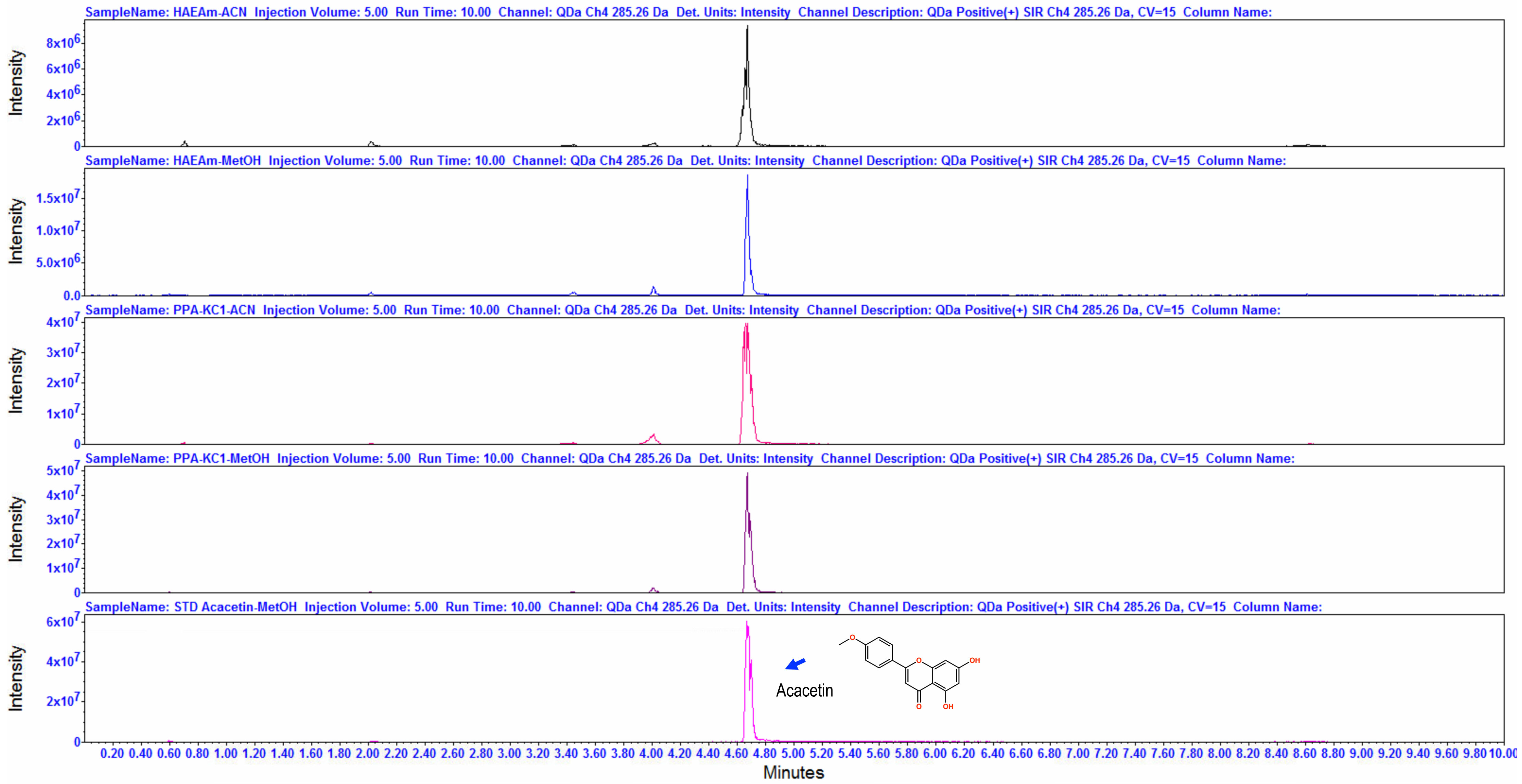

Figure S18: Selected Ion Recording (SIR) experiment, channel selected for Acacetin mass at 285.26 Da m/z [M+1], in standard and samples, positive mode (ESI+) [the X-axis represents time in minutes, and Y-axis represents signal intensity]. Gradient elution, column ACQUITY UPLC® HSS T3 130 Å (1.8 µm, 2.1 × 100 mm).

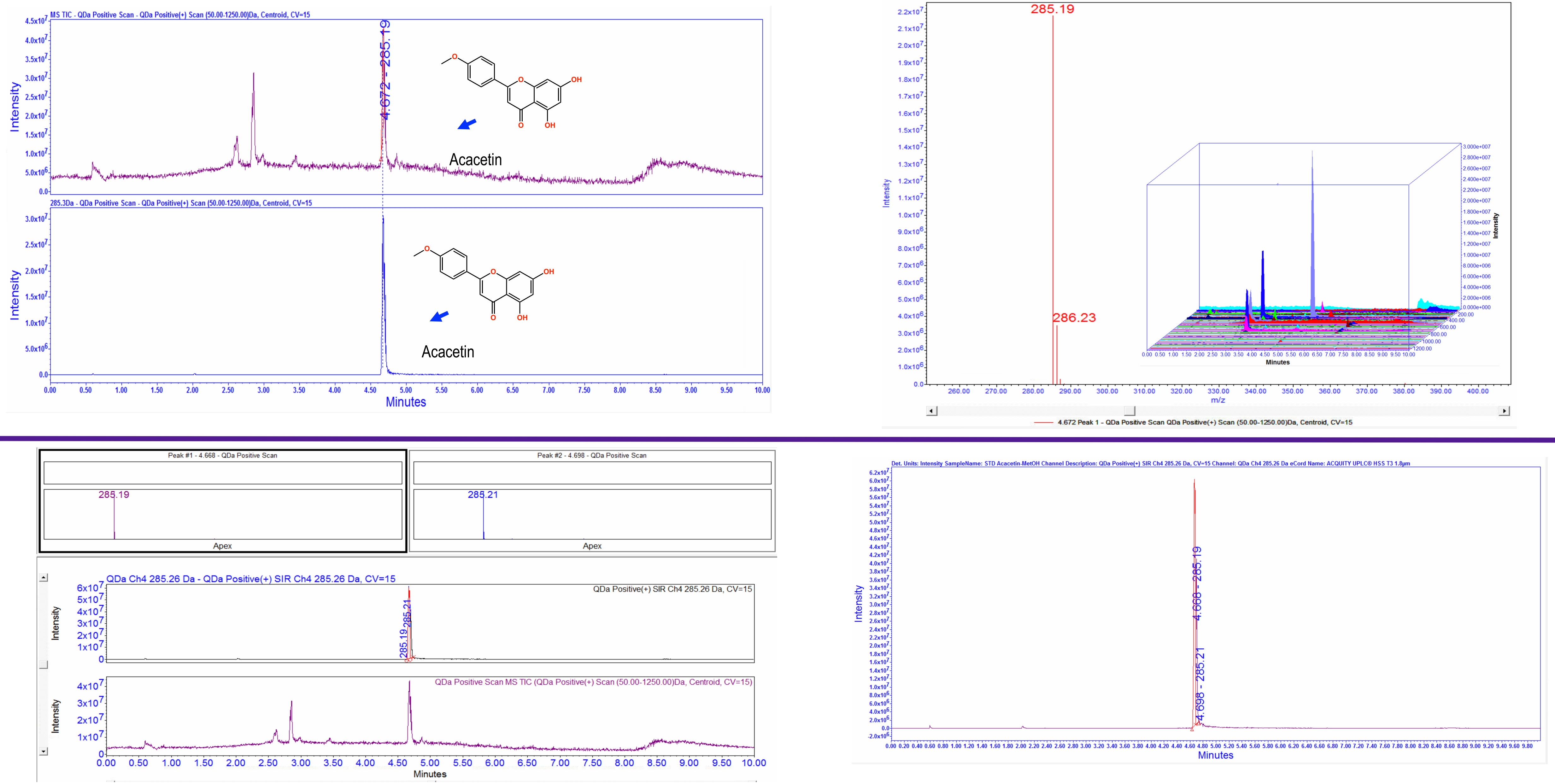

Figure S19: Selected Ion Recording (SIR) experiment, channel selected for Acacetin standard mass at 285.26 Da m/z [M+1], positive mode (ESI+) [the X-axis represents time in minutes, and Y-axis represents signal intensity]. Gradient elution, column ACQUITY UPLC® HSS T3 130 Å (1.8 μm, 2.1 × 100 mm).

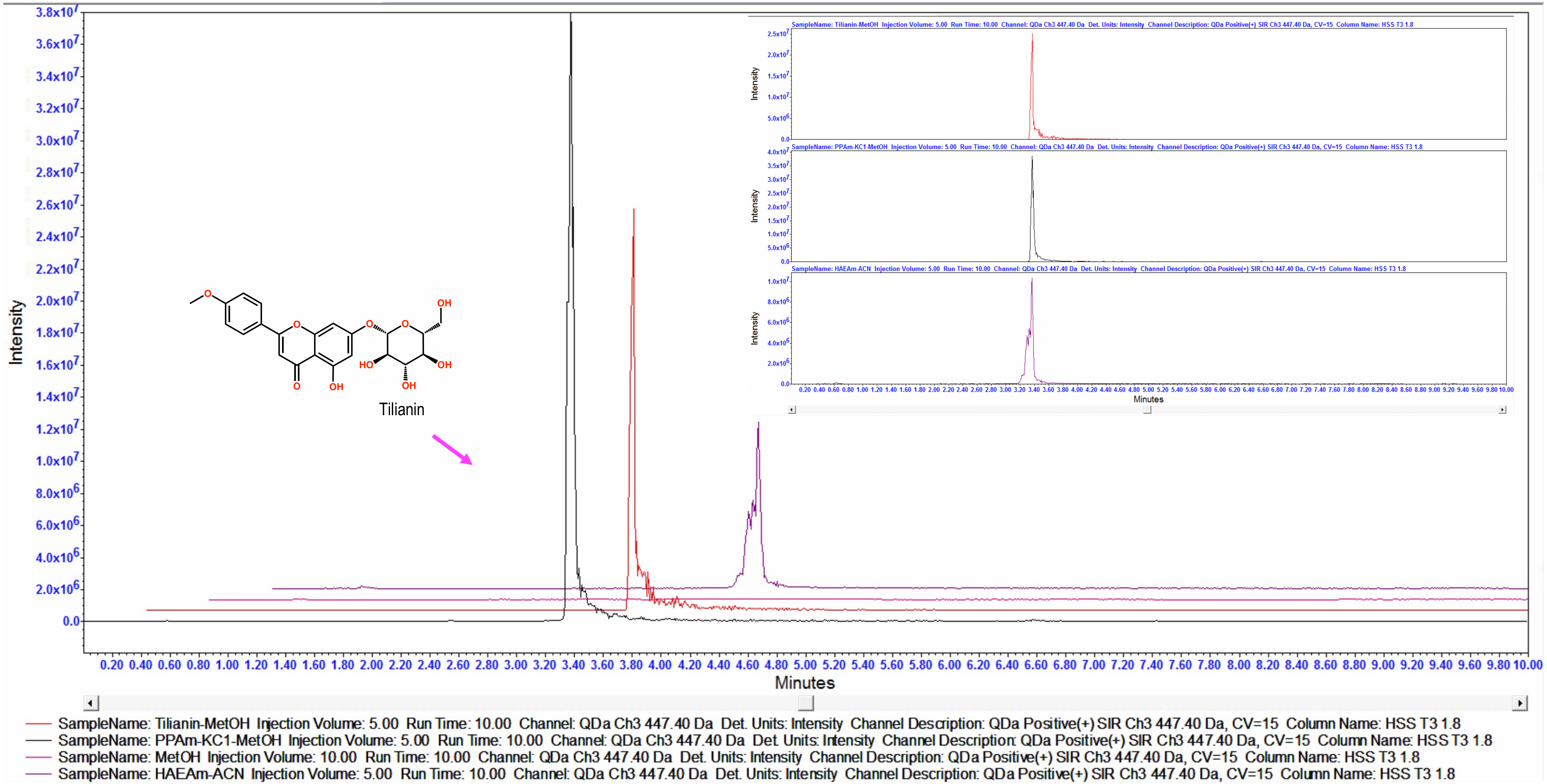

Figure S20: Selected Ion Recording (SIR) experiment, channel selected for Tilianin mass at 447.40 Da m/z [M+1], positive mode (ESI+) for Standard, HAE*Am* and PP*Am* [the X-axis represents time in minutes, and Y-axis represents signal intensity]. Gradient elution, column ACQUITY UPLC® HSS T3 130 Å (1.8 µm, 2.1 × 100 mm).

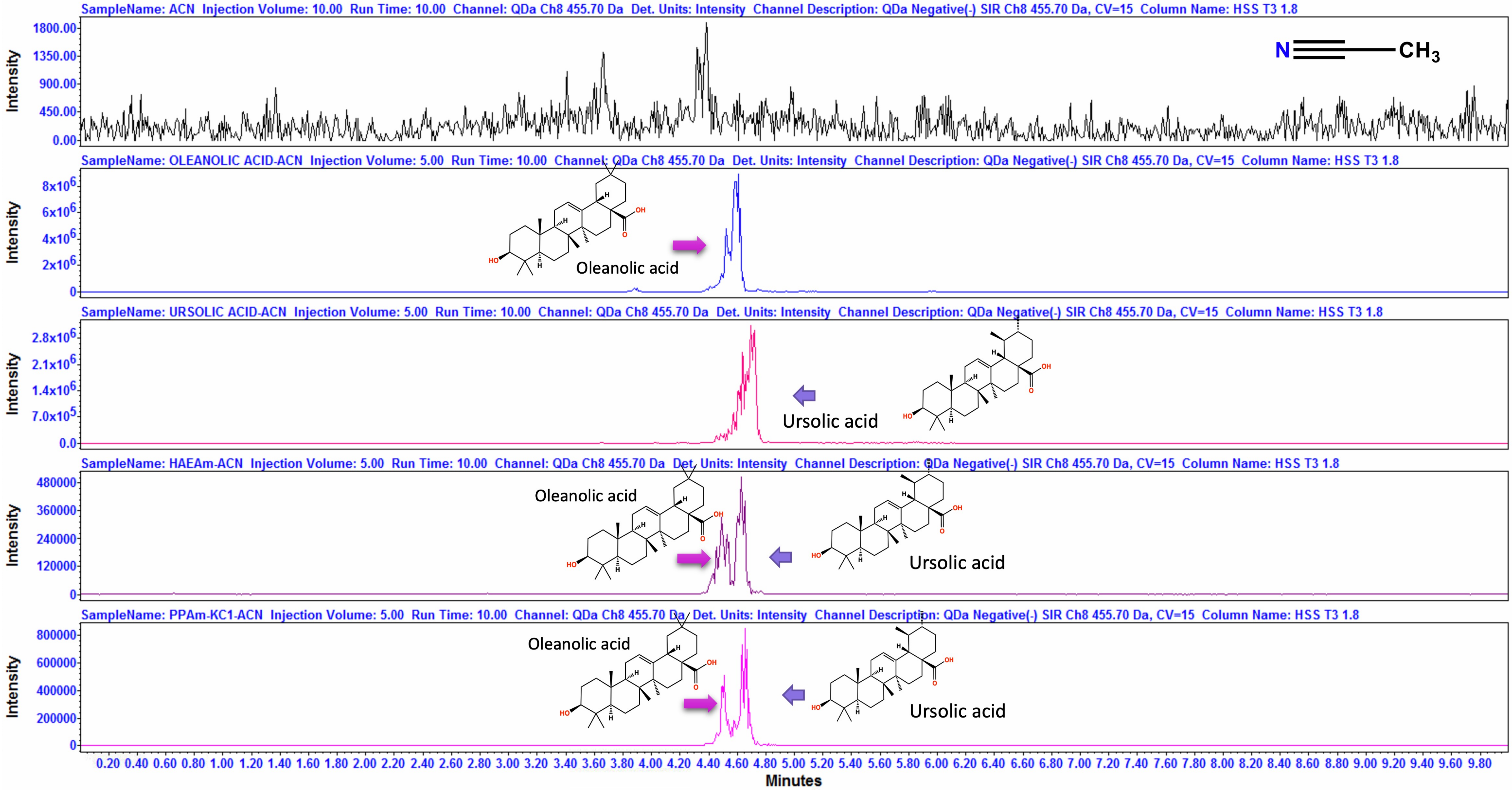

Figure S21: Stack plot from Selected Ion Recording (SIR) experiment, channel selected for **ursolic acid** and **oleanolic acid** mass at 455.70 Da m/z [M-1], negative mode (ESI-) for Standard, HAE*Am* and PP*Am* [the X-axis represents time in minutes, and Y-axis represents signal intensity]. Gradient elution, column ACQUITY UPLC® HSS T3 130 Å (1.8 µm, 2.1 × 100 mm).

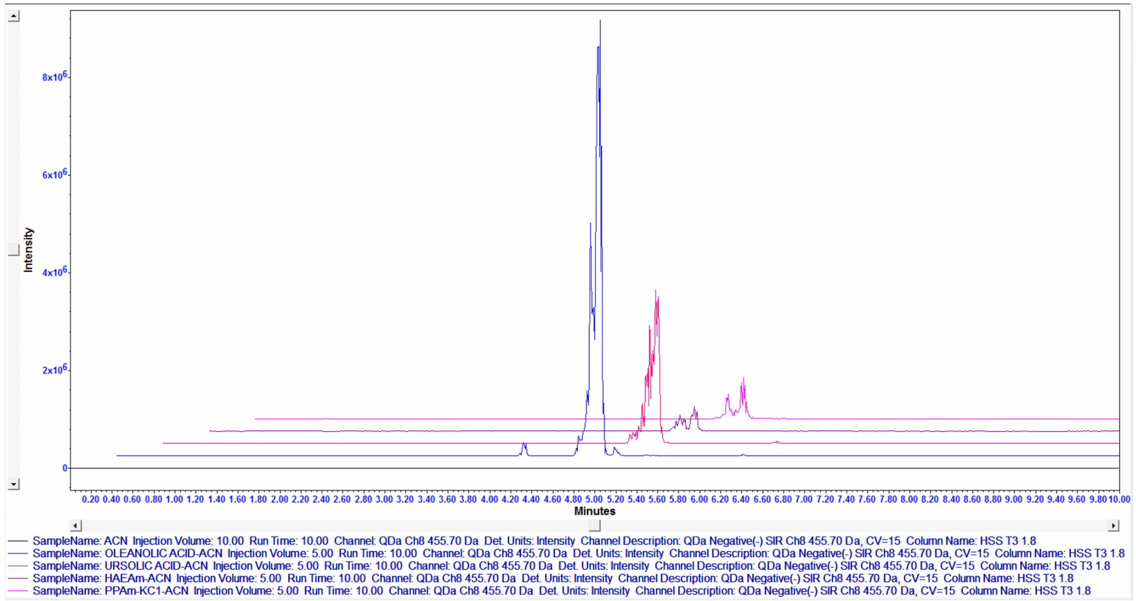

	Mass channel selection/SIR ESI-	Compound name targeted	Retention time isocratic mode		Retention time gradient mode	
			PPam	Standard	PPam	Standard
1	285.24 Da	Luteolin	0.521	0.583	2.81	2.81
2	283.26 Da	Acacetin	0.718	0.93	2.87	2.83
3	445.40 Da	Tilianin	ND	ND	ND	ND
4	455.7 Da	Oleanolic acid	2.27	2.12	4.5	4.47
5	455.7 Da	Ursolic acid	2.35	2.39	4.65	4.65

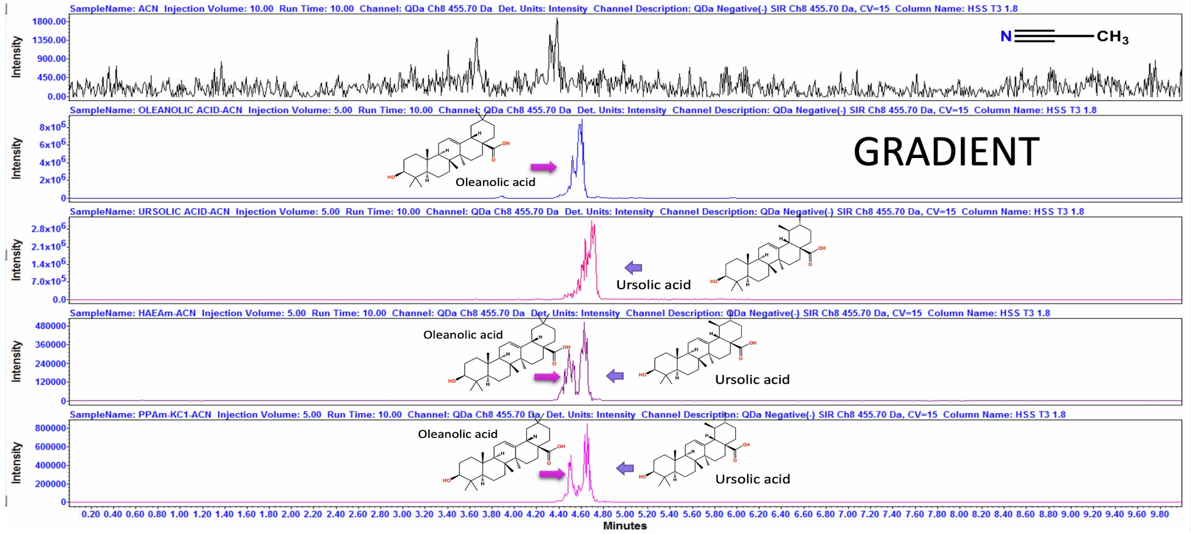

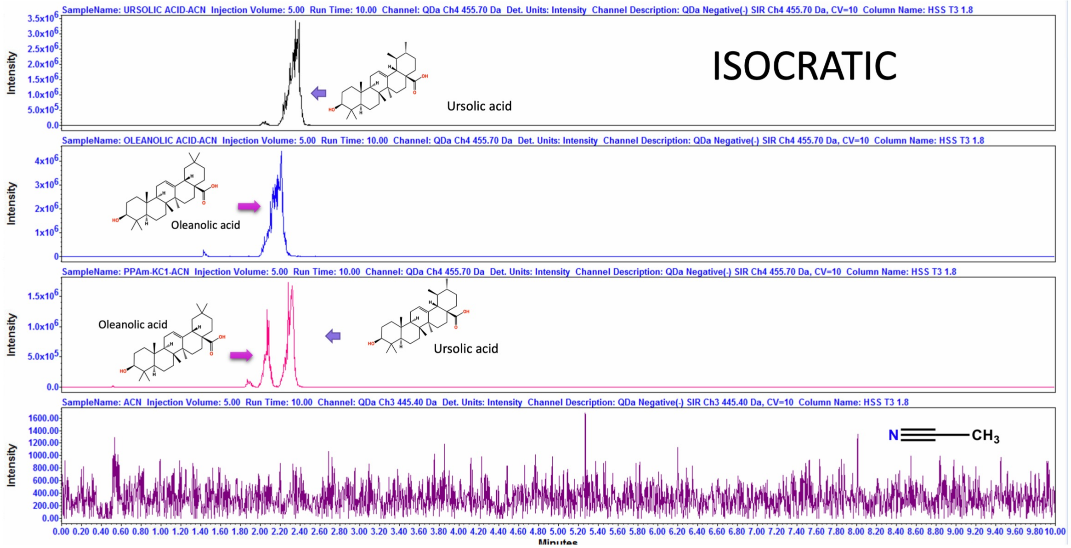

Figure S22: B), C) Stack plot from Selected Ion Recording (SIR) experiment, channel selected for ursolic acid and oleanolic acid mass at 455.70 Da m/z [M-1], negative mode (ESI-) for Standard, HAE*Am* and PP*Am*, *g*gradient mode elution for B) and isocratic for A). [the X-axis represents time in minutes, and Y-axis represents signal intensity]. Column ACQUITY UPLC® HSS T3 130 Å (1.8 µm, 2.1 × 100 mm).

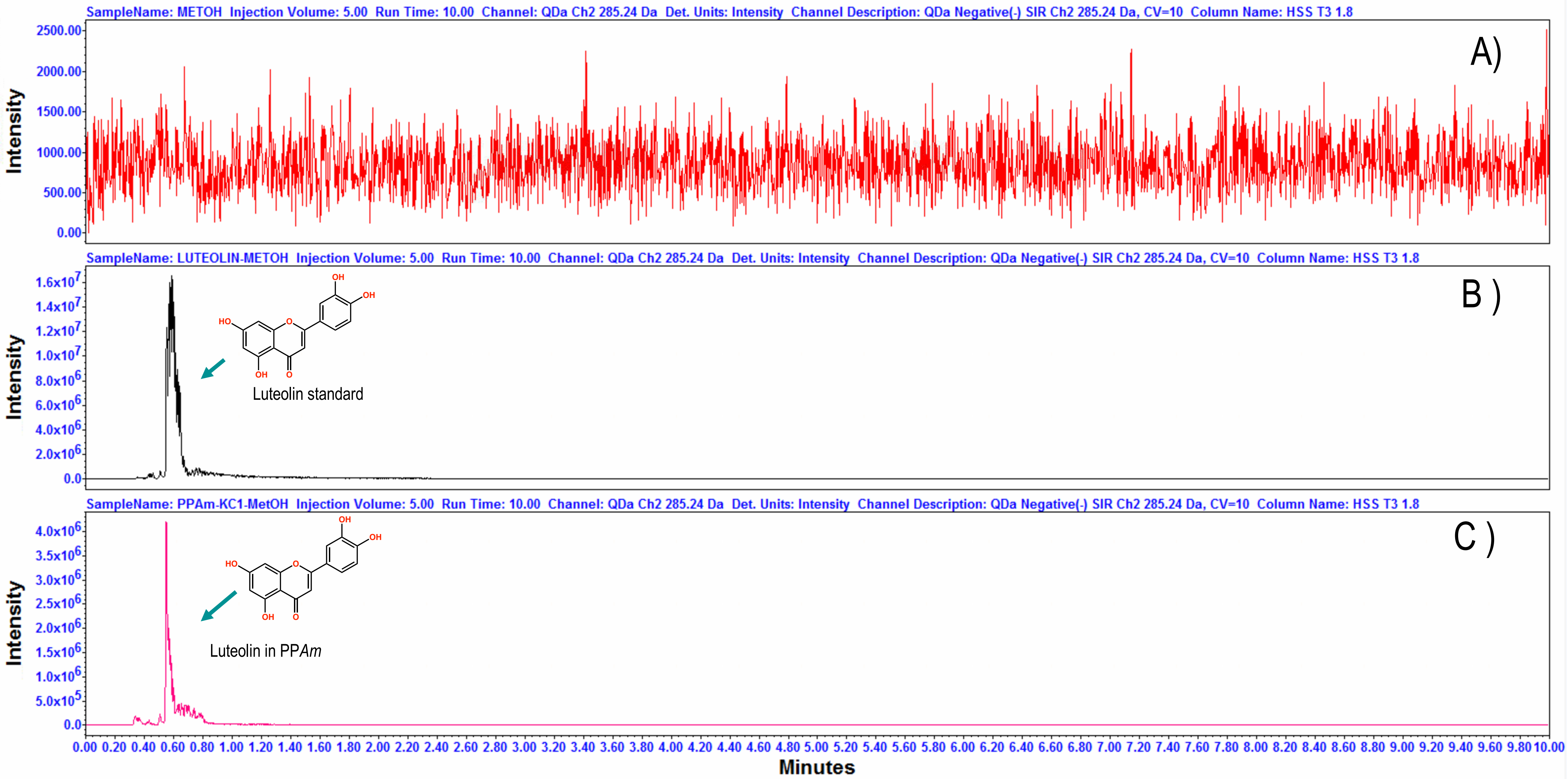

Figure S23: Selected Ion Recording (SIR) experiment, channel selected for Luteolin mass at 285.24 Da m/z [M-1], negative mode (ESI-) A) blank methanol B)Luteolin commercial standard in methanol retention time 0.583 C) PP*Am* in methanol retention time 0.521[the X-axis represents time in minutes, and Y-axis represents signal intensity]. Isocaratic elution, column ACQUITY UPLC® HSS T3 130 Å (1.8 µm, 2.1 × 100 mm).

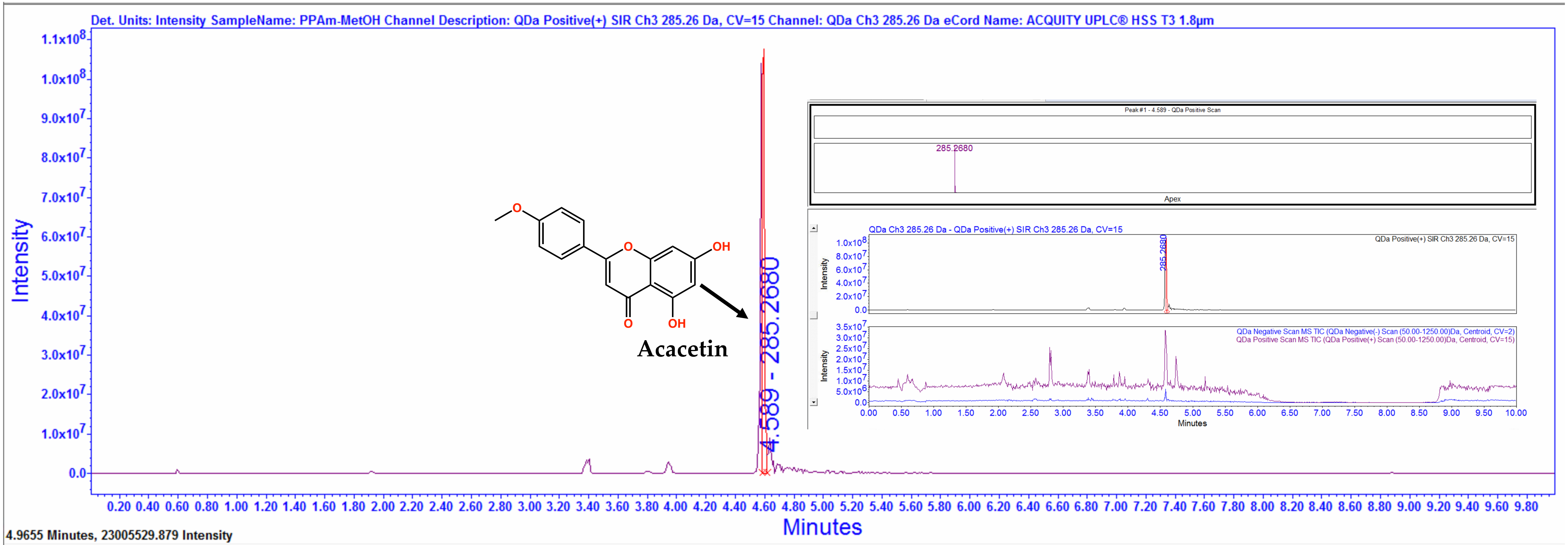

	Retention Time (min)	Area (µV*sec)	% Area	% Height	Height (µV)	Int Type	Start Time (min)	End Time (min)	Base Peak (m/z)	Baseline Start (min)	Baseline End (min)	Slope	MS 3D Channel Name	Offset	RF	Width (sec)
1	4.589	97761241	100.00	100.00	116084041	VV	4.584	4.618	285.2680	4.524	4.669	4.115232e+005	QDa Positive Scan	-1.765884e+006		2.039

Figure S24 : Selected Ion Recording (SIR) experiment for PP*Am* in methanol, channel selected for acacetin mass targeted at 285.26 Da m/z [M+1], positive mode (ESI+) mass detected in PP*Am* 285.2680 at 4.589 minuteds [the X-axis represents time in minutes, and Y-axis represents signal intensity]. Gradient elution, column ACQUITY UPLC® HSS T3 130 Å (1.8 µm, 2.1 × 100 mm).

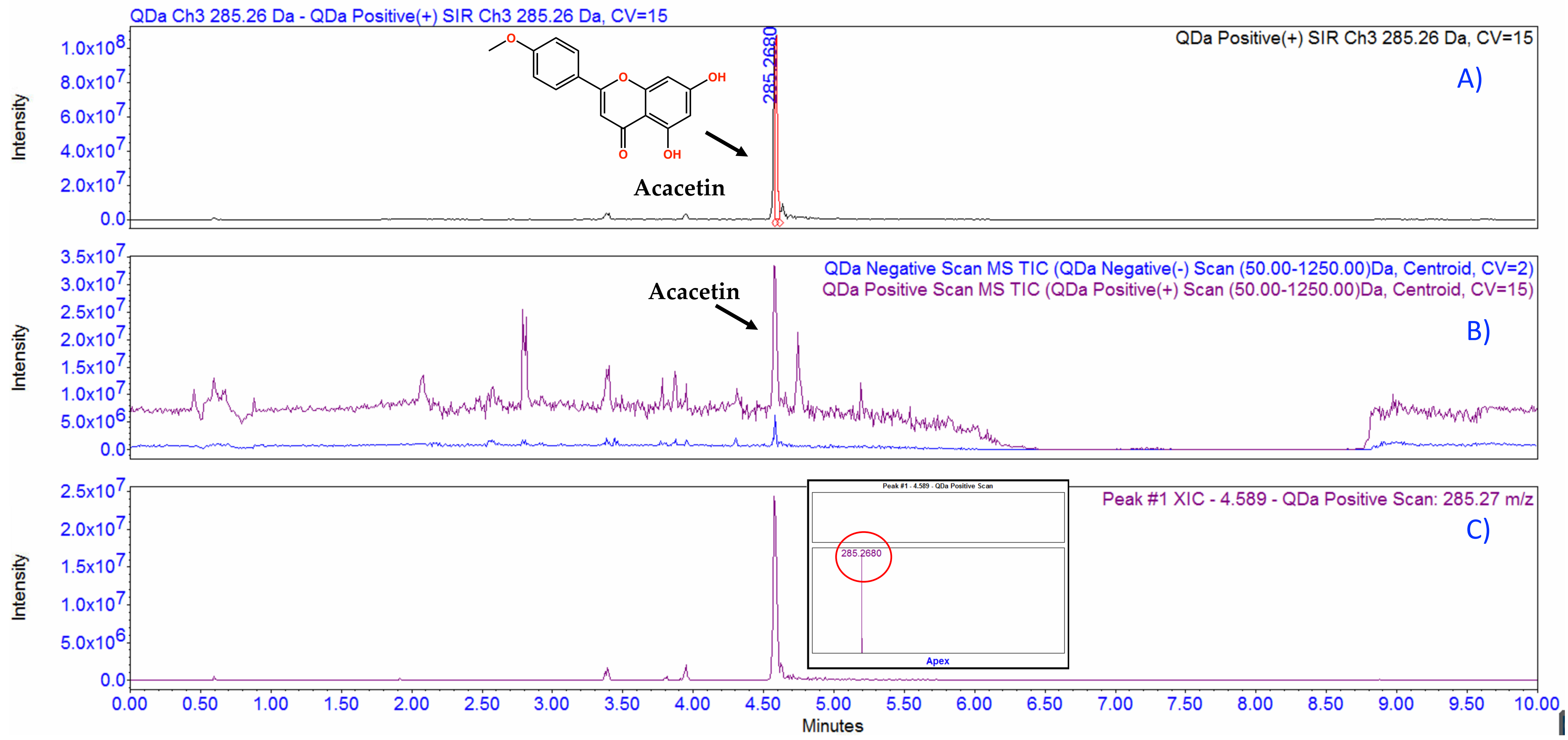

Figure S25: Stack plot for mass analysis for PP*Am* in methanol. **A)**SIR experiment, mass targeted 285.26**, mass detected 285.2680 B)** total ion chromatogram, exploratory mass scan from 50-1250, Selected channels Da m/Z [ESI+] from PP*Am* showing the presence of acacetin in the sample at 4.589 minutes **C)** Extracted ion chromatograms (XIC) at 4.589 minutes, the X-axis represents time in minutes, and Y-axis represents signal intensity. Column ACQUITY UPLC® HSS T3 130 Å (1.8 μm, 2.1 × 100 mm).

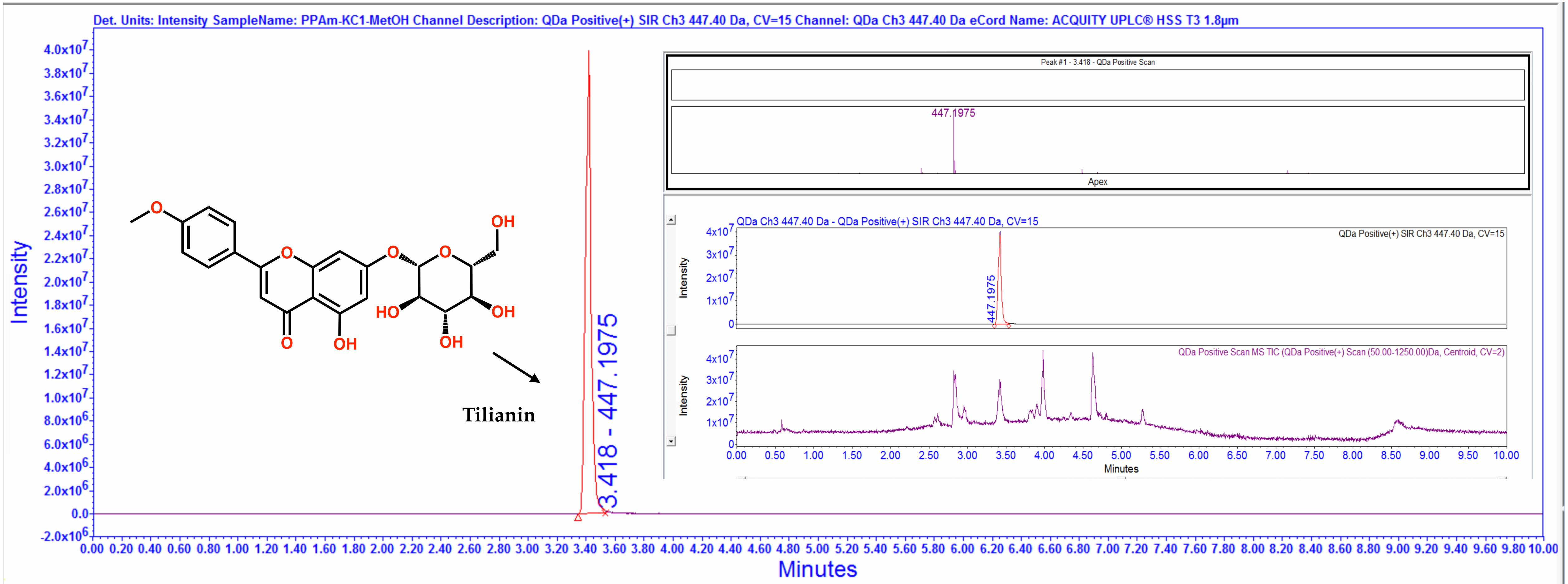

	Retention Time (min)	Area (µV*sec)	% Area	% Height	Height (µV)	Int Type	Start Time (min)	End Time (min)	Base Peak (m/z)	Baseline Start (min)	Baseline End (min)	Slope	MS 3D Channel Name	Offset	RF	Width (sec)	Inflection Width (sec)	2[illegible] Deriv[illegible] Ap[illegible] (m[illegible]
1	3.418	108487943	100.00	100.00	39417508	BV	3.343	3.533	447.1975	3.343	3.599	2.395932e+005	QDa Positive Scan	-7.761199e+005		11.381	1.118	

Figure S26 : Selected Ion Recording (SIR) experiment for PP*Am* in methanol, channel selected for acacetin mass targeted at447.40 Da m/z [M+1], positive mode (ESI+) mass detected in PP*Am* 447.1975 at 3.418 minutes [the X-axis represents time in minutes, and Y-axis represents signal intensity]. Gradient elution, column ACQUITY UPLC® HSS T3 130 Å (1.8 µm, 2.1 × 100 mm).

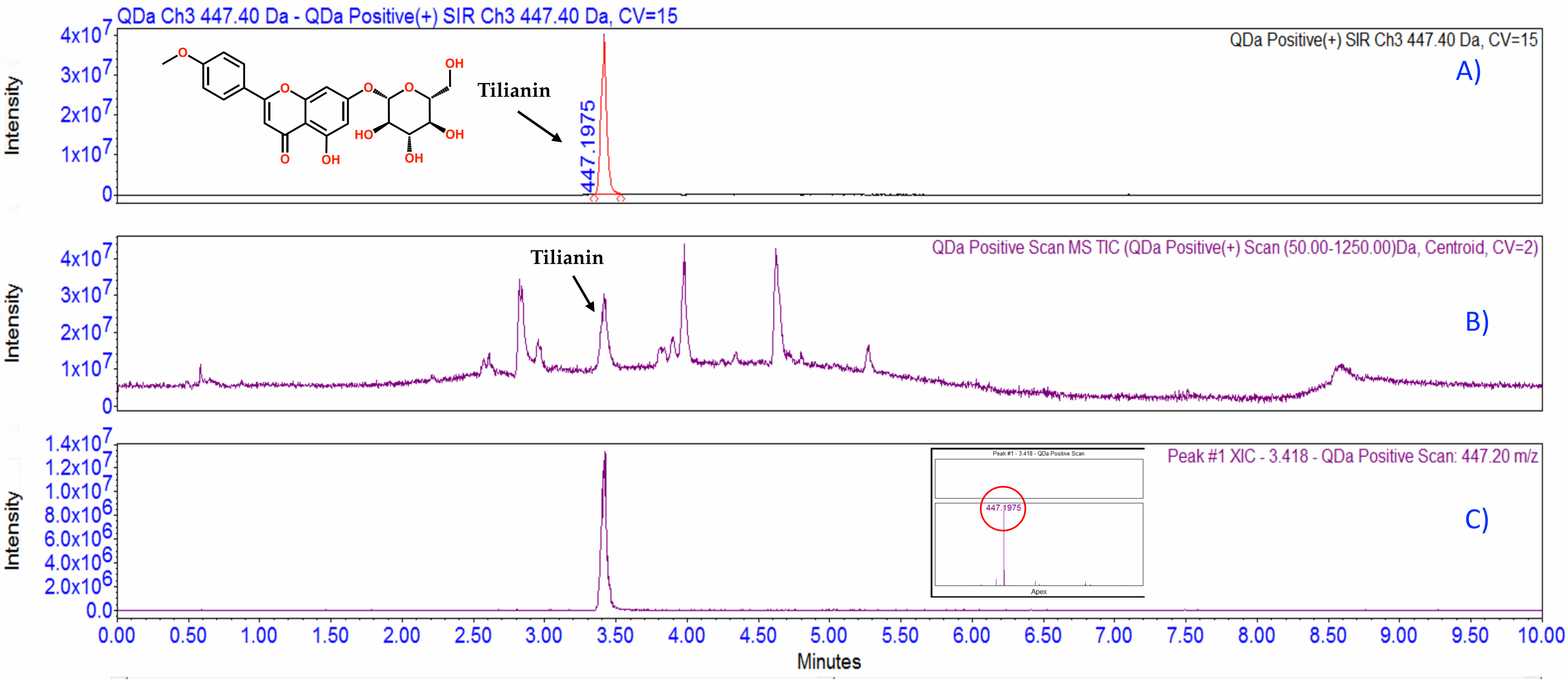

Figure S27: Stack plot for mass analysis for PP*Am* in methanol. **A)**SIR experiment, mass targeted 447.40 for tilianin identification, mass detected 447.1975. **B)** total ion chromatogram, exploratory mass scan from 50-1250, Selected channels Da m/Z [ESI+] from PP*Am* showing the presence of tilianin in the sample **C)** Extracted ion chromatograms (XIC) at 3.418 minutes in [the X-axis represents time in minutes, and Y-axis represents signal intensity]. Column ACQUITY UPLC® HBE C18 130 Å column (1.7 µm, 2.1 x 50mm).

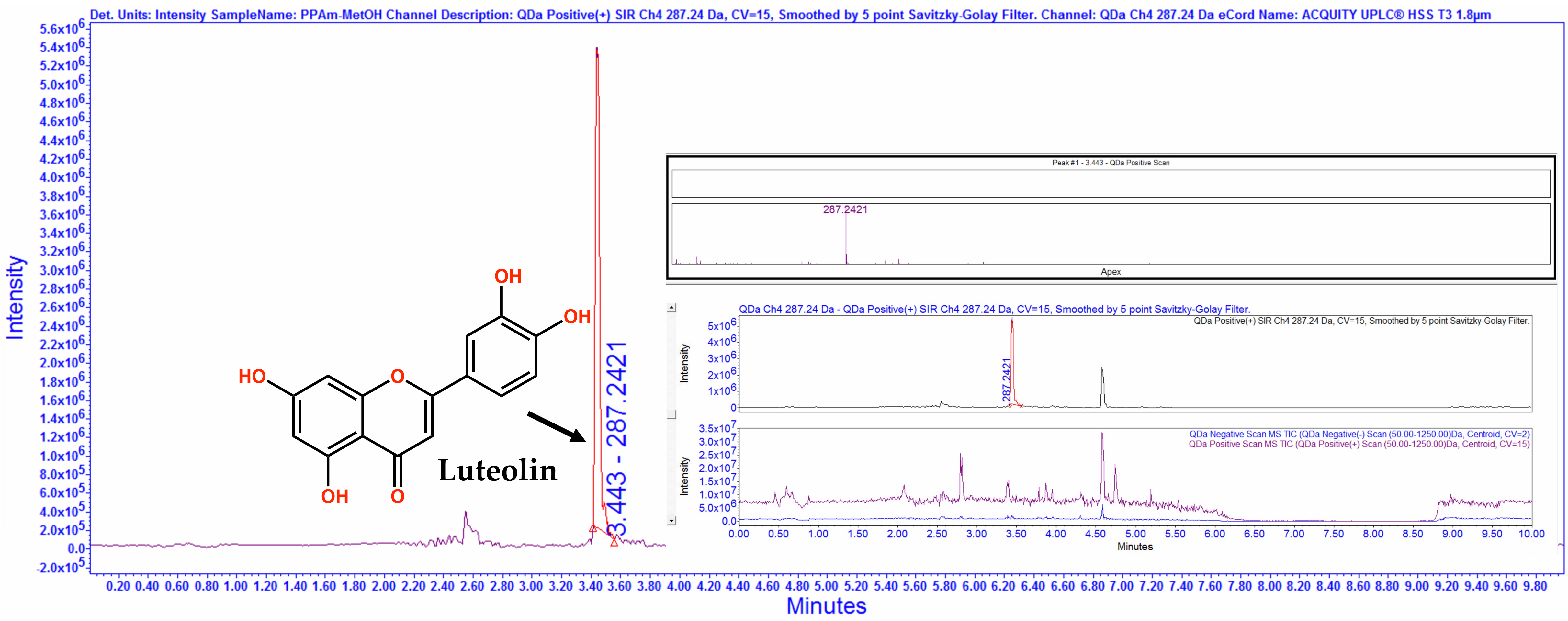

	Retention Time (min)	Area (µV*sec)	% Area	% Height	Height (µV)	Int Type	Start Time (min)	End Time (min)	Base Peak (m/z)	Baseline Start (min)	Baseline End (min)	Slope	MS 3D Channel Name	Offset	RF	Width (sec)	In
1	3.443	11423094	100.00	100.00	5214106	BB	3.412	3.556	287.2421	3.412	3.556	-1.064387e+006	QDa Positive Scan	3.894350e+006		8.664	

Figure S28 : Selected Ion Recording (SIR) experiment for PP*Am* in methanol, channel selected for luteolin mass identification targeted at 287.24 Da m/z [M+1], positive mode (ESI+) mass detected in PP*Am* 287.2421 at 3.443 minutes [the X-axis represents time in minutes, and Y-axis represents signal intensity]. Gradient elution, column ACQUITY UPLC® HSS T3 130 Å (1.8 µm, 2.1 × 100 mm).

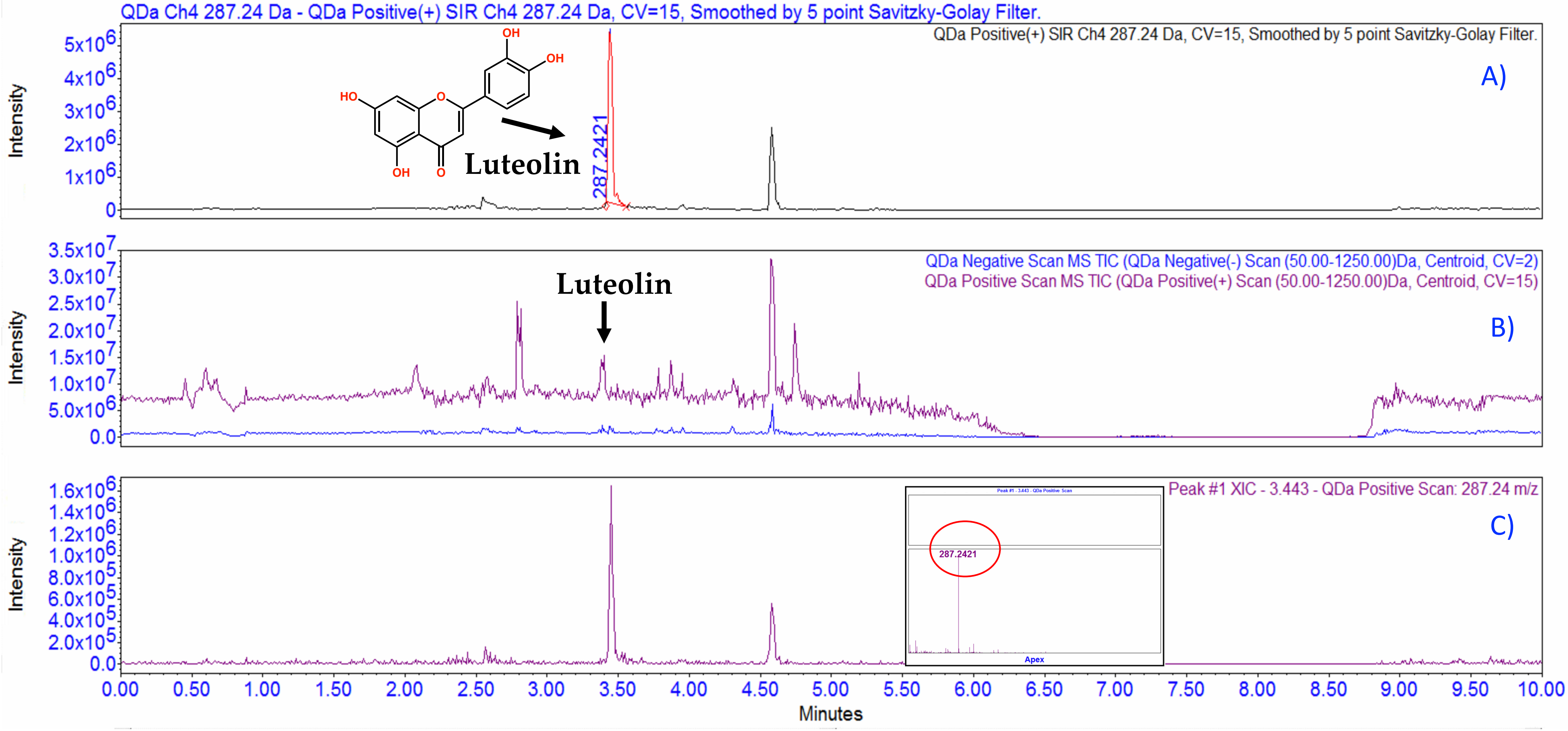

Figure S29: Stack plot for mass analysis for PP*Am* in methanol. **A)**SIR experiment, mass targeted 287.24**, mass detected 287.2421 B)** total ion chromatogram, exploratory mass scan from 50-1250, Selected channels Da m/Z [ESI+] from PP*Am* showing the prescence of **luteolin** in the sample at 3.443 minutes **C)** Extracted ion chromatograms (XIC) at 3.443 minutes, the X-axis represents time in minutes, and Y-axis represents signal intensity. Column ACQUITY UPLC® HSS T3 130 Å (1.8 µm, 2.1 × 100 mm).

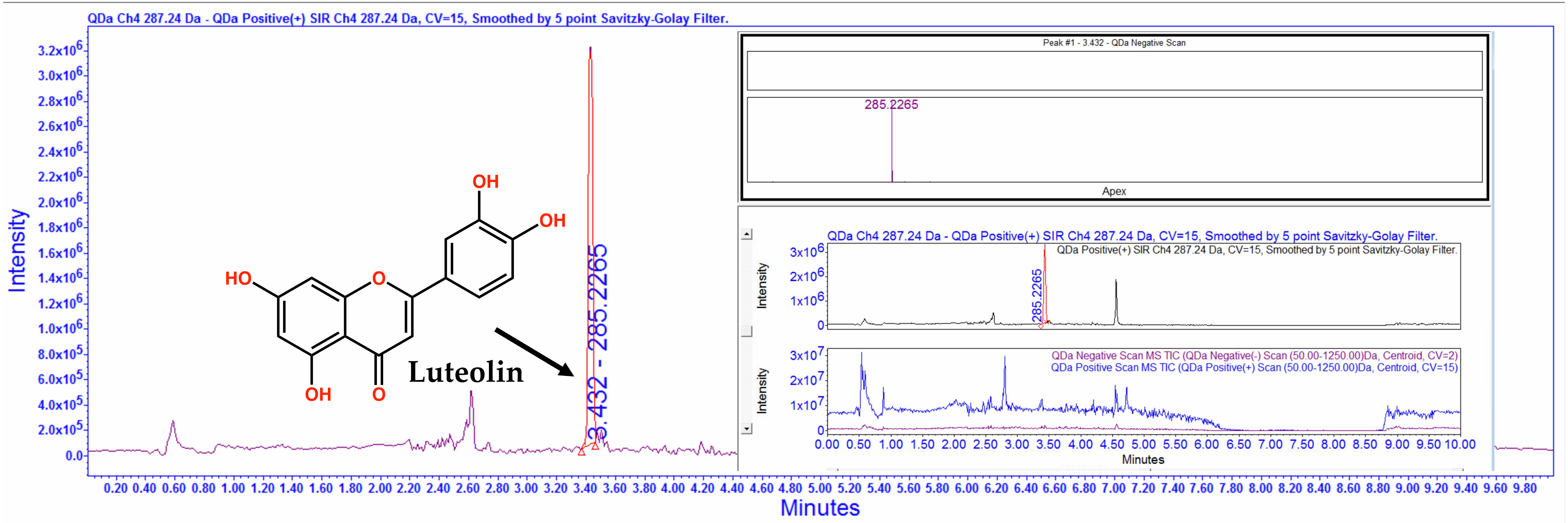

	Base Peak (m/z)	Baseline Start (min)	Baseline End (min)	Slope	MS 3D Channel Name	Offset	RF	Width (sec)	Inflection Width (sec)	2nd Derivative Apex (min)	Scan Number	MS 3D Channel Id	MS 3D Channel Description
1	285.2265	3.369	3.463	5.409736e+005	QDa Negative Scan	-1.764281e+006		5.607	2.206	3.433	404	34188	QDa Negative(-) Scan (50.00-1250.

Figure S30 : Selected Ion Recording (SIR) experiment for HAE*Am* in methanol, channel selected for **luteolin** mass identification targeted at 287.24 Da m/z [M+1], positive mode (ESI+) mass detected in PP*Am* 285.2265 at 3.432 minutes [the X-axis represents time in minutes, and Y-axis represents signal intensity]. Gradient elution, column ACQUITY UPLC® HSS T3 130 Å (1.8 µm, 2.1 × 100 mm).

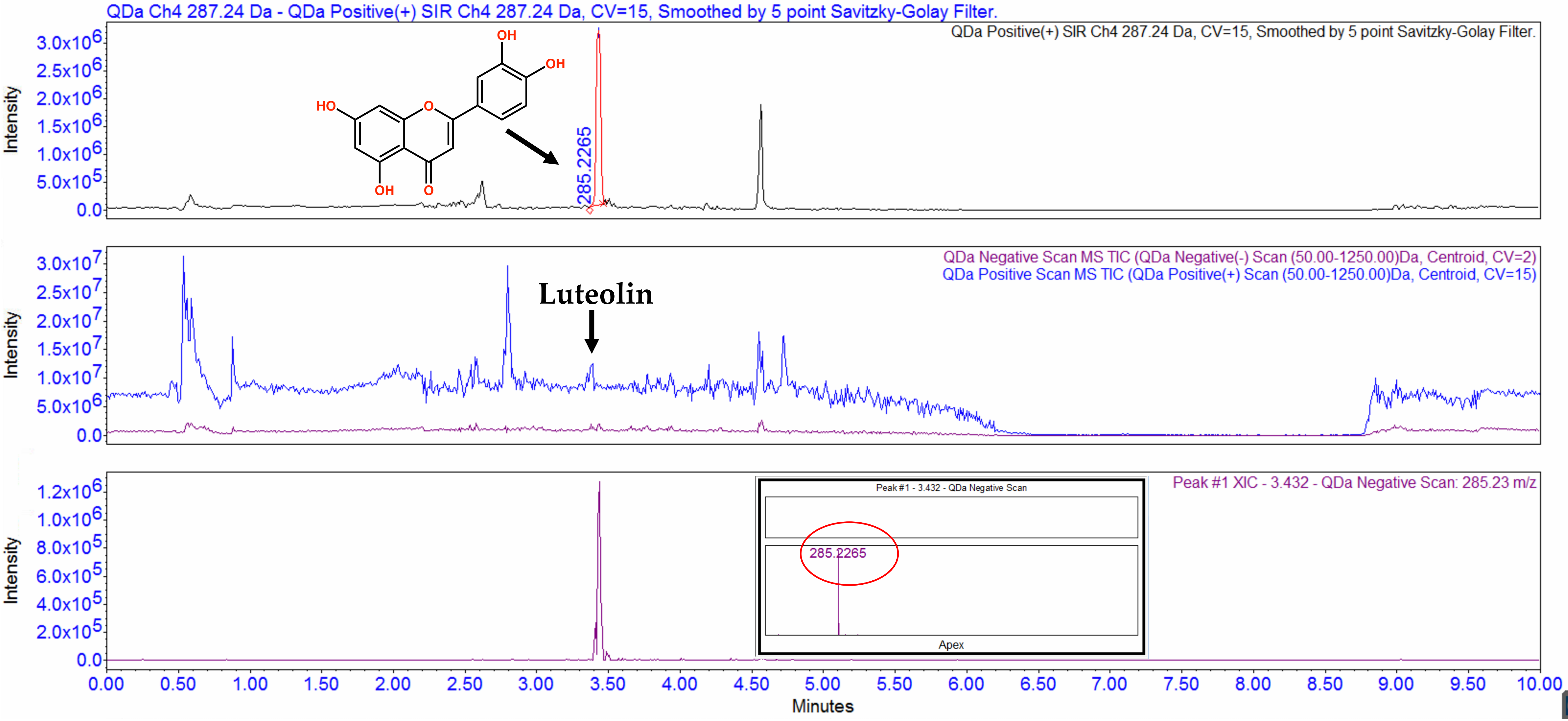

Figure S31: Stack plot for mass analysis for HAE*Am* in methanol. **A)**SIR experiment, mass targeted 287.24 for luteolin identification, mass detected 285.2265 **B)** total ion chromatogram, exploratory mass scan from 50-1250, Selected channels Da m/Z [ESI+] from HAE*Am* showing the presence of **luteolin** in the sample at 3.432 minutes **C)** Extracted ion chromatograms (XIC) at 3.443 minutes, the X-axis represents time in minutes, and Y-axis represents signal intensity. Column ACQUITY UPLC® HSS T3 130 Å (1.8 µm, 2.1 × 100 mm).

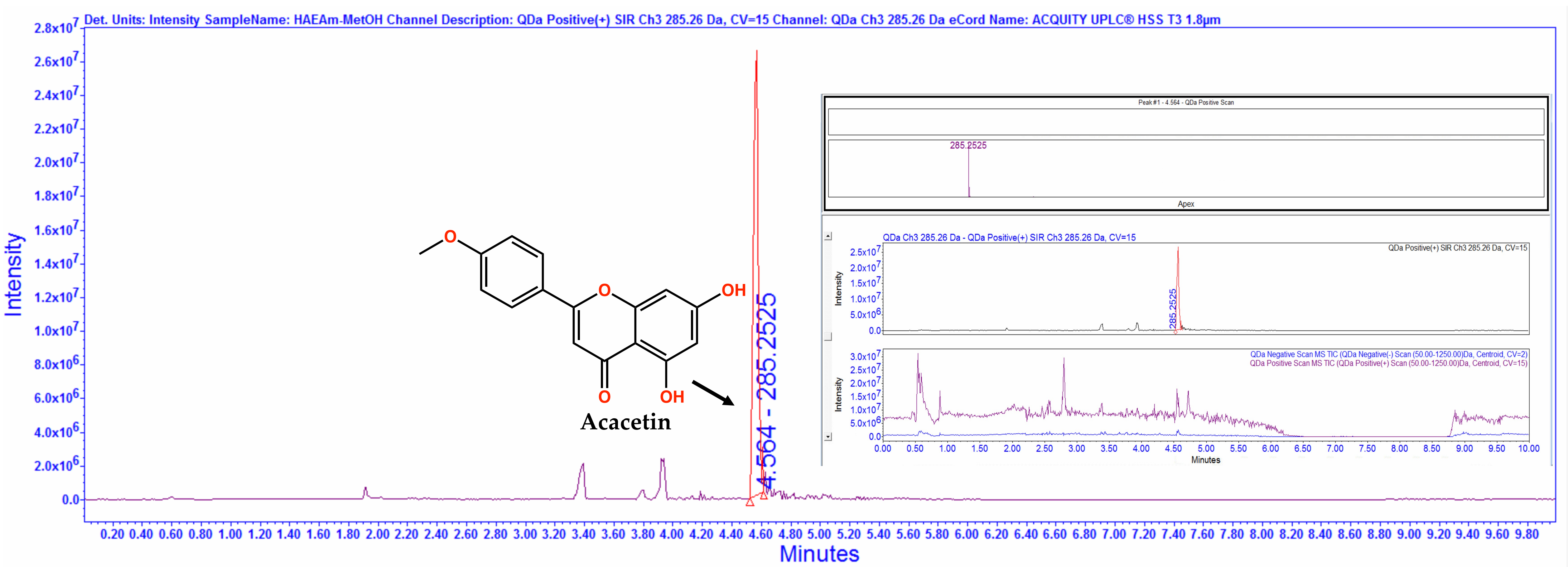

Retention Time (min)	Area (µV*sec)	% Area	% Height	Height (µV)	Int Type	Start Time (min)	End Time (min)	Base Peak (m/z)	Baseline Start (min)	Baseline End (min)	Slope	MS 3D Channel Name	Offset	RF	Width (sec)	Inflection Width (sec)	2nd Derivative Apex (min)	Scan Number	MS 3D Chann Id
4.564	60121625	100.00	100.00	26060135	BB	4.525	4.618	285.2525	4.525	4.618	4.274632e+006	QDa Positive Scan	-1.926076e+007		5.608	2.383	4.567	538	34

Figure S32 : Selected Ion Recording (SIR) experiment for HAE*Am* in methanol, channel selected for **acacetin** mass targeted at 285.26 Da m/z [M+1], positive mode (ESI+) mass detected in HAE*Am* 285.2525 [the X-axis represents time in minutes, and Y-axis represents signal intensity]. Gradient elution, column ACQUITY UPLC® HSS T3 130 Å (1.8 µm, 2.1 × 100 mm).

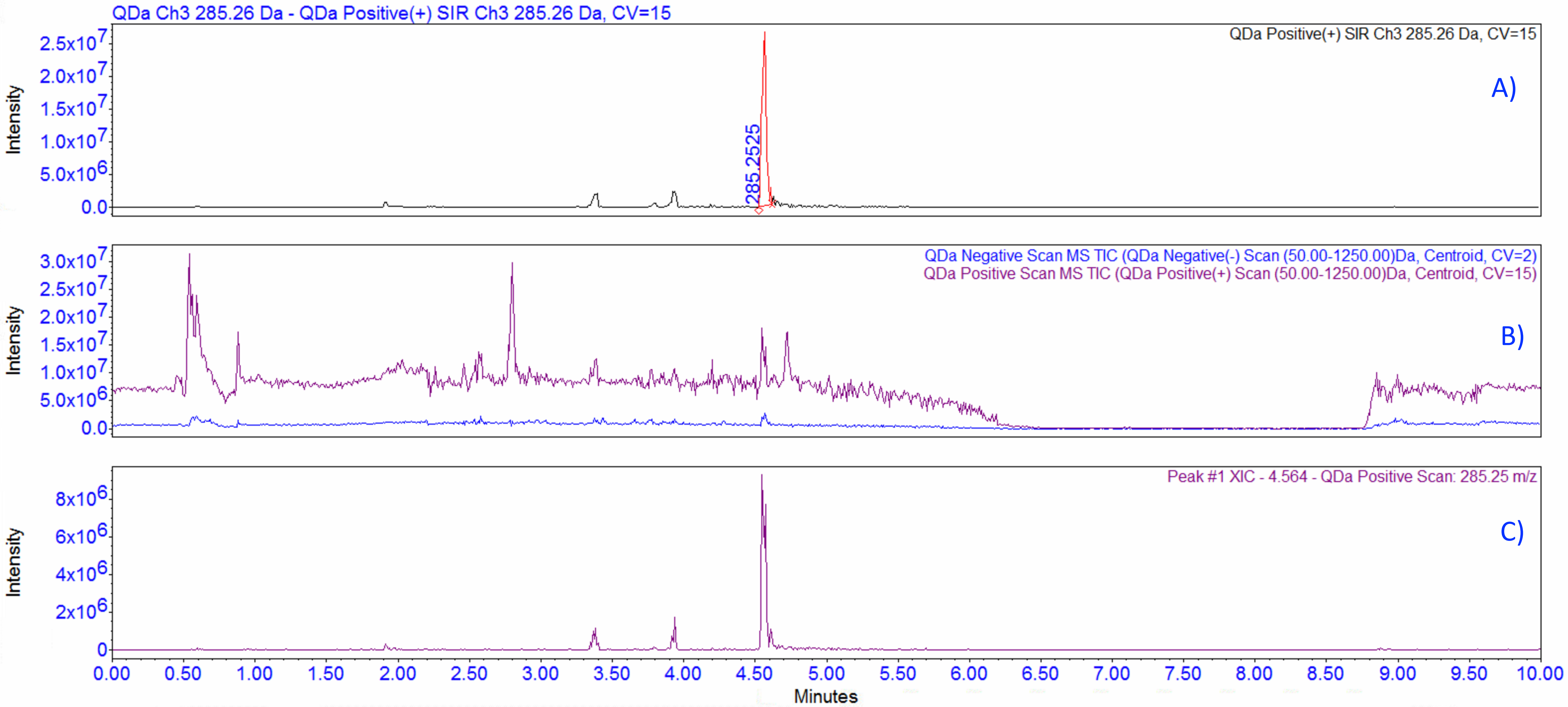

Figure S33: Stack plot for mass analysis for HAE*Am* in methanol. **A)**SIR experiment for acacetin detection, mass targeted 285.26**,** mass detected 285.2525 **B)** total ion chromatogram, exploratory mass scan from 50-1250, Selected channels Da m/Z [ESI+/ESI-] from HAE*Am* showing the presence of acacetin in the sample at 4.564 minutes **C)** Extracted ion chromatograms (XIC) at 4.564 minutes, the X-axis represents time in minutes, and Y-axis represents signal intensity. Column ACQUITY UPLC® HSS T3 130 Å (1.8 µm, 2.1 × 100 mm).

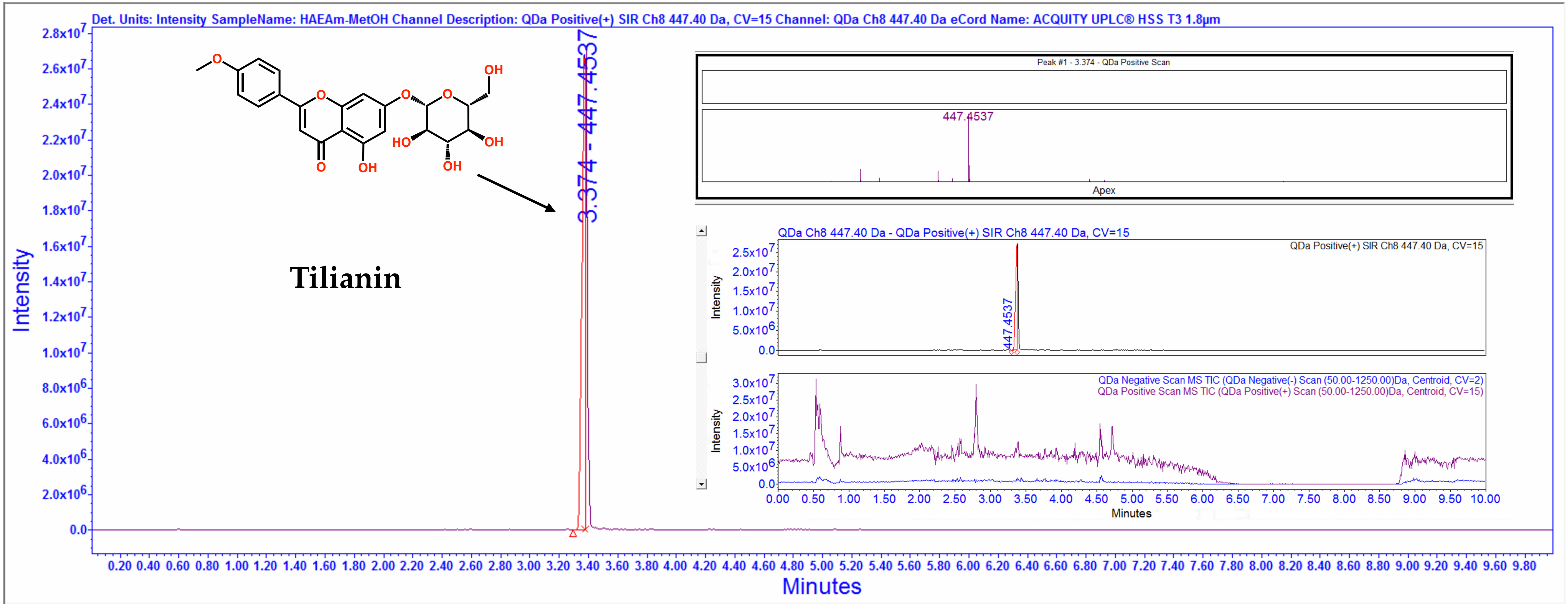

	Retention Time (min)	Area (µV*sec)	% Area	% Height	Height (µV)	Int Type	Start Time (min)	End Time (min)	Base Peak (m/z)	Baseline Start (min)	Baseline End (min)	Slope	MS 3D Channel Name	Offset	RF	Width (sec)	In
1	3.374	39074301	100.00	100.00	27868496	BV	3.294	3.379	447.4537	3.294	3.480	2.638632e+005	QDa Positive Scan	-8.543438e+005		5.098	

Figure S34: Selected Ion Recording (SIR) experiment for HAE*Am* in methanol, channel selected for tilianin mass targeted at 447.40 Da m/z [M+1], positive mode (ESI+) mass detected in HAE*Am* 447.4537 at 3.374 minutes [the X-axis represents time in minutes, and Y-axis represents signal intensity]. Gradient elution, column ACQUITY UPLC® HSS T3 130 Å (1.8 µm, 2.1 × 100 mm).

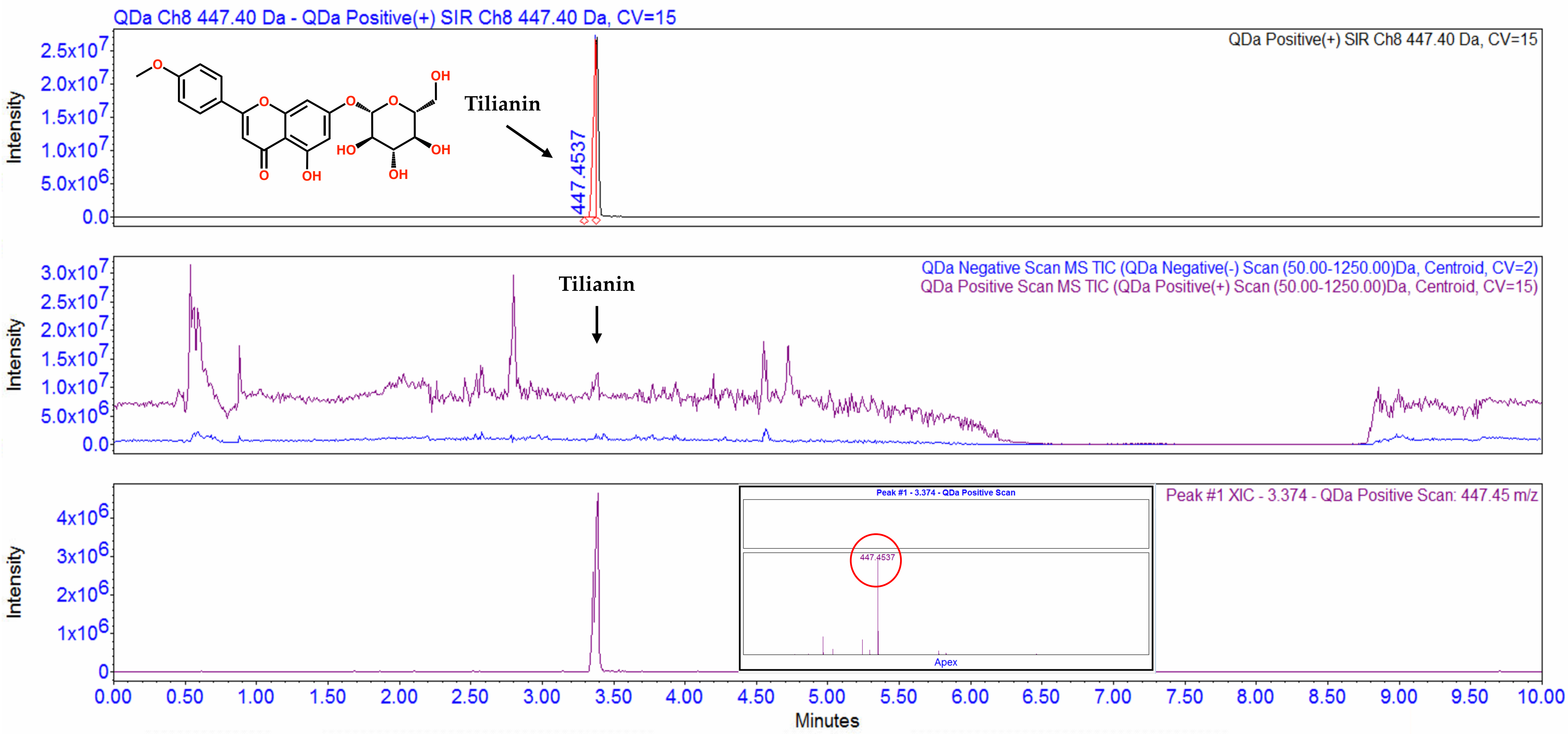

Figure S35: Stack plot for mass analysis for PP*Am* in methanol. **A)**SIR experiment, mass targeted 447.40**, mass detected 447.1975. B)** total ion chromatogram, exploratory mass scan from 50-1250, Selected channels Da m/Z [ESI+] from PP*Am* showing the presence of tilianin in the extract at 3.374 minutes **C)** Extracted ion chromatograms (XIC) at 3.374 minutes in [the X-axis represents time in minutes, and Y-axis represents signal intensity]. Column ACQUITY UPLC® HSS T3 130 Å (1.8 μm, 2.1 × 100 mm).

CHROMATOGRAPHIC EXPERIMENTS IN ACQUITY ACQUITY UPLC® HBE C18 130 Å column (1.7 µm, 2.1 x 50mm).

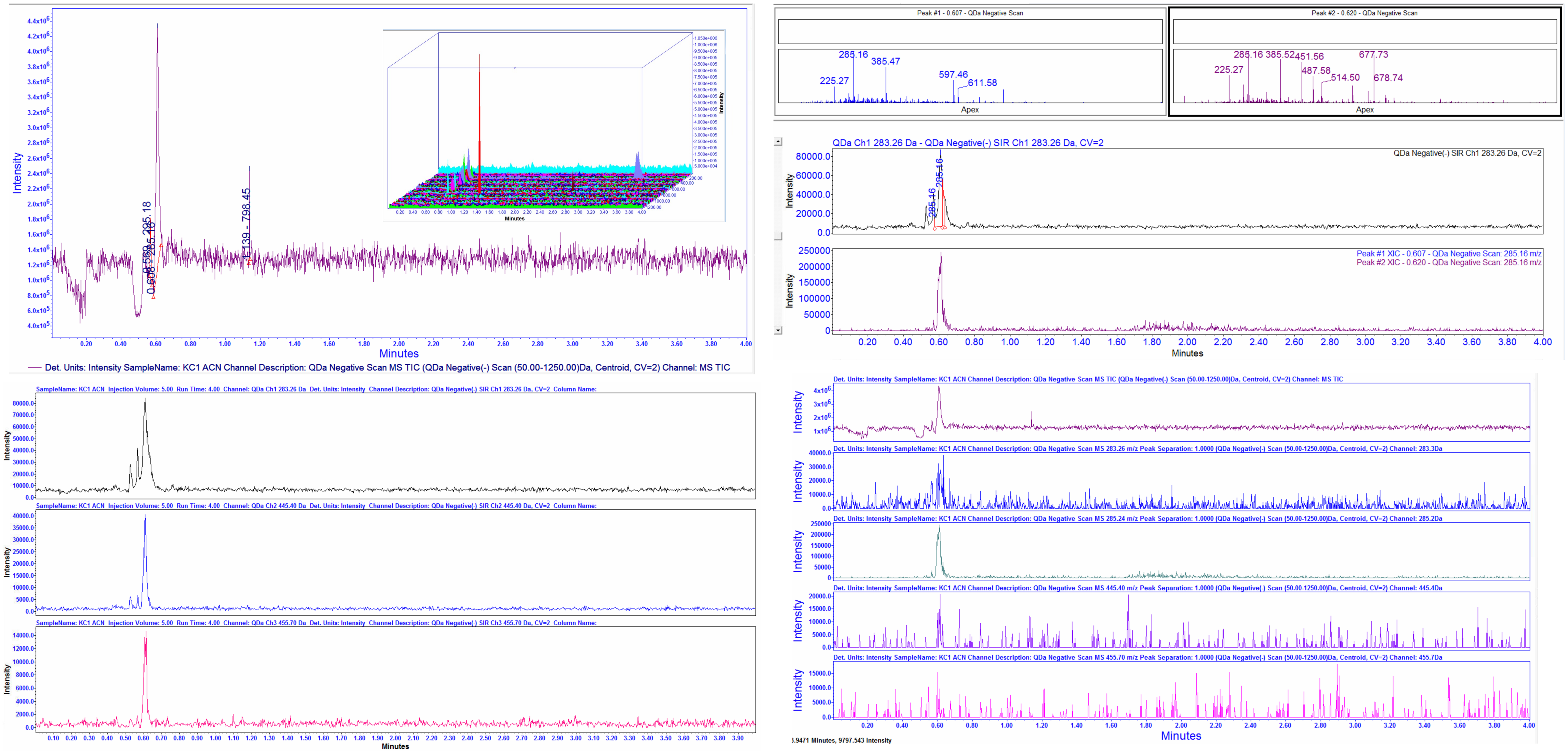

Figure S36: TIC and SIR experiment, negative mode (ESI-) 1) Acacetin 283.26 Da 2)Luteolin 28.24 Da 3) Tilianin 445.4 Da 4) Ursolic acid 455.41 Da [the X-axis represents time in minutes, and Y-axis represents signal intensity]. Column ACQUITY UPLC® HBE C18 130 Å column (1.7 μm, 2.1 x 50mm).

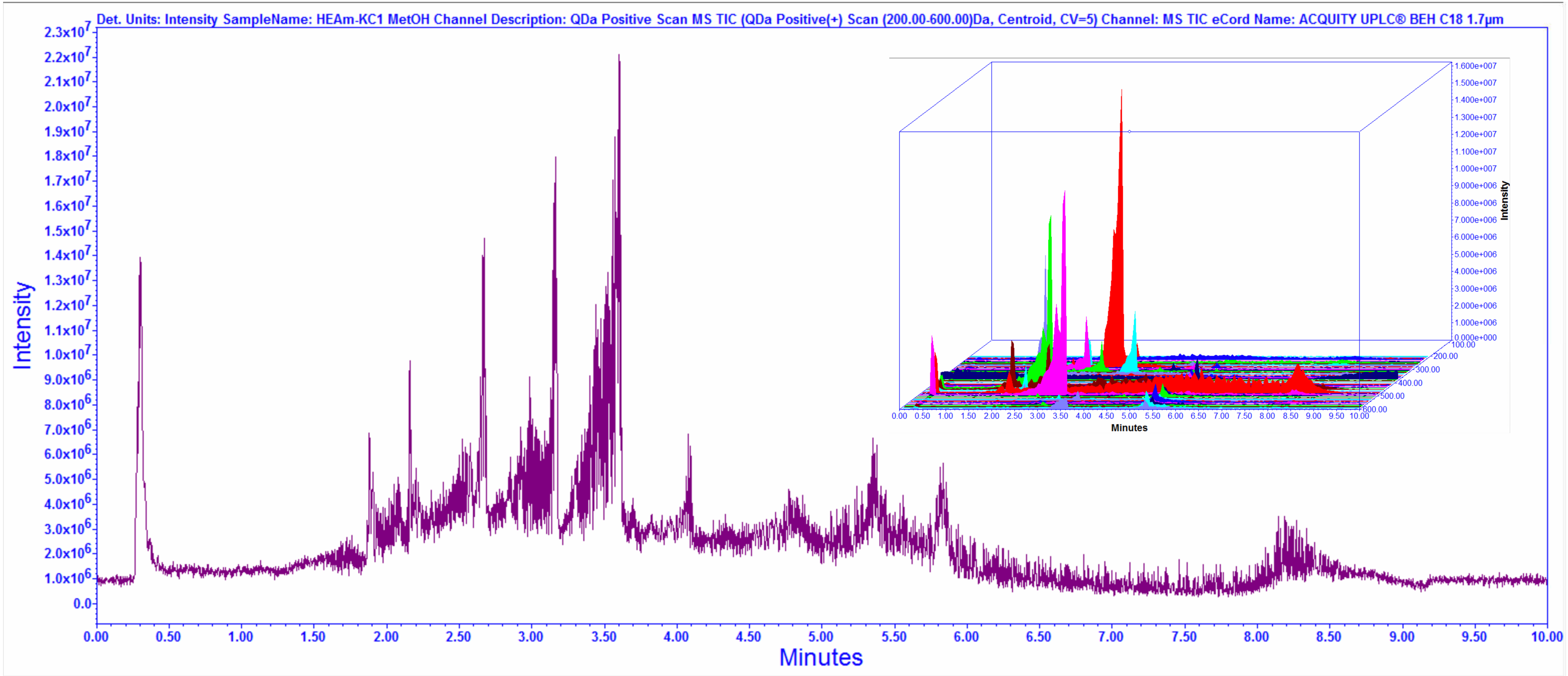

Figure S37: total ion chromatogram, exploratory mass scan from 200-600 Da **[ESI+],** the X-axis represents time in minutes, and Y-axis represents signal intensity].Gradient elution, column ACQUITY UPLC® HBE C18 130 Å column (1.7 µm, 2.1 x 50mm).

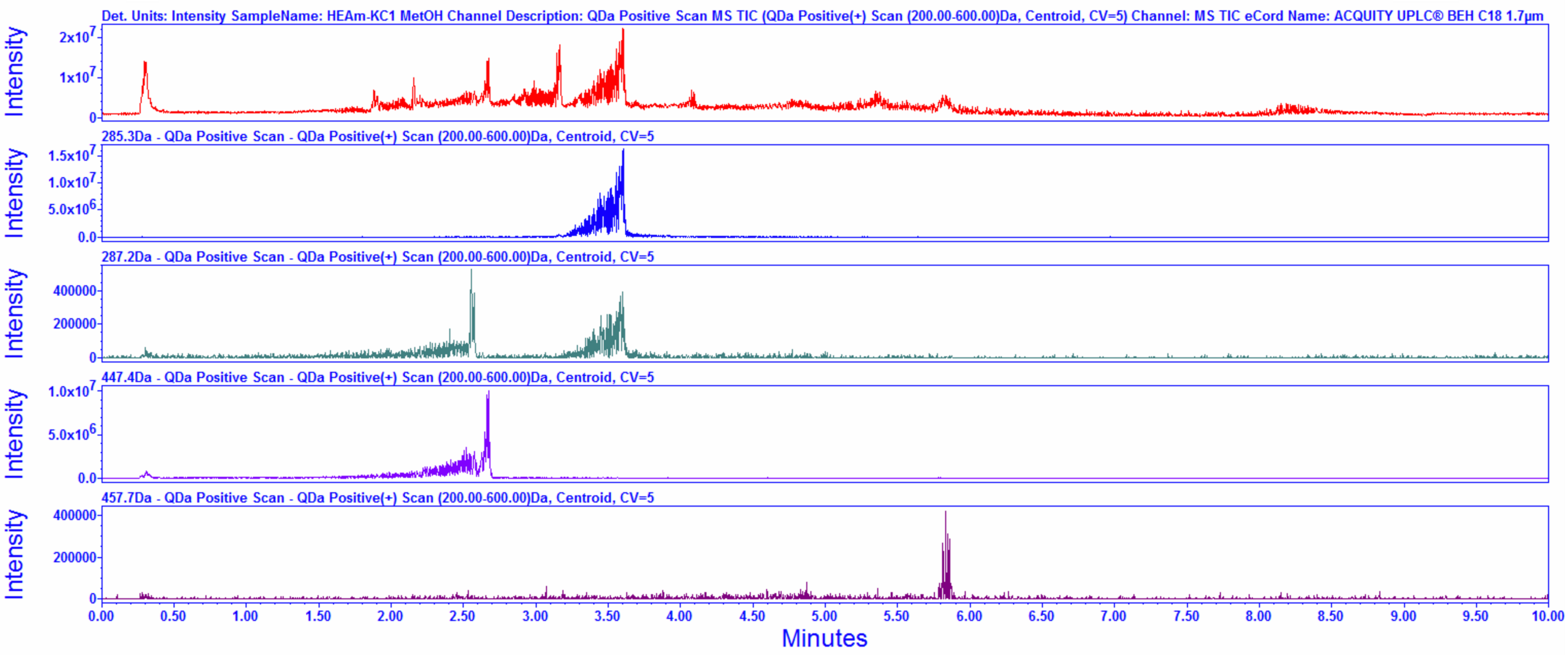

Figure S38: Stack plot from Mass Scan in positive mode (ESI+) for HAE*AmA*) Total ion chromatogram, exploratory mass scan from 90-800 Da Selected channels for B) **Acacetin** 285.26 Da m/z [M+1] **C) Luteolin** 287.2 Da m/z [M+1] **D) Tilianin** 447.4 Da m/z [M+1] **E) Oleanolic** and **ursolic acid** 457.7 Da m/z [M+1]. The X-axis represents time in minutes, and Y-axis represents signal intensity; chromatographic column ACQUITY UPLC® HSS T3 130 Å (1.8 µm, 2.1 × 100 mm).
